# Adapting the Scar-in-a-Jar to Skin Fibrosis and Screening Traditional and Contemporary Anti-Fibrotic Therapies

**DOI:** 10.3389/fbioe.2021.756399

**Published:** 2021-10-26

**Authors:** João Q. Coentro, Ulrike May, Stuart Prince, John Zwaagstra, Olli Ritvos, Tero A.H. Järvinen, Dimitrios I. Zeugolis

**Affiliations:** ^1^ Regenerative, Modular and Developmental Engineering Laboratory (REMODEL) and Science Foundation Ireland (SFI) Centre for Research in Medical Devices (CÚRAM), National University of Ireland Galway (NUI Galway), Galway, Ireland; ^2^ Faculty of Medicine and Health Technology, Tampere University, Tampere, Finland; ^3^ Human Health Therapeutics Research Centre, National Research Council Canada, Montreal, QC, Canada; ^4^ University of Helsinki, Helsinki, Finland; ^5^ Tampere University Hospital, Tampere, Finland; ^6^ Regenerative, Modular and Developmental Engineering Laboratory (REMODEL), Charles Institute of Dermatology, Conway Institute of Biomolecular and Biomedical Research and School of Mechanical and Materials Engineering, University College Dublin (UCD), Dublin, Ireland

**Keywords:** *In vitro* tools, drug testing, disease modelling, macromolecular crowding, fibrosis, anti-fibrotic molecules

## Abstract

Skin fibrosis still constitutes an unmet clinical need. Although pharmacological strategies are at the forefront of scientific and technological research and innovation, their clinical translation is hindered by the poor predictive capacity of the currently available *in vitro* fibrosis models. Indeed, customarily utilised *in vitro* scarring models are conducted in a low extracellular matrix milieu, which constitutes an oxymoron for the in-hand pathophysiology. Herein, we coupled macromolecular crowding (enhances and accelerates extracellular matrix deposition) with transforming growth factor *β*1 (TGF*β*1; induces trans-differentiation of fibroblasts to myofibroblasts) in human dermal fibroblast cultures to develop a skin fibrosis *in vitro* model and to screen a range of anti-fibrotic families (corticosteroids, inhibitors of histone deacetylases, inhibitors of collagen crosslinking, inhibitors of TGF*β*1 and pleiotropic inhibitors of fibrotic activation). Data obtained demonstrated that macromolecular crowding combined with TGF*β*1 significantly enhanced collagen deposition and myofibroblast transformation. Among the anti-fibrotic compounds assessed, trichostatin A (inhibitors of histone deacetylases); serelaxin and pirfenidone (pleiotropic inhibitors of fibrotic activation); and soluble TGF*β* receptor trap (inhibitor of TGF*β* signalling) resulted in the highest decrease of collagen type I deposition (even higher than triamcinolone acetonide, the gold standard in clinical practice). This study further advocates the potential of macromolecular crowding in the development of *in vitro* pathophysiology models.

## Introduction

Skin fibrosis is characterised by the formation of excessive fibrous connective tissue, which leads to alteration of the architecture of the dermis and compromises skin’s function and mechanical properties (Coentro et al., 2018). Skin fibrosis manifests either locally (after skin wounding) or systemically (as a result of autoimmune skin disease), with clinical outcomes ranging from small cosmetic imperfections to functional impairment. Skin fibrosis affects over 100 million patients every year (Sund and Arrow, 2000) and is associated with annual healthcare expenditure in excess of US$ 12 billion in the US alone (Griffin et al., 2020).

Fibrosis and skin wound-related scarring are complex, multi-stage (inflammatory, proliferative and remodelling) processes, involving numerous cells, molecules and signalling pathways (Zeng et al., 2011). The key feature in fibrosis formation is the transformation of normal fibroblasts to myofibroblasts, which are contraction capable cells and responsible for scar and fibrosis formation in different diseases (Schulz et al., 2018; Pakshir et al., 2020). *De novo* expression of *α* smooth muscle actin (*α*SMA), a marker of late stage myofibroblast transformation (Pakshir et al., 2020), is ultimately associated with fibrosis. Biological (e.g., transforming growth factor *β*1, TGF*β*1) and biophysical (e.g., mechanical stress (Seo et al., 2020)) stimuli trigger fibroblast transition into myofibroblast lineage (Hinz et al., 2012), which is associated with the establishment of several characteristic hallmarks of fibrosis, such as atypical collagen synthesis and deposition, alterations in collagen type I/III ratio and distorted extracellular matrix (ECM) architecture (Jarvinen and Ruoslahti, 2010; Henderson et al., 2020; Pakshir et al., 2020).

Anti-fibrotic therapeutics are the first line of defence in scar-wars (Jarvinen and Ruoslahti, 2010; Desallais et al., 2014). Different classes of molecules have been assessed over the years, largely classified as: corticosteroids; inhibitors of histone deacetylases, collagen crosslinking and deposition, TGF*β* signalling or pleiotropic fibrotic activation (Supplementary Table S1). Unfortunately, the development of anti-fibrotic approaches has been hindered by side effects encountered. For example, TGF*β* inhibitors may compromise immunity and induce autoimmune diseases (Henderson et al., 2020). Other potential factors that have further limited the development of anti-fibrotic therapies include the use of time consuming and low throughput and specificity (due to genetic, epigenetic, immune status and physiological differences between humans and animals) *in vivo* models that fail to recapitulate human disease states and effectively screen potential drugs (Padmanabhan et al., 2019). *In vitro* models have their share of shortcomings (Supplementary Table S2). For example, the low ECM levels present in many traditional *in vitro* models (an oxymoron for a fibroplasia model) is liable for cell genetic and epigenetic drift and restrains/inhibits cell-ECM interactions and paracrine signalling cascades, resulting in failure of the models to predict *in vitro* relevant *in vivo* toxicity of the under investigation molecules (Chen et al., 2009).

Macromolecular crowding (MMC), a biophysical technique based on volume exclusion effect, accelerates the enzymatic conversion of water-soluble procollagen to insoluble collagen resulting in enhanced and accelerated collagen type I and associated ECM deposition (Raghunath and Zeugolis, 2021; Tsiapalis and Zeugolis, 2021; Zeugolis, 2021). In 2009, the first pathophysiologically relevant *in vitro* fibrosis model (termed *Scar-in-the-Jar*) was published that utilised the principles of MMC (to enhance and accelerate ECM deposition) and TGF*β*1 (to induce myofibroblast transformation of WI-38 lung fibroblasts) (Chen et al., 2009). Since then, several fibrotic models based on MMC have been developed for screening anti-fibrotics in different fibrotic diseases (e.g., dermal (Fan et al., 2019; Fan et al., 2020), lung (Good et al., 2019; Rønnow et al., 2020), vocal fold (Graupp et al., 2015; Graupp et al., 2018) scarring). Unfortunately, these dermal scar models might be incomplete as the optimal crowding molecule was not used (Chen et al., 2009). Although MMC agents, such as Ficoll^®^ (Fan et al., 2019) and polyvinylpyrrolidone (Rashid et al., 2014), have been used as crowding agents, dextran sulphate has demonstrated pro-fibrotic potency by transforming corneal fibroblasts to myofibroblasts (Kumar et al., 2015), possibly due to its binding and releasing capacity of growth factors, such as TGF*β*1 (Walton, 1952; Logeart-Avramoglou et al., 2002; Maire et al., 2005).

Considering the above, herein, we first modified and adopted the *Scar-in-the-Jar* model (Chen et al., 2009; Stebler and Raghunath, 2021) for skin fibrosis by using dextran sulphate as MMC agent, primary dermal fibroblasts as tissue-specific cell population and TGF*β*1 to induce their myofibroblast trans-differentiation (Supplementary Figure S1). We then assessed the model’s anti-fibrotic screening potential (through collagen deposition and cell metabolic activity, DNA concentration and viability) by using different anti-fibrotic compounds (corticosteroids: Triamcinolone acetonide, TAC; inhibitors of histone deacetylases: Trichostatin A, TSA; inhibitors of collagen crosslinking: *β*-aminopropionitrile, BAPN; inhibitors of TGF*β* signalling: soluble TGF*β* type II receptor-based 2 traps, recombinant proteins T22d35 and T122bt and an activin IIB receptor inhibitor, ACVR2B; and pleiotropic inhibitors of fibrotic activation: Serelaxin, RLX-2 and Pirfenidone, Pirf).

## Materials and Methods

### Materials

All labware were obtained from Sarstedt (Ireland) and Thermo Fisher Scientific (Ireland) and all chemicals and reagents were purchased from Sigma-Aldrich (Ireland), unless stated otherwise.

### Recombinant Protein Production, Purification and Analysis

A TGF*β* type II receptor-based (TβRII)2, single-chain trap was designed, termed T22d35, where two TβRII ligand binding domains are separated by a 35 amino acid long native linker (Zwaagstra et al., 2012). In addition, we also created a heterovalent trap, termed T122bt, where the TβRI domain was added to two TβRII ligand binding domains separated by a 60 amino acid long native linker (O'Connor-Mccourt et al., 2013) (Supplementary Figure S2). Both TGFβ traps were expressed in a mammalian expression system, purified by chromatography and characterized in detail. TGF*β* neutralisation curves were plotted, and the determined IC_50_-values were tabulated (Supplementary Table S3).

### Cell Culture and Fibrotic Model Induction

Normal adult dermal fibroblasts (DF, PCS-201-012, ATCC, United States) were routinely sub-cultured and used between passages 3 and 6, with DMEM supplemented with 10% FBS and 1% penicillin/streptomycin; media were changed every 2–3 days. For the various experiments, cells were cultured at 25,000 cells/cm^2^ and allowed to attach for 24 h, after which the culture media were changed to media containing 100 *μ*M L-ascorbic acid 2-phosphate sesquimagnesium salt hydrate, 100 *μ*g/ml 500 kDa Dextran Sulphate (DxS), 5 ng/ml TGF*β*1 and in combination with or without the following anti-fibrotic substances: BAPN (Acros Organics, Belgium, 0.1, 0.25, 0.5 and 1 mM) and TAC (0.025, 0.050, 0.1 and 0.2 mM) were dissolved in sterile 20% dimethyl sulfoxide solution and then were added into the media; TSA (0.5, 1, 2 and 5 *μ*M), RLX-2 (5, 10, 25 and 50 nM), Pirf (0.25, 0.5, 1 and 1.5 mM) ACVR2B (5, 10, 25 and 50 nM), T22d25 (25, 50, 100 and 200 nM) and T122bt (25, 50, 100 and 200 nM) were dissolved in supplemented media. Tested drug concentrations for different anti-fibrotic molecules were based on previously published data. From the review of the literature we chose reported concentration ranges that proved to have a therapeutic effect *in vitro* (1–20 μM for TAC (Cancela and Rebut-Bonneton, 1987; Carroll et al., 2002; Yang et al., 2018); 0.1–1 μM for TSA (Rombouts et al., 2002; Ghosh et al., 2007; Chen et al., 2009); 5–17 nM for RLX-2 (Unemori and Amento, 1990; Unemori et al., 1992; Samuel et al., 2003); 0.5–5.4 mM for Pirf (Saito et al., 2012; Hall et al., 2018; Wells and Leung, 2020); ∼IC50 TGF*β* neutralising values of 2.5–8.2 nM for T22d35 and T122bt (Zwaagstra et al., 2012; O'Connor-Mccourt et al., 2013), which were also used for ACVR2B; and 0.1–1 mM for BAPN (Redden and Doolin, 2003; Péterszegi et al., 2008; Chen et al., 2009)). The anti-fibrotic substances were added to the culture media only once. Supplemented media were changed every 3 days and cells were analysed at the appropriate time points.

### SDS-PAGE Analysis

Cell layers were analysed by SDS-PAGE as described elsewhere (Capella-Monsonís et al., 2018). Briefly, culture media were aspirated, cell layers were washed with PBS and digested with 0.1 mg/ml pepsin solution (porcine gastric mucosa, 3,500–4,200 U/mg) in 0.5 M acetic acid. The cell layers were then scraped, neutralised with 1 M NaOH, denatured at 95°C and resolved under non-reducing conditions using in-house resolving and stacking polyacrylamide gels (5 and 3% respectively) on a Mini-Protean 3 (Bio-Rad Laboratories, United Kingdom) system. Purified collagen type I (Symatese, France) was used as standard. Samples were stained using a SilverQuest™ Silver Staining Kit (Invitrogen, Ireland) according to the manufacturer’s instructions. Densitometric analysis was performed on *α*1(I), *α*2(I), *β*11(I), *β*12(I) or *γ*(I) bands, as appropriate, using ImageJ software (NIH, United States).

### Immunocytochemistry Analysis

Cells layers were washed with PBS, fixed with 4% paraformaldehyde and permeabilised with 0.25% Triton X-100. Cells layers were then blocked with 5% donkey serum in PBS for 1 h at room temperature and incubated with primary antibodies [rabbit *α*-human collagen type I 1:300: PA2140-2 (Boosterbio, United States); mouse *α-*human *α*SMA 1:300: ab7817 (Abcam, United Kingdom)] for a minimum of 90 min at room temperature. Cell layers were then washed 3 times with PBS and incubated with appropriate secondary antibodies (Alexa Fluor™ 594 donkey anti-rabbit 1:500: R37119 or Alexa Fluor™ 488 donkey anti-mouse 1:400: R37114; both from Thermo Fisher Scientific, United States) for 60 min. Cell nuclei were counterstained with 4,6-diamidino-2-phenylindole (DAPI) for 5 min and washed 3 times with PBS. Cells layers were then imaged with an inverted fluorescence microscope (Olympus IX-81, Olympus Corporation, Japan) and further processed with ImageJ software (NIH, United States).

### DNA Concentration Analysis

Cell proliferation was assessed using the Quant-iT™ PicoGreen™ dsDNA Assay Kit for quantifying DNA concentration (Invitrogen, United States) as per the manufacturer’s instructions. Briefly, cells were washed with PBS, ultrapure water was added and three cycles of freezing and thawing to promote cell lysis were followed. DNA standards of known concentrations were prepared, both samples and standards were mixed with Tris-HCl-Ethylenediaminetetraacetic acid (EDTA) buffer and PicoGreen™ reagent and incubated in dark. Fluorescence was measured at 480 nm excitation and 520 nm emission with a Varioskan Flash Spectral scanning multimode reader (Thermo Fisher Scientific, United States).

### Metabolic Activity Analysis

Cell metabolic activity was assessed using the alamarBlue^®^ assay (ThermoFisher Scientific, United States) as per the manufacturer’s instructions. Briefly, cells were washed with PBS and incubated with a 10% alamarBlue^®^ solution in PBS for 3 h at 37°C in a humidified atmosphere of 5% CO_2_. Absorbance was then measured at 550 and 595 nm with a Varioskan Flash Spectral scanning multimode reader (Thermo Fisher Scientific, United States). Cell metabolic activity was expressed as percentage reduction of the alamarBlue^®^ dye and normalised by the respective quantity of DNA and to the non-treated control.

### Viability Analysis

Calcein AM (live cell marker) and ethidium homodimer I (dead cell marker) stainings were used to assess the influence of MMC, TGFβ1 and anti-fibrotic molecule supplementation on cell viability. Briefly, at each time point, cells were carefully washed with PBS and incubated with a solution of calcein AM (4 μM) and ethidium homodimer I (2 μM) in PBS for 30 min at 37°C in a humidified atmosphere of 5% CO_2_. Afterwards, cells were imaged with an inverted fluorescence microscope Olympus IX-81 (Olympus Corporation, Japan), using the FITC filter for calcein AM and the Texas Red filter for ethidium homodimer.

### Statistical Analysis

Statistical evaluation of the data was performed using the statistical program MiniTab^®^ version 17 (Minitab Inc., United States). All data are expressed as mean values ± standard deviations. Datasets were assessed for normal distribution (Anderson-Darling) and equal variance (Levene’s test for homogeneity of variances). When the assumptions of parametric analysis were confirmed, one-way analysis of variance (ANOVA) was used for multiple comparisons and Tukey’s post hoc test was used for pairwise comparisons. When either or both assumptions were violated, non-parametric analysis was conducted using Kruskall-Wallis test for multiple comparisons and Mann-Whitney U test for pairwise comparisons. Statistical significance was accepted at *p* < 0.05.

## Results

### Fibrotic Model Establishment

**FIGURE 1:**
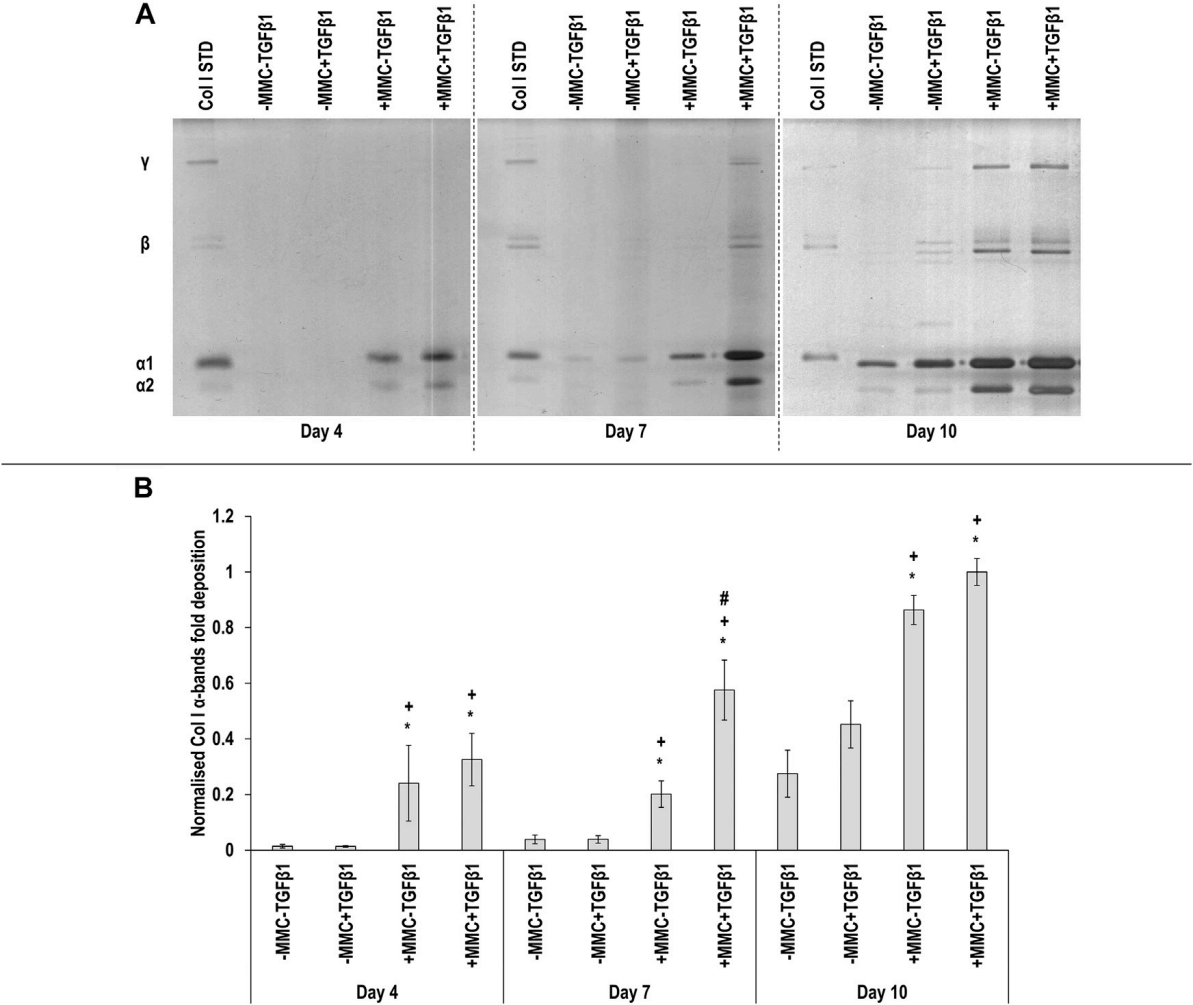
Macromolecular crowding and TGFβ1 increase collagen deposition. Adult dermal fibroblasts were cultured in the presence of dextran sulphate and TGFβ1 for up to 10 days. SDS-PAGE **(A)** and densitometry of α(I)1 and α(I)2 bands **(B)** analyses revealed that MMC increased collagen type I deposition and MMC coupled with TGFβ1 increased further collagen type I deposition. Col I STD: 0.1 mg/ml. One-way ANOVA and Tukey’s post-hoc analysis for pairwise comparisons were conducted. *: *p* < 0.05 indicates a statistically significant difference when compared to the negative control (-MMC-TGFβ1) of the respective time point. +: *p* < 0.05 indicates a statistically significant difference when compared to the -MMC+TGFβ1 group of the respective time point. #: *p* < 0.05 indicates a statistically significant difference when compared to the +MMC-TGFβ1 group of the respective time point. *n* = 3.

SDS-PAGE (Figure 1A) and complementary densitometry analysis of collagen type I α(I)1 and α(I)2 bands (Figure 1B) made apparent that at day 4 and day 7 almost no collagen was deposited in the control and the TGF*β*1 groups, whilst MMC groups significantly (*p* < 0.05) increased collagen deposition at all time points, which was further increased (*p* < 0.05) with +MMC+TGF*β*1 at day 7. Densitometry analysis also showed a significant increase (*p* < 0.05) of *β*11(I), *β*12(I) dimers for the +MMC+TGF*β*1 group at day 4 and day 7 (Supplementary Figure S3A) and of γ(I) trimers for the +MMC+TGF*β*1 group at day 4 and day 10 (Supplementary Figure S3B).

**FIGURE 2:**
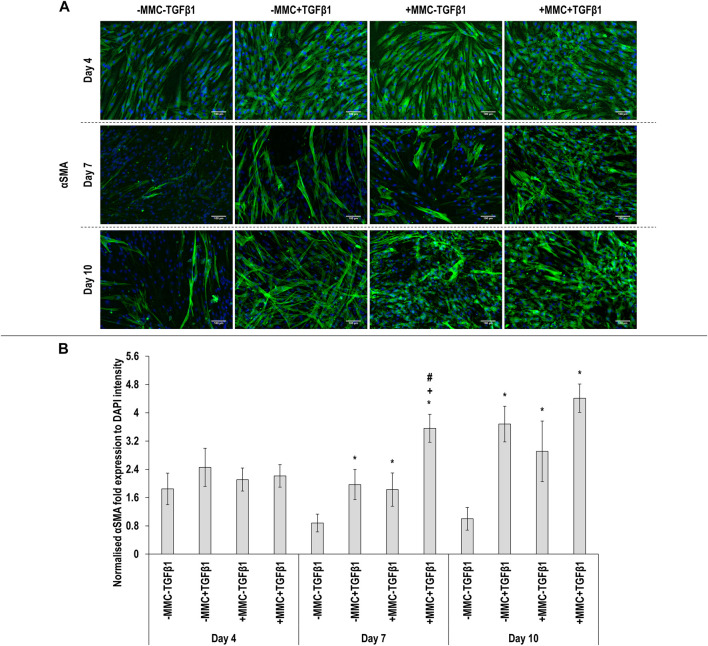
Macromolecular crowding and TGFβ1 increase αSMA expression. Immunocytochemistry **(A)** and image intensity analysis for αSMA **(B)** revealed no differences in αSMA expression between the groups at day 4; the +MMC+TGFβ1 group induced the highest αSMA expression at day 7; the -MMC-TGFβ1 group induced the lowest αSMA expression at day 10. One-way ANOVA and Tukey’s post-hoc comparison test or Kruskal Wallis and Mann Whitney post-hoc analyses were conducted. *: *p* < 0.05 indicates a statistically significant difference when compared to the negative control (-MMC-TGFβ1) of the respective time point. +: *p* < 0.05 indicates a statistically significant difference when compared to the -MMC+TGFβ1 group of the respective time point. #: *p* < 0.05 indicates a statistically significant difference when compared to the +MMC-TGFβ1 group of the respective time point. *n* = 3.

**FIGURE 3:**
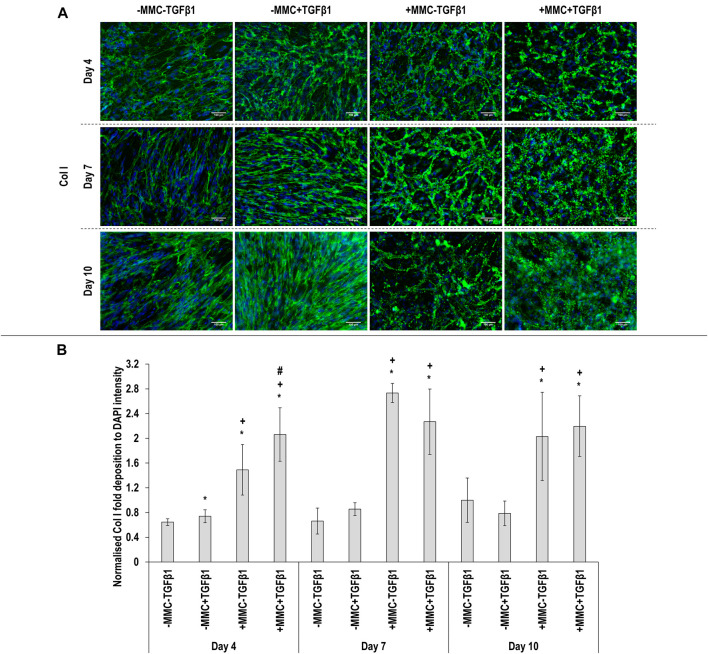
Macromolecular crowding and TGFβ1 increase collagen deposition. Immunocytochemistry **(A)** and image intensity analysis for collagen type I **(B)** revealed that the +MMC-TGFβ1 and +MMC+TGFβ1 groups induced the highest collagen deposition at all time points. One-way ANOVA and Tukey’s post-hoc comparison test or Kruskal Wallis and Mann Whitney post-hoc analyses were conducted. *: *p* < 0.05 indicates a statistically significant difference when compared to the negative control (-MMC-TGFβ1) of the respective time point. +: *p* < 0.05 indicates a statistically significant difference when compared to the -MMC+TGFβ1 group of the respective time point. #: *p* < 0.05 indicates a statistically significant difference when compared to the +MMC-TGFβ1 group of the respective time point. *n* = 3.

Immunocytochemistry (Figure 2A for *α*SMA and Figure 3A for collagen type I) analysis made apparent that when the cells were cultured with +MMC+TGF*β*1, clear stress fibres were observed and the collagen fibres were aligned parallel to the stress fibres, albeit collagen type I deposition showed a granular pattern when MMC was used, whilst in its absence, a meshwork architecture was evidenced. Complementary image intensity (Figure 2B for *α*SMA and Figure 3B for collagen type I) analyses revealed that, in comparison to the control, the addition of TGF*β*1 resulted in a significant (*p* < 0.05) increase in *α*SMA expression at day 7 and day 10 and collagen type I deposition at day 4. MMC resulted in a significant (*p* < 0.05) increase compared to the control for both molecules in almost all time points. This was also observed for +MMC+TGF*β*1, which led to an even greater (*p* < 0.05) increase in *α*SMA at day 7 and collagen I at day 4.

### Screening of Anti-Fibrotic Molecules in the *in vitro* Fibrotic Model

**FIGURE 4:**
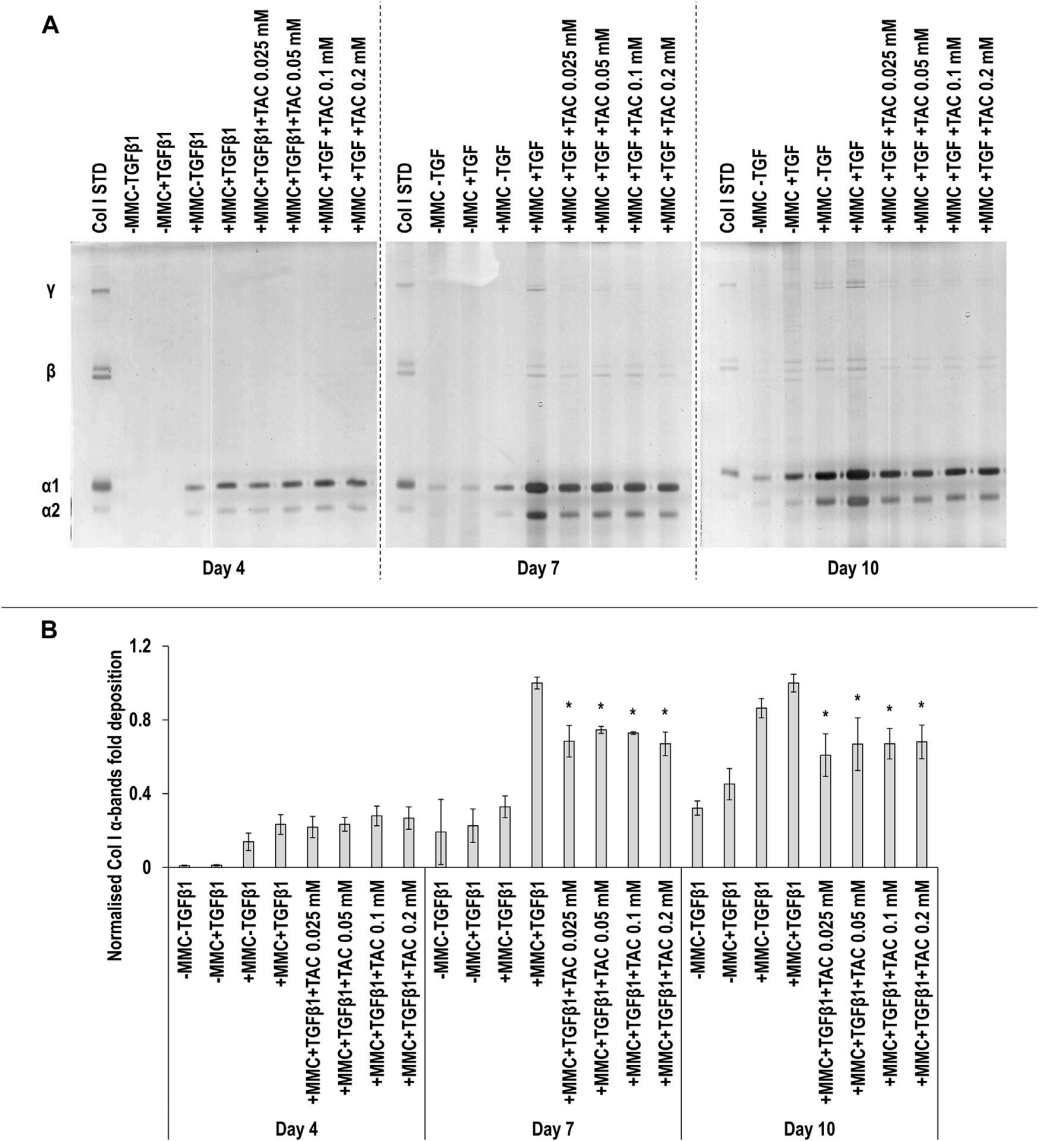
TAC moderately reduces collagen deposition. All TAC concentrations resulted in reduced collagen deposition at day 7 and day 10, in comparison to +MMC+TGF*β*1 group, as judged by SDS-PAGE **(A)** and densitometry of α(I)1 and α(I)2 bands analyses **(B)**. Col I STD: 0.1 mg/ml. One-way ANOVA and Tukey’s post-hoc comparison tests were conducted. *: *p* < 0.05 denotes a significant difference when compared to the +MMC+TGF*β*1 group of the respective time point. *n* = 3.

SDS-PAGE (Figure 4A) and densitometry analysis of α(I)1 and α(I)2 bands (Figure 4B) revealed that all TAC concentrations resulted in significant (*p* < 0.05) decrease of α(I)1 and α(I)2 chains deposition at day 7 and day 10, when compared to the +MMC+TGF*β*1 group at the respective time point. No significant (*p* < 0.05) differences in the deposition of *β*11(I), *β*12(I) dimers (Supplementary Figure S4A) and γ(I) trimers (Supplementary Figure S4B) were observed. At day 7 the +MMC+TGF*β*1+TAC groups exhibited significantly (*p* < 0.05) higher and at day 10 significantly (*p* < 0.05) lower DNA concentration than the +MMC+TGF*β*1 group (Supplementary Figure S5A). At day 4 and 7 almost all the +MMC+TGF*β*1+TAC groups exhibited significantly (*p* < 0.05) lower and at day 10 significantly (*p* < 0.05) higher metabolic activity than the +MMC+TGF*β*1 group (Supplementary Figure S5B).

**FIGURE 5:**
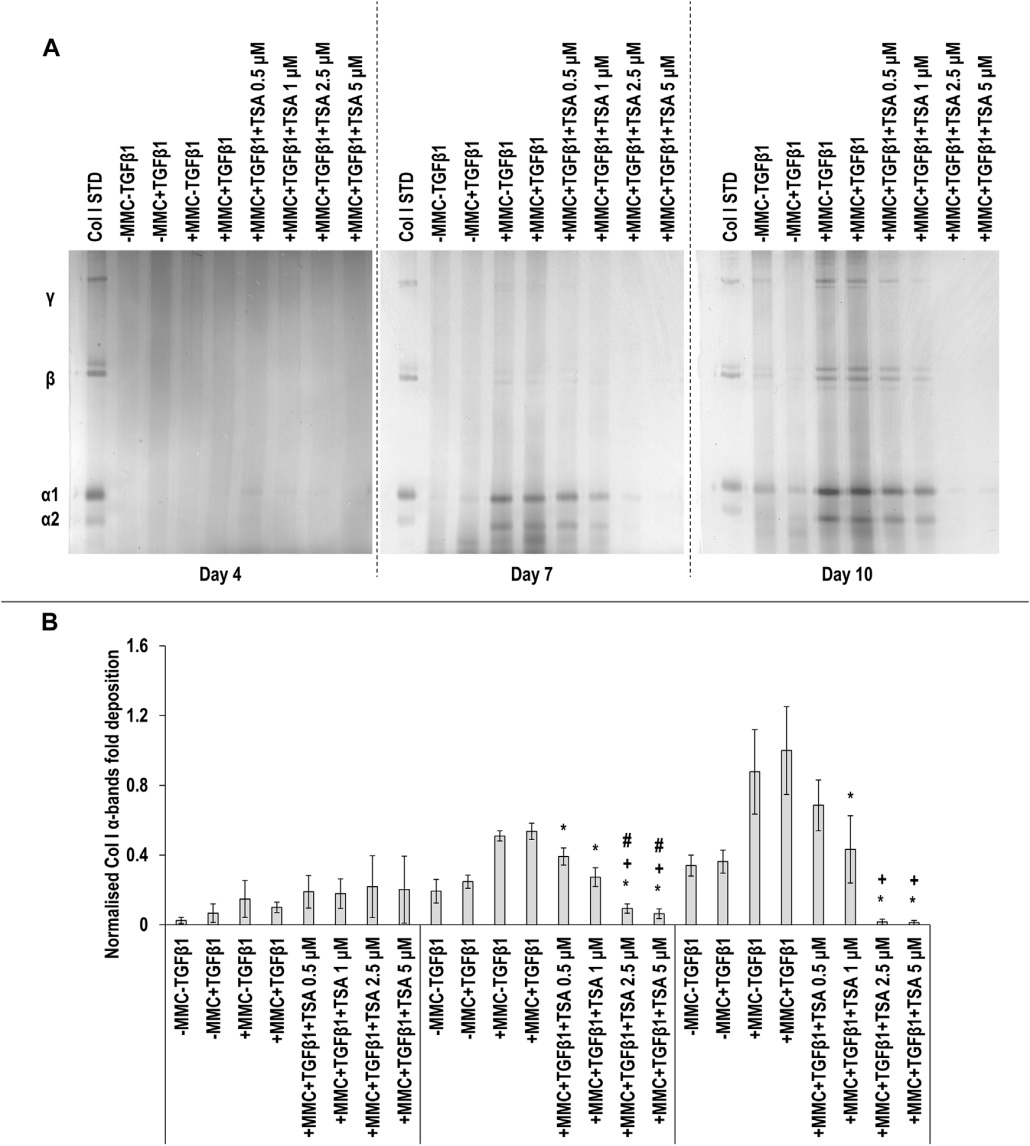
High concentrations of TSA reduce collagen deposition. The 2.5 and 5 µM TSA concentrations in +MMC+TGF*β*1 at day 7 and day 10 induced the lowest collagen deposition in comparison to +MMC+TGF*β*1 group, as revealed by SDS-PAGE **(A)** and densitometry of α(I)1 and α(I)2 bands **(B)**. Col I STD: 0.1 mg/ml. One-way ANOVA and Tukey’s post-hoc comparison tests were conducted. *: *p* < 0.05 indicates a statistically significant difference when compared to the +MMC+TGF*β*1 group of the respective time point. +*: p* < 0.05 indicates a statistically significant difference when compared to the +MMC+TGF*β*1+TSA 0.5 μM group of the respective time point. #*: p* < 0.05 indicates a statistically significant difference when compared to the +MMC+TGF*β*1+TSA 1 μM group of the respective time point. n = 3.

SDS-PAGE (Figure 5A) and densitometry of α(I)1 and α(I)2 bands (Figure 5B) analyses revealed that all TSA concentrations in +MMC+TGF*β*1 at day 7 and the 1, 2.5 and 5 µM TSA concentrations in +MMC+TGF*β*1 at day 10 resulted in significant (*p* < 0.05) decrease of α(I)1 and α(I)2 chains deposition, when compared to the +MMC+TGF*β*1 group at the respective time point. The 2.5 and 5 µM TSA concentrations in +MMC+TGF*β*1 at day 7 and all concentrations of TSA in +MMC+TGF*β*1 at day 10 resulted in significant (*p* < 0.05) decrease of *β*11(I), *β*12(I) dimers (Supplementary Figure S6A) and γ(I) trimers deposition (Supplementary Figure S6B). The 1, 2.5 and 5 µM TSA concentrations in +MMC+TGF*β*1 resulted in significant (*p* < 0.05) reduction of DNA concentration at day 4 and the 2.5 and 5 µM TSA concentrations in +MMC+TGF*β*1 resulted in significant (*p* < 0.05) reduction of DNA concentration at day 7 and day 10, all in comparison to the +MMC+TGF*β*1 group (Supplementary Figure S7A). The 0.5 and 2.5 µM TSA concentrations in +MMC+TGF*β*1 resulted in significant (*p* < 0.05) reduction of metabolic activity at day 7 in comparison to the +MMC+TGF*β*1 group (Supplementary Figure S7B).

**FIGURE 6:**
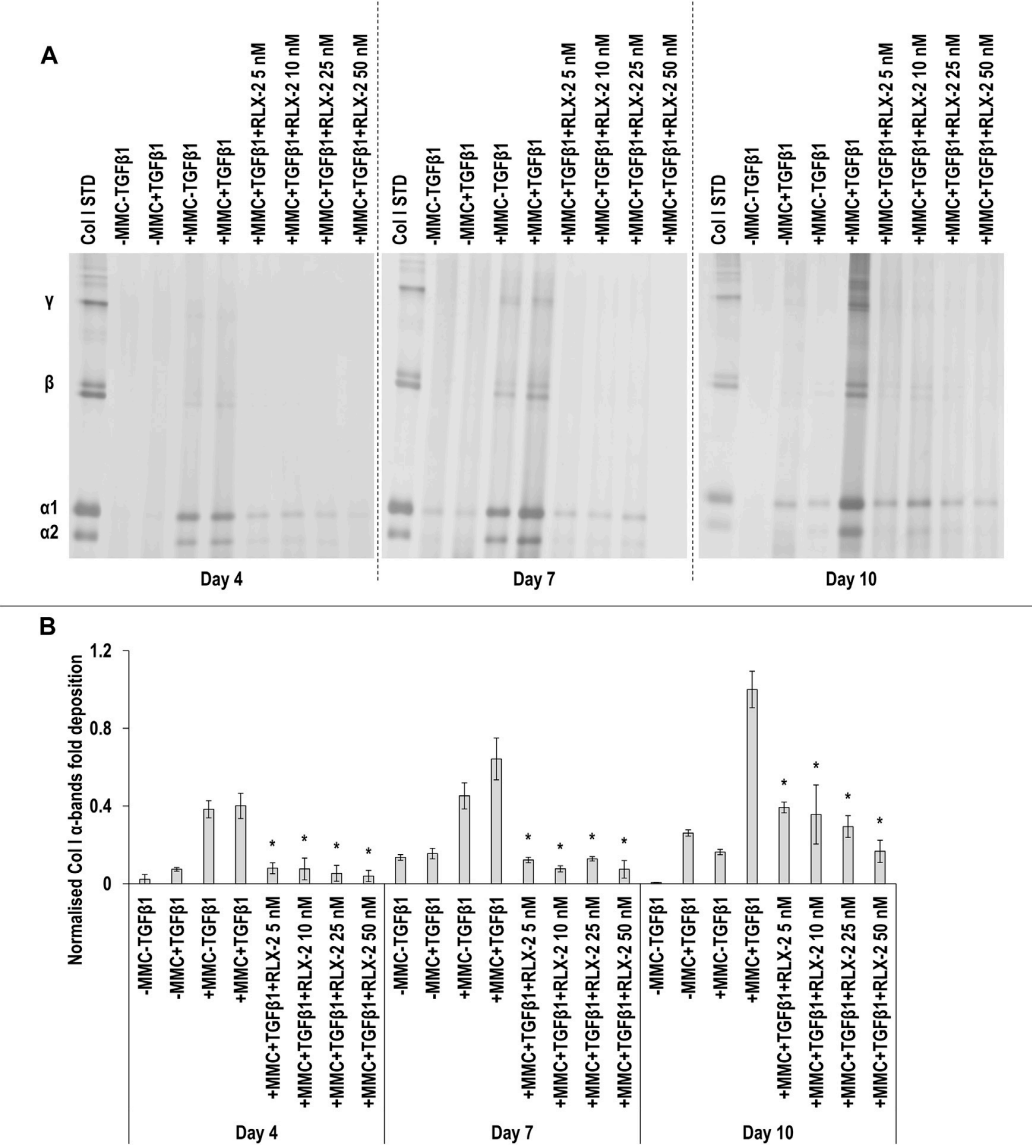
RLX-2 reduces collagen deposition at all time points. All RLX-2 concentrations in +MMC+TGF*β*1 at all time points resulted in significant (*p* < 0.05) decrease of collagen deposition, as judged by SDS-PAGE **(A)** and densitometry analysis of α(I)1 and α(I)2 bands **(B)**. Col I STD: 0.1 mg/ml. One-way ANOVA and Tukey’s post-hoc comparison tests were conducted. *: *p* < 0.05 indicates a statistically significant difference when compared to the +MMC+TGF*β*1 group of the respective time point. *n* = 3.

SDS-PAGE (Figure 6A) and densitometry analysis of α(I)1 and α(I)2 (Figure 6B), *β*11(I), *β*12(I) (Supplementary Figure S8A) and γ(I) bands (Supplementary Figure S8B) revealed that all RLX-2 concentrations in +MMC+TGF*β*1 at all time points resulted in a significant (*p* < 0.05) decrease of the deposition of α(I)1 and α(I)2, *β*11(I), *β*12(I) and γ(I) components, when compared to the +MMC+TGF*β*1 group at the respective time point. At day 7, all concentrations of RLX-2 in +MMC+TGF*β*1 resulted in significant (*p* < 0.05) increase of DNA concentration in comparison to the +MMC+TGF*β*1 group (Supplementary Figure S9A). At day 7, all concentrations of RLX-2 in +MMC+TGF*β*1 resulted in significant (*p* < 0.05) decrease of metabolic activity in comparison to the +MMC+TGF*β*1 group (Supplementary Figure S9B).

**FIGURE 7:**
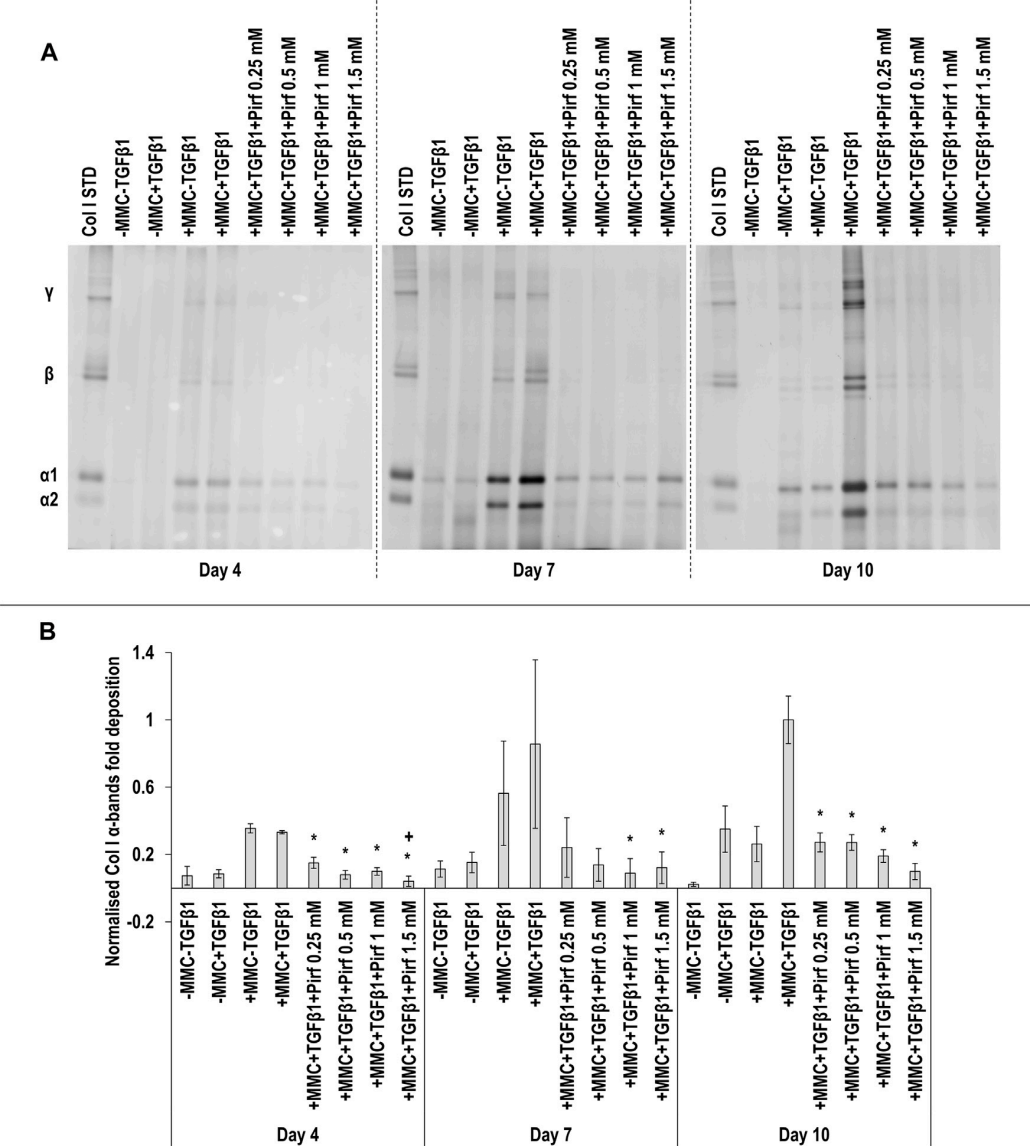
Pirf reduces collagen deposition. The 1 and 1.5 mM Pirf concentrations in +MMC+TGF*β*1 resulted in significant decrease of collagen deposition, as judged by SDS-PAGE **(A)** and densitometry analysis of α(I)1 and α(I)2 bands. Col I STD: 0.1 mg/ml. One-way ANOVA and Tukey’s post-hoc comparison tests were conducted. *: *p* < 0.05 indicates a statistically significant difference when compared to the +MMC+TGF*β*1 group of the respective time point. +: *p* < 0.05 indicates a statistically significant difference when compared to the +MMC+TGF*β*1+Pirf 0.25 mM group of the respective time point. *n* = 3.

SDS-PAGE (Figure 7A) and densitometry analysis of α(I)1 and α(I)2 bands (Figure 7B) revealed that all Pirf concentrations at day 4, the 1 and 1.5 mM Pirf concentrations at day 7 and all Pirf concentrations at day 10, all in +MMC+TGF*β*1, resulted in significant (*p* < 0.05) decrease of α(I)1 and α(I)2 chains deposition, all when compared to the +MMC+TGF*β*1 group. The 1 mM and the 1.5 mM Pirf concentrations at day 7 and all Pirf concentrations at day 10, all in +MMC+TGF*β*1, resulted in a significant (*p* < 0.05) decrease of *β*11(I), *β*12(I) dimer deposition, all when compared to the +MMC+TGF*β*1 group (Supplementary Figure S10A). All Pirf concentrations at day 4 and 10, all in +MMC+TGF*β*1, resulted in a significant (*p* < 0.05) decrease of γ(I) trimer deposition, all when compared to the +MMC+TGF*β*1 group (Supplementary Figure S10B). The 1.5 mM Pirf concentration in +MMC+TGF*β*1 resulted in a significant (*p* < 0.05) decrease of DNA concentration at day 4 and day 7, when compared to +MMC+TGF*β*1 group (Supplementary Figure S11A). The 1.5 mM Pirf concentration in +MMC+TGF*β*1 resulted in significant (*p* < 0.05) increase of metabolic activity at day 4 and day 7, when compared to +MMC+TGF*β*1 group (Supplementary Figure S11B).

**FIGURE 8:**
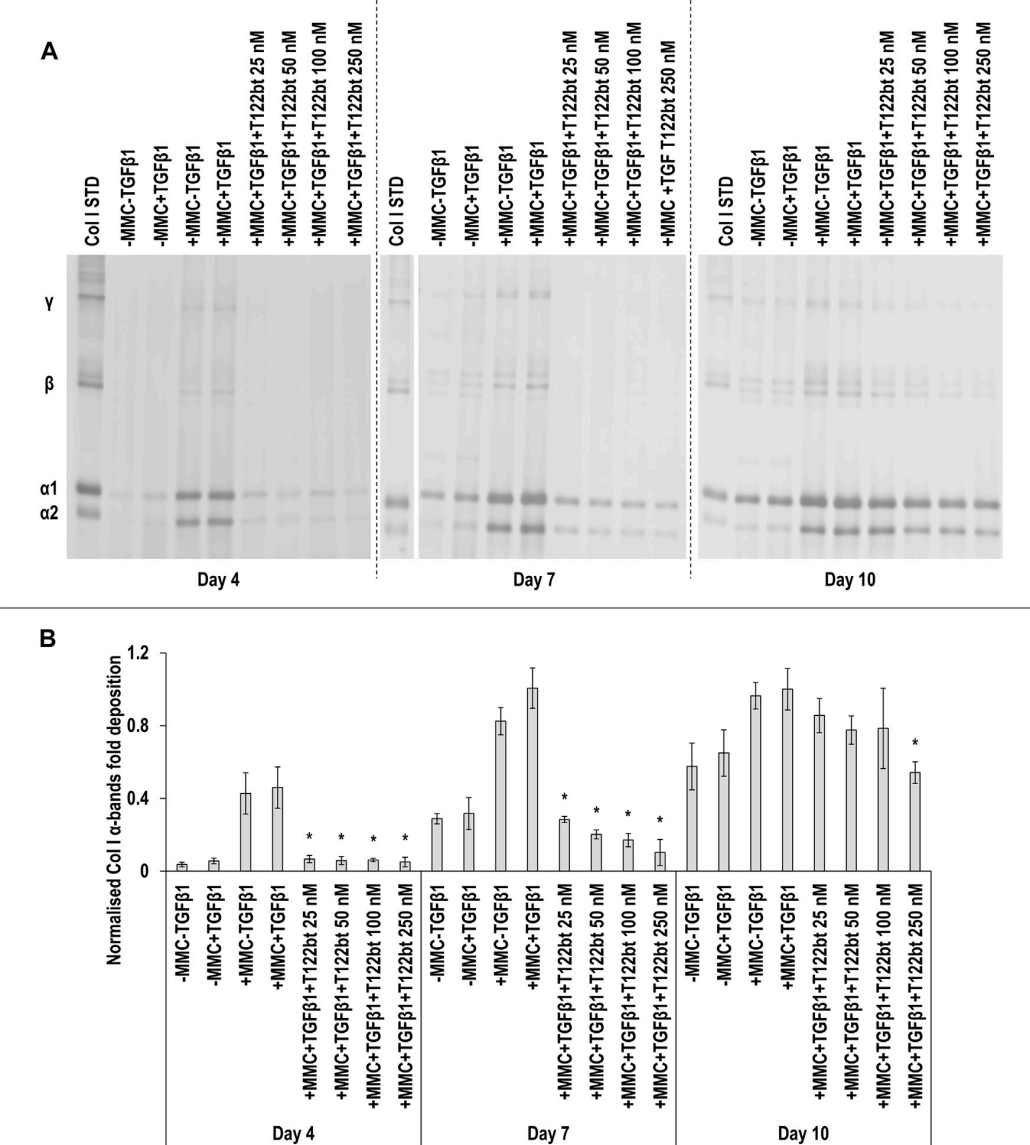
The highest concentrations of TGF*β*1 type II receptor-based trap (T122bt) reduce collagen deposition. The 100 and 250 nM T122bt concentrations in +MMC+TGF*β*1 significantly reduced collagen deposition at all time points, in comparison to +MMC+TGF*β*1 group, as judged by SDS-PAGE **(A)** and densitometry of α(I)1 and α(I)2 bands **(B)**. Col I STD: 0.1 mg/ml. One-way ANOVA and Tukey’s post-hoc comparison test or Kruskal Wallis and Mann Whitney post-hoc analyses were conducted, as appropriate. *: *p* < 0.05 indicates a statistically significant difference when compared to the +MMC+TGF*β*1 group of the respective time point. *n* = 3.

SDS-PAGE (Figure 8A) and densitometry analysis of α(I)1 and α(I)2 bands (Figure 8B) revealed that all T122bt concentrations in +MMC+TGF*β*1 at day 4 and day 7 and the 250 nM T122bt concentration in +MMC+TGF*β*1 at day 10 significantly (*p* < 0.05) reduced α(I)1 and α(I)2 chains deposition. Further densitometry analysis of *β*11(I), *β*12(I) bands (Supplementary Figure S12A) revealed that all T122bt concentrations in +MMC+TGF*β*1 at day 4 and day 7 and the 100 and 250 nM T122bt concentrations in +MMC+TGF*β*1 at day 10 significantly (*p* < 0.05) reduced *β*11(I), *β*12(I) dimer deposition. Additional densitometry analysis of γ(I) bands (Supplementary Figure S12B) revealed that all T122bt concentrations in +MMC+TGF*β*1 at all time points significantly (*p* < 0.05) reduced γ(I) trimer deposition.

No T122bt concentrations in +MMC+TGF*β*1 induced any significant (*p* < 0.05) differences in DNA concentration at any time point, in comparison to the +MMC+TGF*β*1 group (Supplementary Figure S13A). Only the 250 nM T122bt concentrations in +MMC+TGF*β*1 significantly (*p* < 0.05) increased metabolic activity, in comparison to the +MMC+TGF*β*1 group, at day 4 (Supplementary Figure S13B).

SDS-PAGE (Supplementary Figure S14A) and densitometry analysis of α (Supplementary Figure S14B), *β*11(I), *β*12(I) (Supplementary Figure S15A) and γ(I) bands (Supplementary Figure S15B) revealed that no T22d35 concentration significantly (*p* < 0.05) reduced collagen deposition in comparison to the +MMC+TGF*β*1 group. Only the 50 nM T22d35 in +MMC+TGF*β*1 significantly (*p* < 0.05) increased DNA concentration, in comparison to +MMC+TGF*β*1 group, at day 10 (Supplementary Figure S16A). Only the 25 nM T22d35 in +MMC+TGF*β*1 significantly (*p* < 0.05) increased metabolic activity, in comparison to +MMC+TGF*β*1 group, at day 4 (Supplementary Figure S16B).

**FIGURE 9:**
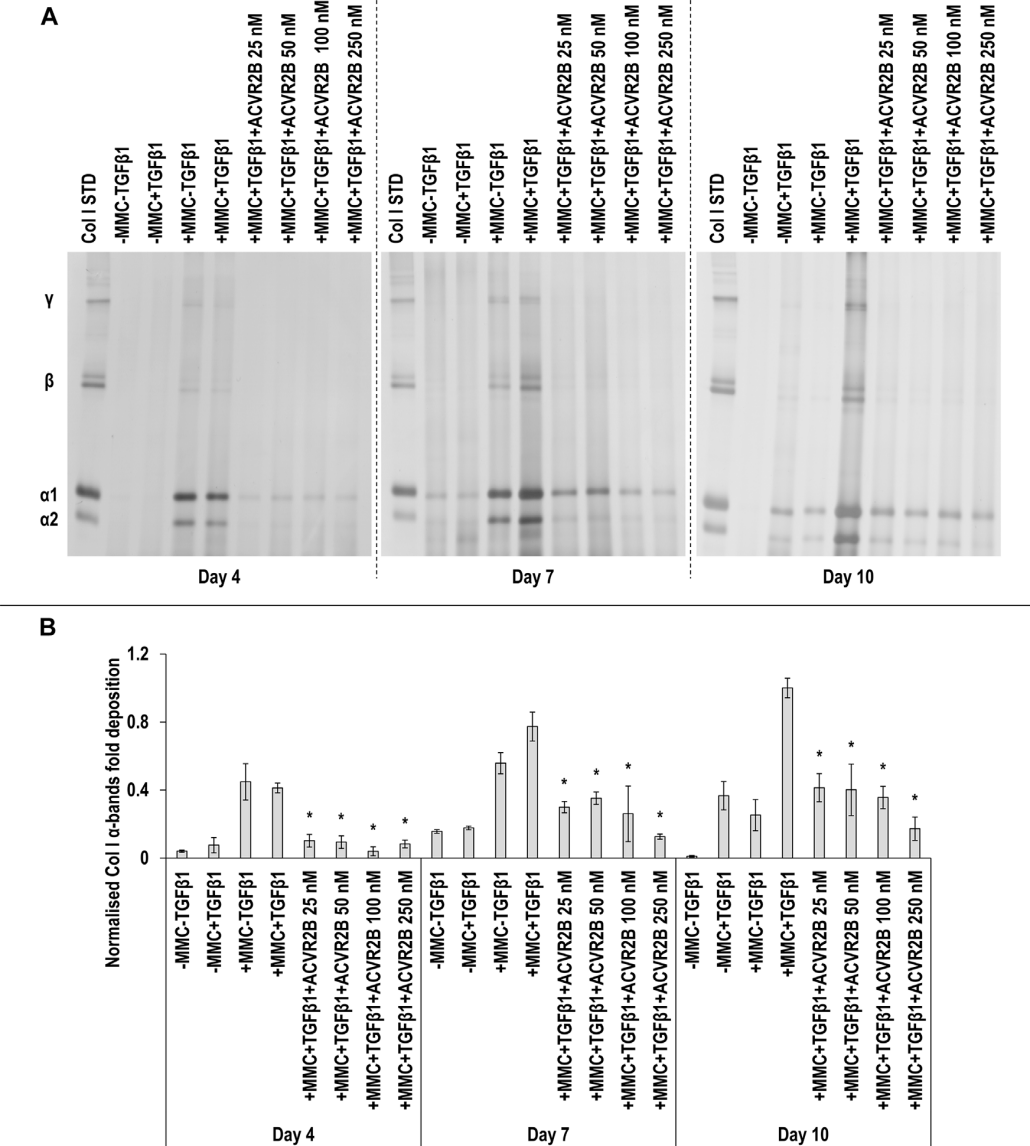
ACVR2B reduces collagen deposition. All ACVR2B concentrations in +MMC+TGFβ1 at all time points significantly reduced collagen deposition, in comparison to +MMC+TGFβ1 group, as judged by SDS-PAGE **(A)** and densitometry analysis of α(I)1 and α(I)2 bands **(B)**. Col I STD: 0.1 mg/ml. One-way ANOVA and Tukey’s post-hoc comparison tests or Kruskal Wallis and Mann Whitney post-hoc analyses were conducted, as appropriate. *: *p* < 0.05 indicates a statistically significant difference when compared to the +MMC+TGFβ1 group of the respective time point. *n* = 3.

SDS-PAGE (Figure 9A) and densitometry analysis of α(I)1 and α(I)2 (Figure 9B), *β*11(I), *β*12(I) (Supplementary Figure S17A) and γ(I) (Supplementary Figure S17B) bands revealed that all ACVR2B concentrations in +MMC+TGF*β*1 at all time points significantly (*p* < 0.05) reduced the deposition of α(I)1 and α(I)2, *β*11(I), *β*12(I) and γ(I) components, in comparison to the +MMC+TGF*β*1 group. Only the 25 and 50 nM ACVR2B concentrations in +MMC+TGF*β*1 significantly (*p* < 0.05) increased DNA concentration, in comparison to +MMC+TGF*β*1 group, at day 7 (Supplementary Figure S18A). The 25, 50 and 100 nM ACVR2B concentrations in +MMC+TGF*β*1 significantly (*p* < 0.05) decreased metabolic activity, in comparison to +MMC+TGF*β*1 group, at day 7 and the 250 nM concentration at day 10 (Supplementary Figure S18B).

SDS-PAGE (Supplementary Figure S19A) and densitometry of α(I)1 and α(I)2 bands (Supplementary Figure S19B) revealed that all concentrations of BAPN in +MMC+TGF*β*1 at day 4 and the 0.1, 0.25 and the 0.5 mM concentrations of BAPN in +MMC+TGF*β*1 at day 10 significantly (*p* < 0.05) increased α(I)1 and α(I)2 deposition in comparison to the +MMC+TGF*β*1 group at the respective time points. No significant (*p* < 0.05) differences in the deposition of *β*11(I), *β*12(I) (Supplementary Figure S20A) and γ(I) components (Supplementary Figure S20B) were observed. The 1 mM concentration of the BAPN in +MMC+TGF*β*1 significantly reduced DNA concentration at day 7 and day 10 in comparison to the +MMC+TGF*β*1 group (Supplementary Figure S21A). The 0.5 mM and the 1 mM BAPN concentration in +MMC+TGF*β*1 resulted in significantly increased metabolic activity at day 10 in comparison to the +MMC+TGF*β*1 group (Supplementary Figure S21B).

Qualitative cell viability assessment revealed that TAC did not have a negative affect; TSA, BAPN and T122bt had a negative effect at high concentrations (the latter, only at the latest time point); and RLX-2, Pirf, T22d35 and ACV2R had a negative effect mostly at later time points, regardless of concentration (Supplementary Figures S22–S29).

## Discussion

Fibrosis is not just the outcome of devastating skin diseases, but it can also be the result of abnormal skin wound healing and scarring. One of the fundamental roadblocks in the use of traditional *in vitro* models for screening anti-fibrotic molecules is that they do not recapitulate the excessive and altered ECM characteristic of human fibrotic diseases (Chen et al., 2009). To address this deficiency, we ventured to assess whether MMC (that dramatically enhances and accelerates ECM deposition) coupled with TGF*β*1 (that induces myofibroblast trans-differentiation) can generate an efficient skin fibrosis model.

Starting with the establishment of the fibrotic model, TGF*β*1 is a key player in many fibrotic conditions (Lodyga and Hinz, 2020), with its signalling pathway involved in transformation of fibroblasts into myofibroblasts (Sarrazy et al., 2011; Lichtman et al., 2016). MMC significantly increased collagen deposition and *a*SMA expression in TGF*β*1 supplemented cultures and resulted in the formation of an ECM-rich substrate, magnifying the profibrotic effect of TGF*β*1, a prerequisite for the development of a scarring model.

Although a quantitative analysis of cell and ECM orientation was not performed in this study, it was still possible to observe that treatment with macromolecular crowding and TGF*β*1 resulted in an alteration of the deposited ECM. Deposition of densely packed granular collagen type I (resembling the formation of scar tissue (Pakshir and Hinz, 2018)) was observable. On the other hand, in the absence of macromolecular crowding treatment, it was observed the normal meshwork architecture of healthy skin ECM (Goto et al., 2020). The formation of αSMA stress fibres alongside the deposition axis of collagen type I also seems to confirm this.

In accordance with previous publications, several other organ-specific fibrotic models induced by MMC supplementation have been developed (Chen et al., 2009; Graupp et al., 2015; Graupp et al., 2018; Fan et al., 2019; Good et al., 2019; Fan et al., 2020; Rønnow et al., 2020; De Pieri et al., 2021). It is worth noting that cocktails of pro-inflammatory cytokines have been used for more accurate recapitulation of fibrosis *in vitro* (Chawla and Ghosh, 2018) and such approach should be studied further in the future in combination with MMC.

After establishment of the *in vitro* model of skin fibrosis, several anti-fibrotic molecules with different mechanisms of action were tested to assess their capacity to decrease collagen synthesis and/or deposition. We also assessed their effect on basic cellular functions (cell metabolic activity, viability, DNA concentration) to assess how selective their mode of action is. Tested drug concentrations were based on previously published data that had been shown to have a therapeutic effect *in vitro*. This explains the different orders of magnitude in concentrations used for different anti-fibrotic molecules, which allowed us to validate the model and select the best, as judged by maximum collagen reduction and adequate profile regarding basic cellular functions, anti-fibrotic molecules.

The first molecule tested was TAC, and in this study, only a very moderate effect in the reduction of the deposition of collagen type I was observed. This synthetic corticosteroid, used to reduce inflammation, is currently one of the most used molecules for the treatment of hypertrophic scars and keloids in humans and arguably the “gold standard” treatment (Ogawa, 2010; Berman et al., 2017; Hietanen et al., 2019). Despite its wide usage, TAC presents only moderate response rates, while presenting relatively high recurrence rates (Ogawa, 2010; Hietanen et al., 2019) and considerable side effects on skin (Sadeghinia and Sadeghinia, 2012). We have recently demonstrated in a randomised controlled trial that keloids that respond to either TAC or 5-FU show a reduction in myofibroblasts (Hietanen et al., 2020). This is a secondary effect as neither TAC nor 5-FU have a direct influence on myofibroblast transformation. Our results support clinical data and illustrate that more effective and safer therapies than TAC should be developed for keloids and hypertrophic scars.

Another molecule tested was TSA and we observed an overall reduction in collagen type I deposition and cell proliferation. This is not surprising considering that it is an inhibitor of class I and II mammalian histone deacetylases (HDACs), which alter gene expression by altering the access of transcription factors to DNA. TSA has been shown to reduce *α*SMA expression and collagen type I deposition; inhibit cellular proliferation in fibroblasts; and promote apoptosis in certain experimental skin fibrosis models (Rombouts et al., 2002; Huber et al., 2007; Diao et al., 2011). HDACs, in general, have been implicated in TGF*β*1-induced epithelial-mesenchymal transition (EMT) (Jones et al., 2019; Qiao et al., 2020). HDAC suppress epithelial-specific genes and mediate TGF*β*1-induced mesenchymal enhancer reprogramming, which results in EMT of the cells (Qiao et al., 2020). As myofibroblast transformation is a central feature in fibrosis and our results demonstrate that TSA can inhibit it, this makes it a viable therapeutic candidate.

After treatment with RLX-2, a decrease in collagen type I deposition was observed, with no significant negative effects on basic cellular functions. To substantiate this, one should consider that recombinant human relaxin-2 (RLX-2), a naturally occurring peptide, binds to its receptor, RLX family peptide receptor 1. This has been shown to suppress not only Smad 2/3 and consequently, TGF*β*1 signalling pathways in cardiac fibroblasts, but also angiotensin II type 2 receptors and interleukin-1*β* and the potential interaction of these signalling pathways with the TGF*β* axis (Yuan et al., 2017; Wu et al., 2018), as well as with the inflammasome (Pinar et al., 2020). In addition to those, RLX-2 can increase the expression and activity of matrix metalloproteinases that facilitate ECM degradation (Li et al., 2021). Its application in other diseases, mainly fibrotic, has been postulated, although the clinical trials have been inconclusive on its therapeutic efficacy (Samuel et al., 2017; Yuan et al., 2017; Blessing et al., 2019; Hinz and Lagares, 2020).

When testing Pirf, our study showed decreased collagen deposition and an absence of significant adverse effects on basic cellular functions at the lowest drug concentrations tested. However, when compared to the other molecules tested, very high relative concentrations of the molecule were required to obtain anti-fibrotic activity. This follows the reported literature, as Pirf is a small molecule inhibitor and a Food and Drug Administration approved drug for idiopathic lung fibrosis, but large doses are required (Noble et al., 2011; Lancaster et al., 2017). It has been investigated as a treatment for several other fibrotic diseases, such as liver (Seniutkin et al., 2018), kidney (Salah et al., 2019), intestinal (Sun et al., 2018) and skin (Hall et al., 2018). It functions by inhibiting TGF*β*1 (and its production) and platelet-derived growth factor-activated signalling pathways (Lv et al., 2020) in conjunction with anti-inflammatory activity (Mora et al., 2015; Hall et al., 2018). Due to the high doses required, Pirf is associated with side-effects and organ toxicity (Anderson et al., 2016; Lancaster et al., 2017), which require rigorous supervision and limit its therapeutic potential. One option could be to combine a low dose of pirfenidone with another anti-fibrotic molecule. By that approach, side-effects could hopefully be avoided, and the therapeutic effect enhanced.

Following treatment with both TGFβ traps, no significant effects on collagen deposition were observed for T22d35, while T122bt resulted in a significant decrease of collagen deposition, at a concentration several orders of magnitude lower than for some of the other molecules. Neither of the TGF*β* trap molecules elicited a negative response regarding basic cellular functions. This is expected, as given the importance of the TGF*β* signalling pathway in the transition to a fibrotic phenotype, recombinant TGF*β* traps, i.e., soluble ligand binding parts of the TGF*β* receptors can act as inhibitors of this pathway. They can bind different isoforms of TGF*β,* namely *-β*1 and -*β*3 for T22d35 and all three isoforms for T122bt. Both molecules can inhibit different isoforms of TGF*β* at almost picomolar concentration, which is to our knowledge, the lowest concentration that has been attained against TGF*β* (Zwaagstra et al., 2012; O'Connor-Mccourt et al., 2013). Concerning the difference between the two traps and TGF*β* isoforms, TGF*β*2 augments the profibrotic functions of TGF*β*1 (Jagadeesan and Bayat, 2007), whereas TGF*β*3 has been hypothesised to possess anti-fibrotic functions (Occleston et al., 2008; Lichtman et al., 2016). Thus, the T122b trap has a preferential TGF*β* inhibitory profile than T22d35. Furthermore, both tested molecules have demonstrated substantial anti-tumour effect in TGF*β*-driven cancer models *in vivo* (Zwaagstra et al., 2012; O'Connor-Mccourt et al., 2013). As TGF*β*1 was the only growth factor supplied in the media, our results indicate that these traps are very potent and specific in their inhibitory activity.

We also tested an activin IIB receptor antagonist, a soluble ligand binding domain part of the receptor. Despite a substantial decrease in collagen type I deposition, a decrease in basic cellular functions was observed for ACVR2B. This can be explained as this antagonist blocks signalling of activins A and B, myostatin (MSTN/GDF-8), its close homolog, growth differentiation factor-11 (GDF-11) and bone morphogenetic protein-10 (Lautaoja et al., 2019; Magga et al., 2019; Szabó et al., 2020). Both activins are involved in Smad 2/3 signalling and consequently play a role in fibrosis (Werner and Alzheimer, 2006; Canady et al., 2013; Walton et al., 2017; Itoh et al., 2018). The expression of activins is induced during wound repair and activin A leads to accelerated wound healing (Cangkrama et al., 2020). However, its most striking effect is on the granulation tissue formation as it induces excessive scar formation (Cangkrama et al., 2020; Wietecha et al., 2020). This is attributed to the expression of its target genes in fibroblasts, which include both ACTA2 and COL1A1 (*α*SMA and collagen type I) (Cangkrama et al., 2020). Both MMC and TGF*β1*, in turn, induce the expression of activins and myostatin in fibrotic disorders (Erämaa and Ritvos, 1996; Cianciolo et al., 2020; Winter et al., 2020). As activins are produced in mature, active form, they can induce cell signalling immediately (Cangkrama et al., 2020). Furthermore, whereas TGF*β*1 signalling becomes refractory for extended periods of time due to receptor internalisation and degradation, the activin signalling remains active all the time due to constant receptor renewal at cell surface (Miller et al., 2019). Thus, our model could involve activin activity although activins were not supplemented to the culture media.

Treatment with BAPN resulted in decreased cell proliferation, although no significant decrease in collagen type I deposition was observed, which is in accordance with what was reported in previous studies. Given its action as a lysyl oxidase (LOX) inhibitor, which inhibits collagen crosslinking (Redden and Doolin, 2003), its use has been previously suggested as an anti-fibrotic (Trackman, 2016). However, several safety concerns related to osteolathyrism, a collagen cross-linking deficiency (Wilmarth and Froines, 1992; Rosenthal, 2003) have been reported following its use. The target enzyme, LOX, has a wide variety of biological effects beyond collagen cross-linking, which also influences matrix stiffness and cell proliferation and migration (Saatci et al., 2020; Freeberg et al., 2021; Kozma et al., 2021; Sflomos et al., 2021). This is an indicator of its unsuitability as an anti-fibrotic molecule and potentially the reason for the molecule’s failures in clinical trials.

## Conclusion

The low extracellular matrix content in the traditional *in vitro* fibrosis models results in poor imitation of the tissue pathology and to scattered predictive capacity. This study advocates the use of macromolecular crowding (to enhance and accelerate extracellular matrix deposition) and TGF*β* 1 (to induce dermal fibroblast trans-differentiation to myofibroblast) in the development of skin fibrosis specific *in vitro* models. We further identified trichostatin A, serelaxin, pirfenidone and soluble TGF*β* trap as potent anti-fibrotic therapies.

## Data Availability

The raw data supporting the conclusions of this article will be made available by the authors, without undue reservation.

## Abbreviations

*α*SMA, *α* smooth muscle actin; ACVR2B, Activin IIB receptor inhibitor; ANOVA, Analysis of variance; BAPN, *β*-aminopropionitrile; DF, Dermal fibroblasts; DAPI, 4,6-diamidino-2-phenylindole; DMEM, Dulbecco’s Modified Eagle’s Medium; DSC, Differential scanning calorimetry; DxS, Dextran sulphate; ECM, Extracellular matrix; EDTA, Ethylenediaminetetraacetic acid; EMT, Epithelial–mesenchymal transition; FBS, Foetal bovine serum; GDF-8/11, Growth differentiation factor-8/11; HDACs, class I and II mammalian histone deacetylases; LOX, Lysyl oxidase; MMC, Macromolecular crowding; MSTN/GDF-8, Myostatin; PBS, Phosphate buffered saline; Pirf, Pirfenidone; RLX-2, Recombinant human relaxin-2 (Serelaxin); SDS-PAGE, Sodium dodecyl sulphate-polyacrylamide gel electrophoresis; Smad 2/3, Small mothers against decapentaplegic protein 2/3; T22d35, T122bt, TGF*β* type II receptor-based trap; TAC, Triamcinolone acetonide; TBS, Tris-buffered saline; TGF*β*1, Transforming growth factor *β*1; TSA, Trichostatin A.

## References

[B1] Anderson A. Shifren A. Nathan S. D. (2016). A Safety Evaluation of Pirfenidone for the Treatment of Idiopathic Pulmonary Fibrosis. Expert Opin. Drug Saf. 15, 975–982. 10.1080/14740338.2016.1187129 27177012

[B2] Berman B. Maderal A. Raphael B. (2017). Keloids and Hypertrophic Scars: Pathophysiology, Classification, and Treatment. Dermatol. Surg. 43 Suppl 1 (Suppl. 1), S3–S18. 10.1097/DSS.0000000000000819 27347634

[B3] Blessing W. A. Okajima S. M. Cubria M. B. Villa-Camacho J. C. Perez-Viloria M. Williamson P. M. (2019). Intraarticular Injection of Relaxin-2 Alleviates Shoulder Arthrofibrosis. Proc. Natl. Acad. Sci. USA 116, 12183–12192. 10.1073/pnas.1900355116 31160441 PMC6589647

[B4] Canady J. Karrer S. Fleck M. Bosserhoff A. K. (2013). Fibrosing Connective Tissue Disorders of the Skin: Molecular Similarities and Distinctions. J. Dermatol. Sci. 70, 151–158. 10.1016/j.jdermsci.2013.03.005 23631956

[B5] Cancela L. Rebut-Bonneton C. (1987). Regulation of 24-hydroxylase Activity in Mouse Skin Fibroblasts by Cholecalciferol Derivatives, Triamcinolone Acetonide and a Calcium Modulating Agent, Nicardipine. J. Steroid Biochem. 28, 479–484. 10.1016/0022-4731(87)90505-x 3682815

[B6] Cangkrama M. Wietecha M. Werner S. (2020). Wound Repair, Scar Formation, and Cancer: Converging on Activin. Trends Mol. Med. 26, 1107–1117. 10.1016/j.molmed.2020.07.009 32878730

[B7] Capella-Monsonís H. Coentro J. Q. Graceffa V. Wu Z. Zeugolis D. I. (2018). An Experimental Toolbox for Characterization of Mammalian Collagen Type I in Biological Specimens. Nat. Protoc. 13, 507–529. 10.1038/nprot.2017.117 29446773

[B8] Carroll L. A. Hanasono M. M. Mikulec A. A. Kita M. Koch R. J. (2002). Triamcinolone Stimulates bFGF Production and Inhibits TGF-β1 Production by Human Dermal Fibroblasts. Dermatol. Surg. 28, 704–709. 10.1046/j.1524-4725.2002.02012.x 12174062

[B9] Chawla S. Ghosh S. (2018). Regulation of Fibrotic Changes by the Synergistic Effects of Cytokines, Dimensionality and Matrix: Towards the Development of an *In Vitro* Human Dermal Hypertrophic Scar Model. Acta Biomater. 69, 131–145. 10.1016/j.actbio.2018.01.002 29330036

[B10] Chen C. Peng Y. Wang Z. Fish P. Kaar J. Koepsel R. (2009). The Scar-In-A-Jar: Studying Potential Antifibrotic Compounds from the Epigenetic to Extracellular Level in a Single Well. Br. J. Pharmacol. 158, 1196–1209. 10.1111/j.1476-5381.2009.00387.x 19785660 PMC2782330

[B11] Cianciolo G. La Manna G. Capelli I. Gasperoni L. Galassi A. Ciceri P. (2020). The Role of Activin: the Other Side of Chronic Kidney Disease-mineral Bone Disorder? Nephrol. Dial. Transpl. 36, 966–974. 10.1093/ndt/gfaa203 32940690

[B12] Coentro J. Q. Pugliese E. Hanley G. Raghunath M. Zeugolis D. I. (2018). Current and Upcoming Therapies to Modulate Skin Scarring and Fibrosis. Adv. Drug Deliv. Rev. 146, 37–59. 10.1016/j.addr.2018.08.009 30172924

[B13] De Pieri A. Korman B. D. Jüngel A. Wuertz-Kozak K. (2021). Engineering Advanced *In Vitro* Models of Systemic Sclerosis for Drug Discovery and Development. Adv. Biol. 5, e2000168. 10.1002/adbi.202000168 PMC8717409 33852183

[B14] Desallais L. Avouac J. Fréchet M. Elhai M. Ratsimandresy R. Montes M. (2014). Targeting IL-6 by Both Passive or Active Immunization Strategies Prevents Bleomycin-Induced Skin Fibrosis. Arthritis Res. Ther. 16, R157. 10.1186/ar4672 25059342 PMC4220089

[B15] Diao J.-S. Xia W.-S. Yi C.-G. Wang Y.-M. Li B. Xia W. (2011). Trichostatin A Inhibits Collagen Synthesis and Induces Apoptosis in Keloid Fibroblasts. Arch. Dermatol. Res. 303, 573–580. 10.1007/s00403-011-1140-1 21400246

[B16] Erämaa M. Ritvos O. (1996). Endocrinology and Paracrinology. Mol. Hum. Reprod. 2, 815–822. 10.1093/molehr/2.11.815 9237220

[B17] Fan C. Lim L. K. P. Loh S. Q. Ying Lim K. Y. Upton Z. Leavesley D. (2019). Application of "Macromolecular Crowding" *In Vitro* to Investigate the Naphthoquinones Shikonin, Naphthazarin and Related Analogues for the Treatment of Dermal Scars. Chem. Biol. Interact. 310, 108747. 10.1016/j.cbi.2019.108747 31301289

[B18] Fan C. Lim L. K. P. Wu Z. Sharma B. Gan S. Q. Liang K. (2020). *In Vitro* Model of Human Cutaneous Hypertrophic Scarring Using Macromolecular Crowding. J. Vis. Exp. (159), e61037. 10.3791/61037 32420993

[B19] Freeberg M. A. T. Perelas A. Rebman J. K. Phipps R. P. Thatcher T. H. Sime P. J. (2021). Mechanical Feed-Forward Loops Contribute to Idiopathic Pulmonary Fibrosis. Am. J. Pathol. 191, 18–25. 10.1016/j.ajpath.2020.09.008 33031756 PMC7768346

[B20] Ghosh A. K. Mori Y. Dowling E. Varga J. (2007). Trichostatin A Blocks TGF-β-Induced Collagen Gene Expression in Skin Fibroblasts: Involvement of Sp1. Biochem. Biophys. Res. Commun. 354, 420–426. 10.1016/j.bbrc.2006.12.204 17234156

[B21] Good R. B. Eley J. D. Gower E. Butt G. Blanchard A. D. Fisher A. J. (2019). A High Content, Phenotypic 'scar-In-A-Jar' Assay for Rapid Quantification of Collagen Fibrillogenesis Using Disease-Derived Pulmonary Fibroblasts. BMC Biomed. Eng. 1, 14. 10.1186/s42490-019-0014-z 32903343 PMC7422573

[B22] Goto H. Tada A. Ibe A. Kitajima Y. (2020). Basket‐weave Structure in the Stratum Corneum Is an Important Factor for Maintaining the Physiological Properties of Human Skin as Studied Using Reconstructed Human Epidermis and Tape Stripping of Human Cheek Skin. Br. J. Dermatol. 182, 364–372. 10.1111/bjd.18123 31077338

[B23] Graupp M. Gruber H.-J. Weiss G. Kiesler K. Bachna-Rotter S. Friedrich G. (2015). Establishing Principles of Macromolecular Crowding for *In Vitro* Fibrosis Research of the Vocal Fold Lamina Propria. Laryngoscope 125, E203–E209. 10.1002/lary.25103 25545625

[B24] Graupp M. Rinner B. Frisch M. T. Weiss G. Fuchs J. Sundl M. (2018). Towards an *In Vitro* Fibrogenesis Model of Human Vocal Fold Scarring. Eur. Arch. Otorhinolaryngol. 275, 1211–1218. 10.1007/s00405-018-4922-7 29520499 PMC5893733

[B25] Griffin M. F. desJardins-Park H. E. Mascharak S. Borrelli M. R. Longaker M. T. (2020). Understanding the Impact of Fibroblast Heterogeneity on Skin Fibrosis. Dis. Model. Mech. 13, dmm044164. 10.1242/dmm.044164 32541065 PMC7328159

[B26] Hall C. L. Wells A. R. Leung K. P. (2018). Pirfenidone Reduces Profibrotic Responses in Human Dermal Myofibroblasts, *In Vitro* . Lab. Invest. 98, 640–655. 10.1038/s41374-017-0014-3 29497173

[B27] Henderson N. C. Rieder F. Wynn T. A. (2020). Fibrosis: From Mechanisms to Medicines. Nature 587, 555–566. 10.1038/s41586-020-2938-9 33239795 PMC8034822

[B28] Hietanen K. Järvinen T. Huhtala H. Tolonen T. Kuokkanen H. Kaartinen I. (2019). Treatment of Keloid Scars with Intralesional Triamcinolone and 5-fluorouracil Injections - a Randomized Controlled Trial. J. Plast. Reconstr. Aesthet. Surg. 72, 4–11. 10.1016/j.bjps.2018.05.052 30448246

[B29] Hietanen K. E. Järvinen T. A. H. Huhtala H. Tolonen T. T. Kaartinen I. S. (2020). Histopathology and Immunohistochemical Analysis of 5‐fluorouracil and Triamcinolone Treated Keloids in Double‐blinded Randomized Controlled Trial. Wound Rep. Reg. 28, 385–399. 10.1111/wrr.12803 32112591

[B30] Hinz B. Lagares D. (2020). Evasion of Apoptosis by Myofibroblasts: A Hallmark of Fibrotic Diseases. Nat. Rev. Rheumatol. 16, 11–31. 10.1038/s41584-019-0324-5 31792399 PMC7913072

[B31] Hinz B. Phan S. H. Thannickal V. J. Prunotto M. Desmoulière A. Varga J. (2012). Recent Developments in Myofibroblast Biology. Am. J. Pathol. 180, 1340–1355. 10.1016/j.ajpath.2012.02.004 22387320 PMC3640252

[B32] Huber L. C. Distler J. H. W. Moritz F. Hemmatazad H. Hauser T. Michel B. A. (2007). Trichostatin A Prevents the Accumulation of Extracellular Matrix in a Mouse Model of Bleomycin-Induced Skin Fibrosis. Arthritis Rheum. 56, 2755–2764. 10.1002/art.22759 17665426

[B33] Itoh Y. Saitoh M. Miyazawa K. (2018). Smad3-STAT3 Crosstalk in Pathophysiological Contexts. Acta Biochim. Biophys. Sin. 50, 82–90. 10.1093/abbs/gmx118 29140406

[B34] Jagadeesan J. Bayat A. (2007). Transforming Growth Factor Beta (TGFβ) and Keloid Disease. Int. J. Surg. 5, 278–285. 10.1016/j.ijsu.2006.04.007 17660136

[B35] Jarvinen T. A. H. Ruoslahti E. (2010). Target-seeking Antifibrotic Compound Enhances Wound Healing and Suppresses Scar Formation in Mice. Proc. Natl. Acad. Sci. 107, 21671–21676. 10.1073/pnas.1016233107 21106754 PMC3003105

[B36] Jones D. L. Haak A. J. Caporarello N. Choi K. M. Ye Z. Yan H. (2019). Tgfβ-induced Fibroblast Activation Requires Persistent and Targeted HDAC-Mediated Gene Repression. J. Cel Sci. 132, jcs233486. 10.1242/jcs.233486 PMC6826010 31527052

[B37] Kozma K. J. Done S. J. Egan S. E. (2021). The Tumor Cell-Derived Matrix of Lobular Breast Cancer: A New Vulnerability. EMBO Mol. Med. 13, e13807. 10.15252/emmm.202013807 33616312 PMC7933957

[B38] Kumar P. Satyam A. Fan X. Rochev Y. Rodriguez B. J. Gorelov A. (2015). Accelerated Development of Supramolecular Corneal Stromal-like Assemblies from Corneal Fibroblasts in the Presence of Macromolecular Crowders. Tissue Eng. C: Methods 21, 660–670. 10.1089/ten.tec.2014.0387 25535812

[B39] Lancaster L. H. de Andrade J. A. Zibrak J. D. Padilla M. L. Albera C. Nathan S. D. (2017). Pirfenidone Safety and Adverse Event Management in Idiopathic Pulmonary Fibrosis. Eur. Respir. Rev. 26, 170057. 10.1183/16000617.0057-2017 29212837 PMC9488585

[B40] Lautaoja J. H. Lalowski M. Nissinen T. A. Hentilä J. Shi Y. Ritvos O. (2019). Muscle and Serum Metabolomes Are Dysregulated in colon-26 Tumor-Bearing Mice Despite Amelioration of Cachexia with Activin Receptor Type 2B Ligand Blockade. Am. J. Physiol. Endocrinol. Metab. 316, E852–E865. 10.1152/ajpendo.00526.2018 30860875

[B41] Li Y. Shen M. Ferens D. Broughton B. R. S. Murthi P. Saini S. (2021). Combining Mesenchymal Stem Cells with Serelaxin Provides Enhanced Renoprotection against 1K/DOCA/salt‐induced Hypertension. Br. J. Pharmacol. 178, 1164–1181. 10.1111/bph.15361 33450051

[B42] Lichtman M. K. Otero-Vinas M. Falanga V. (2016). Transforming Growth Factor Beta (TGF-β) Isoforms in Wound Healing and Fibrosis. Wound Rep. Reg. 24, 215–222. 10.1111/wrr.12398 26704519

[B43] Lodyga M. Hinz B. (2020). TGF-β1 - A Truly Transforming Growth Factor in Fibrosis and Immunity. Semin. Cel Dev. Biol. 101, 123–139. 10.1016/j.semcdb.2019.12.010 31879265

[B44] Logeart-Avramoglou D. Huynh R. Chaubet F. Sedel L. Meunier A. (2002). Interaction of Specifically Chemically Modified Dextrans with Transforming Growth Factor β1: Potentiation of its Biological Activity. Biochem. Pharmacol. 63, 129–137. 10.1016/s0006-2952(01)00834-6 11841786

[B45] Lv Q. Wang J. Xu C. Huang X. Ruan Z. Dai Y. (2020). Pirfenidone Alleviates Pulmonary Fibrosis *In Vitro* and *In Vivo* through Regulating Wnt/GSK-3β/β-Catenin and TGF-β1/Smad2/3 Signaling Pathways. Mol. Med. 26, 49. 10.1186/s10020-020-00173-3 32448163 PMC7245944

[B46] Magga J. Vainio L. Kilpiö T. Hulmi J. J. Taponen S. Lin R. (2019). Systemic Blockade of ACVR2B Ligands Protects Myocardium from Acute Ischemia-Reperfusion Injury. Mol. Ther. 27, 600–610. 10.1016/j.ymthe.2019.01.013 30765322 PMC6404100

[B47] Maire M. Logeart-Avramoglou D. Degat M.-C. Chaubet F. (2005). Retention of Transforming Growth Factor β1 Using Functionalized Dextran-Based Hydrogels. Biomaterials 26, 1771–1780. 10.1016/j.biomaterials.2004.06.003 15576151

[B48] Miller D. S. J. Schmierer B. Hill C. S. (2019). TGF-β Family Ligands Exhibit Distinct Signalling Dynamics that Are Driven by Receptor Localisation. J. Cel Sci. 132, jcs234039. 10.1242/jcs.234039 PMC6679586 31217285

[B49] Mora D. A. L.-d. l. Sanchez-Roque C. Montoya-Buelna M. Sanchez-Enriquez S. Lucano-Landeros S. Macias-Barragan J. (2015). Role and New Insights of Pirfenidone in Fibrotic Diseases. Int. J. Med. Sci. 12, 840–847. 10.7150/ijms.11579 26640402 PMC4643073

[B50] Noble P. W. Albera C. Bradford W. Z. Costabel U. Glassberg M. K. Kardatzke D. (2011). Pirfenidone in Patients with Idiopathic Pulmonary Fibrosis (CAPACITY): Two Randomised Trials. Lancet 377, 1760–1769. 10.1016/s0140-6736(11)60405-4 21571362

[B51] Occleston N. L. Laverty H. G. O'Kane S. Ferguson M. W. J. (2008). Prevention and Reduction of Scarring in the Skin by Transforming Growth Factor Beta 3 (TGFβ3): from Laboratory Discovery to Clinical Pharmaceutical. J. Biomater. Sci. Polym. Ed. 19, 1047–1063. 10.1163/156856208784909345 18644230

[B52] O'Connor-Mccourt M. Sulea T. Zwaagstra J. Baardsnes J. (2013). Antagonists of Ligands and Uses Thereof. US8574548B2. Available at: https://patentscope.wipo.int/search/en/detail.jsf?docId=WO2008113185 .

[B53] Ogawa R. (2010). The Most Current Algorithms for the Treatment and Prevention of Hypertrophic Scars and Keloids. Plast. Reconstr. Surg. 125, 557–568. 10.1097/prs.0b013e3181c82dd5 20124841

[B54] Padmanabhan J. Maan Z. N. Kwon S. H. Kosaraju R. Bonham C. A. Gurtner G. C. (2019). *In Vivo* models for the Study of Fibrosis. Adv. Wound Care 8, 645–654. 10.1089/wound.2018.0909 PMC6904938 31827979

[B55] Pakshir P. Hinz B. (2018). The Big Five in Fibrosis: Macrophages, Myofibroblasts, Matrix, Mechanics, and Miscommunication. Matrix Biol. 68-69, 81–93. 10.1016/j.matbio.2018.01.019 29408013

[B56] Pakshir P. Noskovicova N. Lodyga M. Son D. O. Schuster R. Goodwin A. (2020). The Myofibroblast at a Glance. J. Cel Sci. 133, jcs227900. 10.1242/jcs.227900 32651236

[B57] Péterszegi G. Andrès E. Molinari J. Ravelojaona V. Robert L. (2008). Effect of Cellular Aging on Collagen Biosynthesis. Arch. Gerontol. Geriatr. 47, 356–367. 10.1016/j.archger.2007.08.019 17961760

[B58] Pinar A. A. Yuferov A. Gaspari T. A. Samuel C. S. (2020). Relaxin Can Mediate its Anti-fibrotic Effects by Targeting the Myofibroblast NLRP3 Inflammasome at the Level of Caspase-1. Front. Pharmacol. 11, 1201. 10.3389/fphar.2020.01201 32848798 PMC7417934

[B59] Qiao Y. Wang Z. Tan F. Chen J. Lin J. Yang J. (2020). Enhancer Reprogramming within Pre-existing Topologically Associated Domains Promotes TGF-β-Induced EMT and Cancer Metastasis. Mol. Ther. 28, 2083–2095. 10.1016/j.ymthe.2020.05.026 32526202 PMC7474343

[B60] Raghunath M. Zeugolis D. I. (2021). Transforming Eukaryotic Cell Culture with Macromolecular Crowding. Trends Biochem. Sci. 46, 805–811. 10.1016/j.tibs.2021.04.006 33994289

[B61] Rashid R. Lim N. S. J. Chee S. M. L. Png S. N. Wohland T. Raghunath M. (2014). Novel Use for Polyvinylpyrrolidone as a Macromolecular crowder for Enhanced Extracellular Matrix Deposition and Cell Proliferation. Tissue Eng. Part C: Methods 20, 994–1002. 10.1089/ten.tec.2013.0733 24665935 PMC4241873

[B62] Redden R. A. Doolin E. J. (2003). Collagen Crosslinking and Cell Density Have Distinct Effects on Fibroblast-Mediated Contraction of Collagen Gels. Skin Res. Technol. 9, 290–293. 10.1034/j.1600-0846.2003.00023.x 12877693

[B63] Rombouts K. Niki T. Greenwel P. Vandermonde A. Wielant A. Hellemans K. (2002). Trichostatin A, a Histone Deacetylase Inhibitor, Suppresses Collagen Synthesis and Prevents TGF-β1-Induced Fibrogenesis in Skin Fibroblasts. Exp. Cel Res. 278, 184–197. 10.1006/excr.2002.5577 12169274

[B64] Rønnow S. R. Dabbagh R. Q. Genovese F. Nanthakumar C. B. Barrett V. J. Good R. B. (2020). Prolonged Scar-In-A-Jar: An *In Vitro* Screening Tool for Anti-Fibrotic Therapies Using Biomarkers of Extracellular Matrix Synthesis. Respir. Res. 21, 108. 10.1186/s12931-020-01369-1 32381012 PMC7203825

[B65] Rosenthal G. (2003). Toxic Constituents and Their Related Metabolites, Plant Nonprotein Amino and Imino Acids: Biological, Biochemical, and Toxicological Properties. New York: Academic Press, 57–157.

[B66] Saatci O. Kaymak A. Raza U. Ersan P. G. Akbulut O. Banister C. E. (2020). Targeting Lysyl Oxidase (LOX) Overcomes Chemotherapy Resistance in Triple Negative Breast Cancer. Nat. Commun. 11, 2416. 10.1038/s41467-020-16199-4 32415208 PMC7229173

[B67] Sadeghinia A. Sadeghinia S. (2012). Comparison of the Efficacy of Intralesional Triamcinolone Acetonide and 5-fluorouracil Tattooing for the Treatment of Keloids. Dermatol. Surg. 38, 104–109. 10.1111/j.1524-4725.2011.02137.x 22093096

[B68] Saito M. Yamazaki M. Maeda T. Matsumura H. Setoguchi Y. Tsuboi R. (2012). Pirfenidone Suppresses Keloid Fibroblast-Embedded Collagen Gel Contraction. Arch. Dermatol. Res. 304, 217–222. 10.1007/s00403-011-1184-2 22033529

[B69] Salah M. M. Ashour A. A. Abdelghany T. M. Abdel-Aziz A.-A. H. Salama S. A. (2019). Pirfenidone Alleviates Concanavalin A-Induced Liver Fibrosis in Mice. Life Sci. 239, 116982. 10.1016/j.lfs.2019.116982 31639402

[B70] Samuel C. S. Sakai L. Y. Amento E. P. (2003). Relaxin Regulates Fibrillin 2, but Not Fibrillin 1, mRNA and Protein Expression by Human Dermal Fibroblasts and Murine Fetal Skin. Arch. Biochem. Biophys. 411, 47–55. 10.1016/s0003-9861(02)00710-5 12590922

[B71] Samuel C. S. Royce S. G. Hewitson T. D. Denton K. M. Cooney T. E. Bennett R. G. (2017). Anti‐fibrotic Actions of Relaxin. Br. J. Pharmacol. 174, 962–976. 10.1111/bph.13529 27250825 PMC5406285

[B72] Sarrazy V. Billet F. Micallef L. Coulomb B. Desmoulière A. (2011). Mechanisms of Pathological Scarring: Role of Myofibroblasts and Current Developments. Wound Repair Regen. 19 (Suppl. 1), s10–s15. 10.1111/j.1524-475x.2011.00708.x 21793960

[B73] Schulz J.-N. Plomann M. Sengle G. Gullberg D. Krieg T. Eckes B. (2018). New Developments on Skin Fibrosis - Essential Signals Emanating from the Extracellular Matrix for the Control of Myofibroblasts. Matrix Biol. 68-69, 522–532. 10.1016/j.matbio.2018.01.025 29408278

[B74] Seniutkin O. Furuya S. Luo Y.-S. Cichocki J. A. Fukushima H. Kato Y. (2018). Effects of Pirfenidone in Acute and Sub-chronic Liver Fibrosis, and an Initiation-Promotion Cancer Model in the Mouse. Toxicol. Appl. Pharmacol. 339, 1–9. 10.1016/j.taap.2017.11.024 29197520

[B75] Seo B. R. Chen X. Ling L. Song Y. H. Shimpi A. A. Choi S. (2020). Collagen Microarchitecture Mechanically Controls Myofibroblast Differentiation. Proc. Natl. Acad. Sci. USA 117, 11387–11398. 10.1073/pnas.1919394117 32385149 PMC7260976

[B76] Sflomos G. Battista L. Aouad P. De Martino F. Scabia V. Stravodimou A. (2021). Intraductal Xenografts Show Lobular Carcinoma Cells Rely on Their Own Extracellular Matrix and LOXL1. EMBO Mol. Med. 13, e13180. 10.15252/emmm.202013180 33616307 PMC7933935

[B77] Stebler S. Raghunath M. (2021). The : *In Vitro* Fibrosis Model for Anti-fibrotic Drug Testing. Methods Mol. Biol. 2299, 147–156. 10.1007/978-1-0716-1382-5_11 34028741

[B78] Sun Y.-W. Zhang Y.-Y. Ke X.-J. Wu X.-j. Chen Z.-F. Chi P. (2018). Pirfenidone Prevents Radiation-Induced Intestinal Fibrosis in Rats by Inhibiting Fibroblast Proliferation and Differentiation and Suppressing the TGF-β1/Smad/CTGF Signaling Pathway. Eur. J. Pharmacol. 822, 199–206. 10.1016/j.ejphar.2018.01.027 29374548

[B79] Sund B. Arrow A. K. (2000). New Developments in Wound Care. Clin. Rep. 45, 379.

[B80] Szabó Z. Vainio L. Lin R. Swan J. Hulmi J. J. Rahtu‐Korpela L. (2020). Systemic Blockade of ACVR2B Ligands Attenuates Muscle Wasting in Ischemic Heart Failure without Compromising Cardiac Function. FASEB j. 34, 9911–9924. 10.1096/fj.201903074rr 32427381

[B81] Trackman P. C. (2016). Lysyl Oxidase Isoforms and Potential Therapeutic Opportunities for Fibrosis and Cancer. Expert Opin. Ther. Targets 20, 935–945. 10.1517/14728222.2016.1151003 26848785 PMC4988797

[B82] Tsiapalis D. Zeugolis D. I. (2021). It Is Time to Crowd Your Cell Culture media - Physicochemical Considerations with Biological Consequences. Biomaterials 275, 120943. 10.1016/j.biomaterials.2021.120943 34139505

[B83] Unemori E. N. Amento E. P. (1990). Relaxin Modulates Synthesis and Secretion of Procollagenase and Collagen by Human Dermal Fibroblasts. J. Biol. Chem. 265, 10681–10685. 10.1016/s0021-9258(18)87000-4 2162358

[B84] Unemori E. N. Bauer E. A. Amento E. P. (1992). Relaxin Alone and in Conjunction with Interferon-γ Decreases Collagen Synthesis by Cultured Human Scleroderma Fibroblasts. J. Invest. Dermatol. 99, 337–342. 10.1111/1523-1747.ep12616665 1512471

[B85] Walton K. L. Johnson K. E. Harrison C. A. (2017). Targeting TGF-β Mediated SMAD Signaling for the Prevention of Fibrosis. Front. Pharmacol. 8, 461. 10.3389/fphar.2017.00461 28769795 PMC5509761

[B86] Walton K. W. (1952). The Biological Behaviour of a New Synthetic Anticoagulant (Dextran Sulphate) Possessing Heparin-like Properties. Br. J. Pharmacol. 7, 370–391. 10.1111/j.1476-5381.1952.tb00705.x PMC1509121 12978241

[B87] Wells A. R. Leung K. P. (2020). Pirfenidone Attenuates the Profibrotic Contractile Phenotype of Differentiated Human Dermal Myofibroblasts. Biochem. Biophys. Res. Commun. 521, 646–651. 10.1016/j.bbrc.2019.10.177 31679692

[B88] Werner S. Alzheimer C. (2006). Roles of Activin in Tissue Repair, Fibrosis, and Inflammatory Disease. Cytokine Growth Factor. Rev. 17, 157–171. 10.1016/j.cytogfr.2006.01.001 16481210

[B89] Wietecha M. S. Pensalfini M. Cangkrama M. Müller B. Jin J. Brinckmann J. (2020). Activin-mediated Alterations of the Fibroblast Transcriptome and Matrisome Control the Biomechanical Properties of Skin Wounds. Nat. Commun. 11, 2604. 10.1038/s41467-020-16409-z 32451392 PMC7248062

[B90] Wilmarth K. R. Froines J. R. (1992). *In Vitro* and *In Vivo* Inhibition of Lysyl Oxidase Byaminopropionitriles. J. Toxicol. Environ. Health 37, 411–423. 10.1080/15287399209531680 1359158

[B91] Winter A. Salamonsen L. A. Evans J. (2020). Modelling Fibroid Pathology: Development and Manipulation of a Myometrial Smooth Muscle Cell Macromolecular Crowding Model to Alter Extracellular Matrix Deposition. Mol. Hum. Reprod. 26, 498–509. 10.1093/molehr/gaaa036 32449756

[B92] Wu X.-p. Wang H.-j. Wang Y.-l. Shen H.-r. Tan Y.-z. (2018). Serelaxin Inhibits Differentiation and Fibrotic Behaviors of Cardiac Fibroblasts by Suppressing ALK-5/Smad2/3 Signaling Pathway. Exp. Cel Res. 362, 17–27. 10.1016/j.yexcr.2017.10.004 28987540

[B93] Yang T.-H. Gingery A. Thoreson A. R. Larson D. R. Zhao C. Amadio P. C. (2018). Triamcinolone Acetonide Affects TGF-β Signaling Regulation of Fibrosis in Idiopathic Carpal Tunnel Syndrome. BMC Musculoskelet. Disord. 19, 342. 10.1186/s12891-018-2260-y 30243295 PMC6151186

[B94] Yuan Y. Zhang Y. Han X. Li Y. Zhao X. Sheng L. (2017). Relaxin Alleviates TGFβ1-Induced Cardiac Fibrosis via Inhibition of Stat3-dependent Autophagy. Biochem. Biophysical Res. Commun. 493, 1601–1607. 10.1016/j.bbrc.2017.09.110 28942152

[B95] Zeng Q. Macri L. K. Prasad A. Clark R. A. F. Zeugolis D. I. Hanley C. (2011). “Skin Tissue Engineering,” in Comprehensive Biomaterials. Editor Ducheyne P. (Oxford: Elsevier), 467–499. 10.1016/b978-0-08-055294-1.00186-0

[B96] Zeugolis D. I. (2021). Bioinspired *In Vitro* Microenvironments to Control Cell Fate: Focus on Macromolecular Crowding. Am. J. Physiology-Cell Physiol. 320, C842–C849. 10.1152/ajpcell.00380.2020 33656930

[B97] Zwaagstra J. C. Sulea T. Baardsnes J. Lenferink A. E. G. Collins C. Cantin C. (2012). Engineering and Therapeutic Application of Single-Chain Bivalent TGF-β Family Traps. Mol. Cancer Ther. 11, 1477–1487. 10.1158/1535-7163.mct-12-0060 22562986

