# Supplementary material for: Adapting the Scar-in-a-Jar to Skin Fibrosis and Screening Traditional and Contemporary Anti-Fibrotic Therapies
Source: Front Bioeng Biotechnol. 2021 Oct 26;9:756399. doi: 10.3389/fbioe.2021.756399 (PMC8576412; doi:10.3389/fbioe.2021.756399)
Supplement: Supplementary file 1 [file DataSheet1.docx]

**Supplementary information**

**Supplementary Table S1:** Representative examples of pharmacological therapies for skin fibrosis according to the most used mechanisms of action.

* Indicates molecules used in this study.

| Molecule class | Molecule | Mechanism of action | Summary |
| --- | --- | --- | --- |
| Corticosteroids | Triamcinolone acetonide* | Immunosuppressant, inhibits NF-κB, cellular proliferation and collagen synthesis | “Gold standard” in keloid and hypertrophic scar treatment; presents many side-effects [1, 2] |
|  | Dexamethasone |  | Decrease of collagen type I and αSMA expression in an in vivo model of fibrosis [3] |
|  | Methylprednisolone |  | Decrease of keloid fibroblast proliferation, migration and fibrotic markers in vitro [4] |
| Inhibitors of cell proliferation | Trichostatin A* | Histone deacetylase inhibitor | Prevention of ECM accumulation in vivo [5] |
|  | 5-Fluorouracil | Antimetabolite | Similar clinical effectivity to triamcinolone acetonide; less side-effects [2] |
|  | Tamoxifen | RNA transcription / cell cycle modulation | Inhibition of dermal fibroblast proliferation in vitro [6]  Decrease of keloid fibroblasts and collagen fibres in early clinical trials [7] |
| Inhibitors of collagen crosslinking / deposition | β-aminopropionitrile* | Lysyl oxidase inhibitor | Failure to inhibit basal or TGFβ-stimulated in vitro fibroplasia [8] |
|  | Verapamil | Calcium channel blocker | Similar clinical effectivity to triamcinolone acetonide; smaller incidence of telangiectasia and skin atrophy [9] |
|  | Decorin | Modulation of collagen fibrillogenesis / degradation and pro-fibrotic growth factor signalling (TGFβ1, CTGF/CNN2, myostatin and thrombospondin) | Decreased collagen deposition and increased MMP expression in vitro [10]; inhibits scar and fibrosis formation in multiple experimental models by suppressing inflammation and TGFβ pathways [11] |
| Inhibitors of TGFβ (superfamily) signalling | TGFβ trap (T22d35 / T122bt)* | TGFβ1/2 signalling blocker | T122bt decreased collagen deposition in our in vitro study |
|  | Activin IIB receptor inhibitor* | Activin IIB receptor inhibition  (Activins A&B, myostatin, GDF-11) | Decreased collagen deposition in our in vitro study |
|  | Botulinum toxin type A | TGFβ1/Smad and ERK signalling inhibition | Effective in preventing and treating scar hypertrophy in vivo [12]; early clinical trials corroborate this [13, 14] |
| Pleiotropic inhibitors of fibrotic activation | Serelaxin* | Smad 2/3 signalling inhibition, inhibition of the inflammasome, increased expression of MMPs | In vitro and in vivo studies observed decreased fibrotic markers; mixed reports in clinical trials [15, 16] |
|  | Pirfenidone* | TGFβ1 / PDGF signalling inhibition, anti-inflammatory activity | Suppression of keloid-derived fibroblast contraction [17]  Clinical trial phase III for treatment of keloid scars [18] |
|  | Rapamycin | Inhibitor of the mTOR complex and PDGF signalling | Reduced collagen expression in vitro [19] / in vivo [20]  Decreased facial angiofibroma severity index in clinical trials [21] |

**Supplementary Table S2:** Representative examples of current *in vitro* models of skin fibrosis and their advantages and disadvantages.

| Model | Mechanism of action | Advantages | Disadvantages |
| --- | --- | --- | --- |
| Monolayer of cultured fibroblasts | Cultured normal dermal fibroblasts [22] or derived from keloids / hypertrophic scars [23, 24] | Ease to obtain and to use, simple methodology and standard culture conditions | Low ECM deposition in a useful time window for screening  Limited physiological relevance |
| Hyperconfluent models with TGFβ supplementation | Dermal fibroblasts cultured under long periods of time and supplemented with TGFβ1 [8] and 2 [25] | Induces myofibroblast transformation and closely resembles a fibrotic phenotype  Current industry testing standard | Long culture times (4-6 weeks)  Limited physiological relevance |
| Macromolecular crowding models | Dermal fibroblasts cultured under macromolecular crowding conditions [26-28] | High ECM deposition in a small time-frame  Expression of fibrotic markers  Useful for high-throughput screening | Absence of cellular crosstalk  Do not represent a 3D environment |
| 3D models | Dermal cells incorporated into pre-made matrixes [28-31]  Co-culture of different cells in layers [32-37] | Better replication of the skin’s natural hierarchy  Interaction of cells and matrix mimic *in vivo* conditions | Increased production costs and requirements for regulatory approval  Increased complexity hinders standardisation and high-throughput screening |
| Ex vivo models | Skin explants (from healthy and pathological tissues) cultured in vitro [34, 38-41] | Reconstitution of the skin’s architecture and cell populations, with their inherent cellular crosstalk and molecular signalling | Difficult to maintain for long periods of time due to the absence of in vivo components and circulation  Lack of tissue donors |
| Organoids and organs-on-a-chip | Use of tissue engineering and microfluidics to replicate multi-tissue and organ interaction through organoids [10, 42, 43] or organs-on-a-chip [44-48] | Replication of the skin’s hierarchical complexity and different tissue / organ interaction in vitro | Increased complexity can hinder their replication in different laboratorial settings |

**Supplementary Table S3:** TGF*β* traps were assessed for neutralisation of different TGF*β* isoforms, namely TGF*β*1, TGF*β*2 and TGF*β*3. Both traps neutralised TGF*β*1 and TGF*β*3, with the IC_50_**-**values of 2.67 nM and 0.45 nM, respectively, for T22d35 and 0.07 nM and 0.06 nM, respectively, for T122bt. The T22d35 trap did not neutralise TGF*β*2, as expected, since T*β*RII has a low affinity for TGF*β*2. In contrast, the heterovalent T122bt trap was able to neutralise TGF*β*2 with reasonable potency [IC_50_**-**values of 8.18 nM]. The TGF*β* luciferase assay showed that these traps are very potent (inhibition at very low to sub nM levels) in their capacity to neutralize TGF*β* isoforms. The reported potencies are average IC50s and standard deviations (SD) from at least 2 experiments, done with triplicate samples.

|  | TGFβ1 | | TGFβ2 | | TGFβ3 | |
| --- | --- | --- | --- | --- | --- | --- |
|  | **IC50 (nM)** | **SD** | **IC50 (nM)** | **SD** | **IC50 (nM)** | **SD** |
| T22d35 | 2.67 | 0.84 | negative | - | 0.45 | 0.07 |
| T122bt | 0.07 | 0.04 | 8.18 | 0.95 | 0.06 | 0.03 |


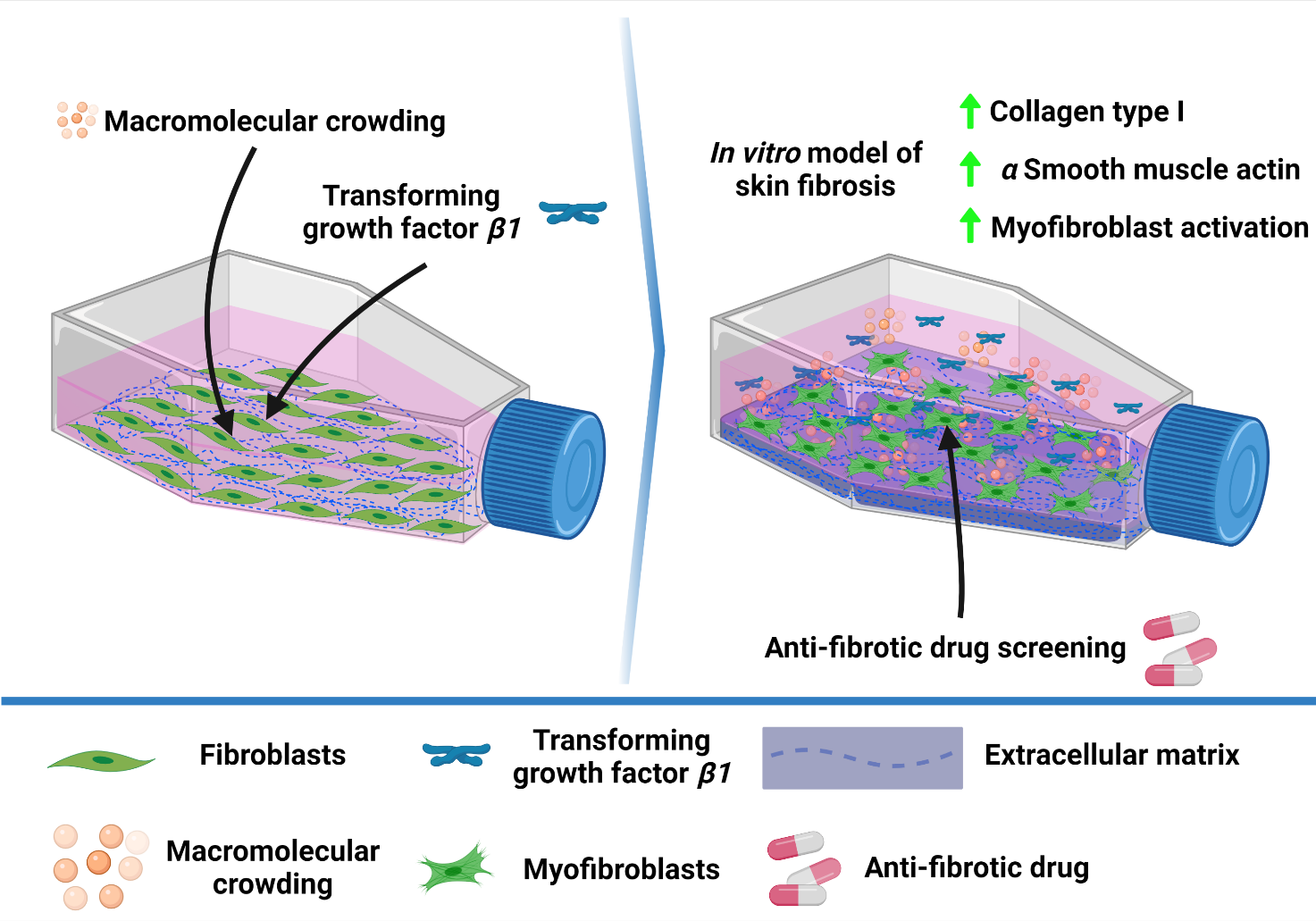


**Supplementary Figure S1:** Supplementation of macromolecular crowding and TGF*β*1 resulted in increased collagen type I deposition and transformation of dermal fibroblasts into αSMA expressing myofibroblasts. This *in vitro* fibrosis model was used for anti-fibrotic drug screening.


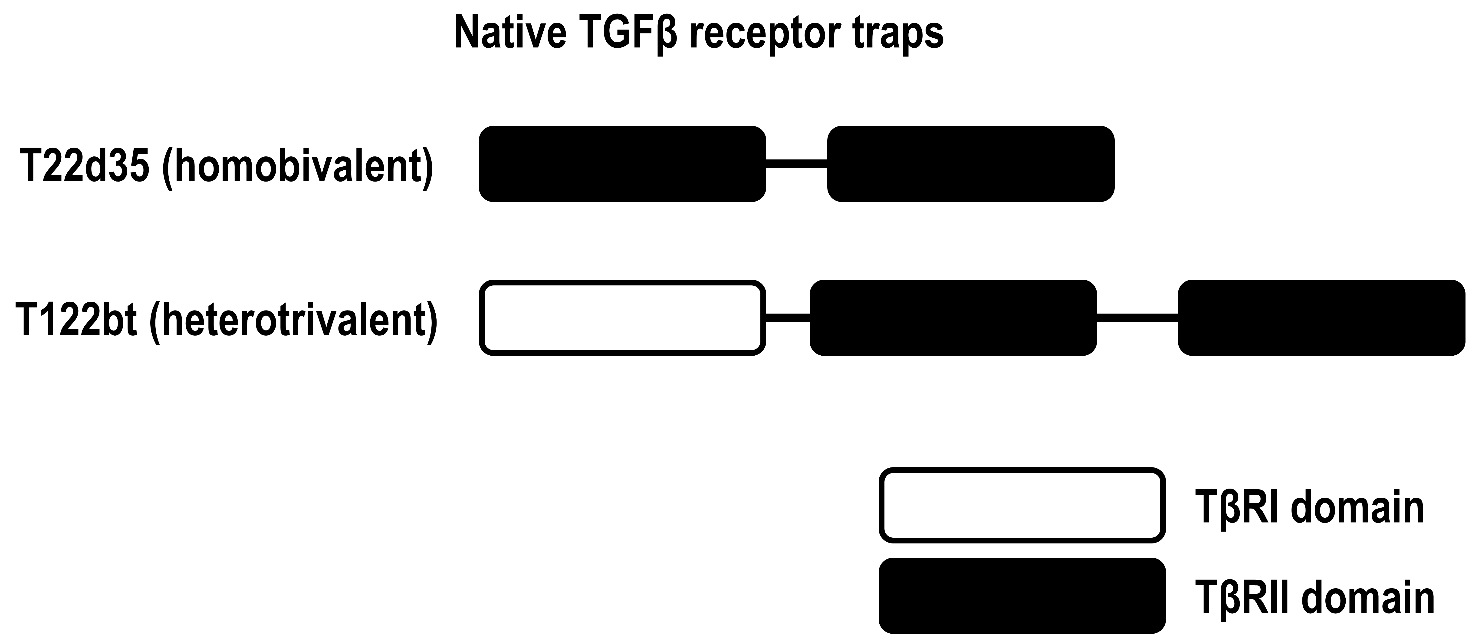


**Supplementary Figure S2:** Schematic of TGFβ receptor-based, single-chain traps, T22d35 and T122bt. The TβRI and TβRII receptor domains that bind TGFβ, are fused via flexible, intervening linkers derived from native receptor sequences [49, 50].


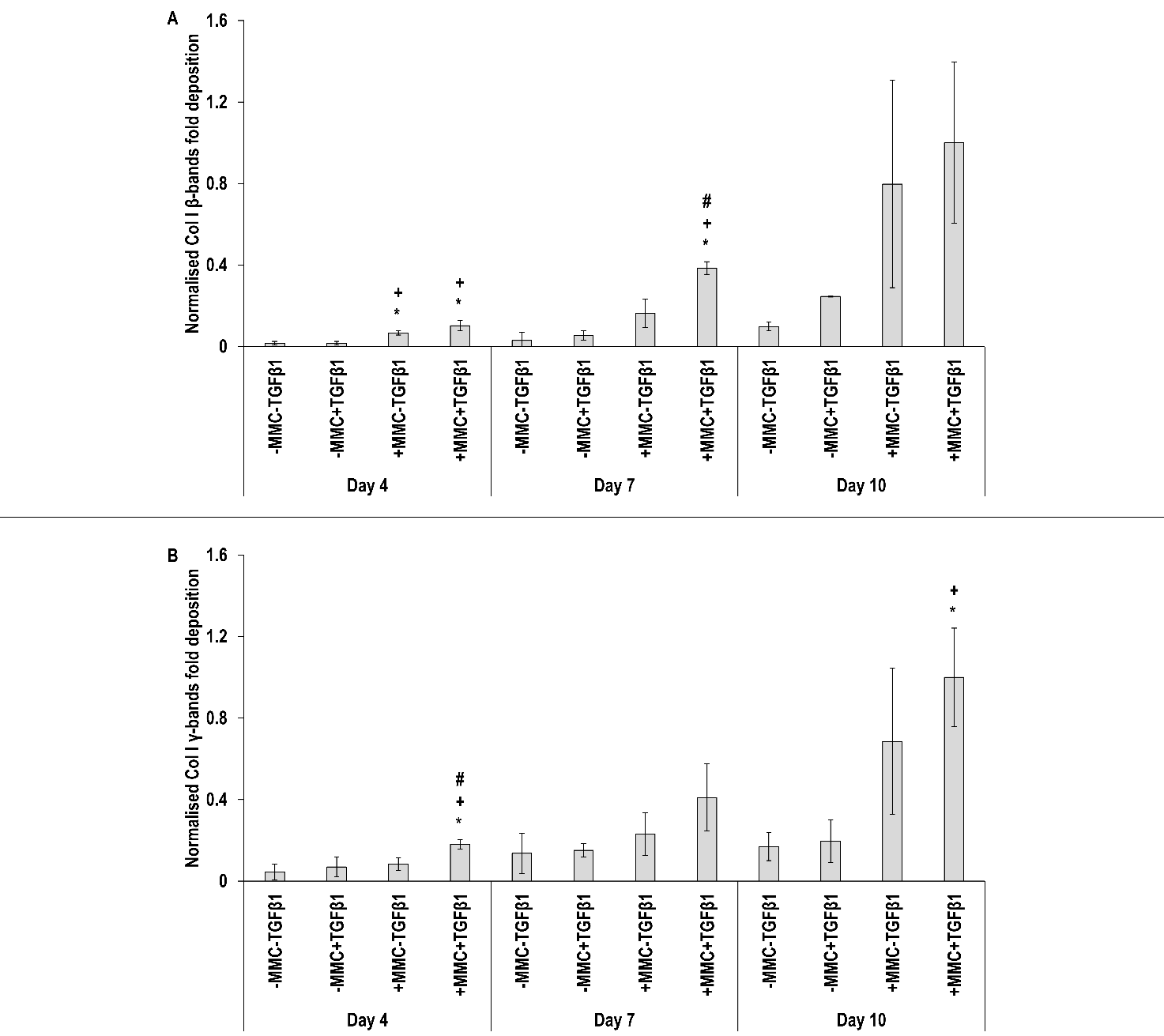


**Supplementary Figure S3:** MMC and TGFβ1 increase collagen deposition. Densitometry of SDS-PAGE for comparison of the expression of collagen I β11(I), β12(I) (A) and γ(I) bands (B) after supplementation with +MMC+TGFβ1 for up to 10 days showed that MMC increased collagen type I deposition and +MMC+TGFβ1 increased further collagen type I deposition in DF cultures. Col I STD: 0.1 mg/ml. Statistical analysis through one-way ANOVA and Tukey’s post-hoc comparison test, *: p < 0.05 denotes a significant difference when compared to the -MMC-TGFβ1 group of the respective time point, +: p < 0.05 denotes a significant difference when compared to the -MMC+TGFβ1 group of the respective time point, #: p < 0.05 denotes a significant difference when compared to the +MMC-TGFβ1 group of the respective time point. n = 3.


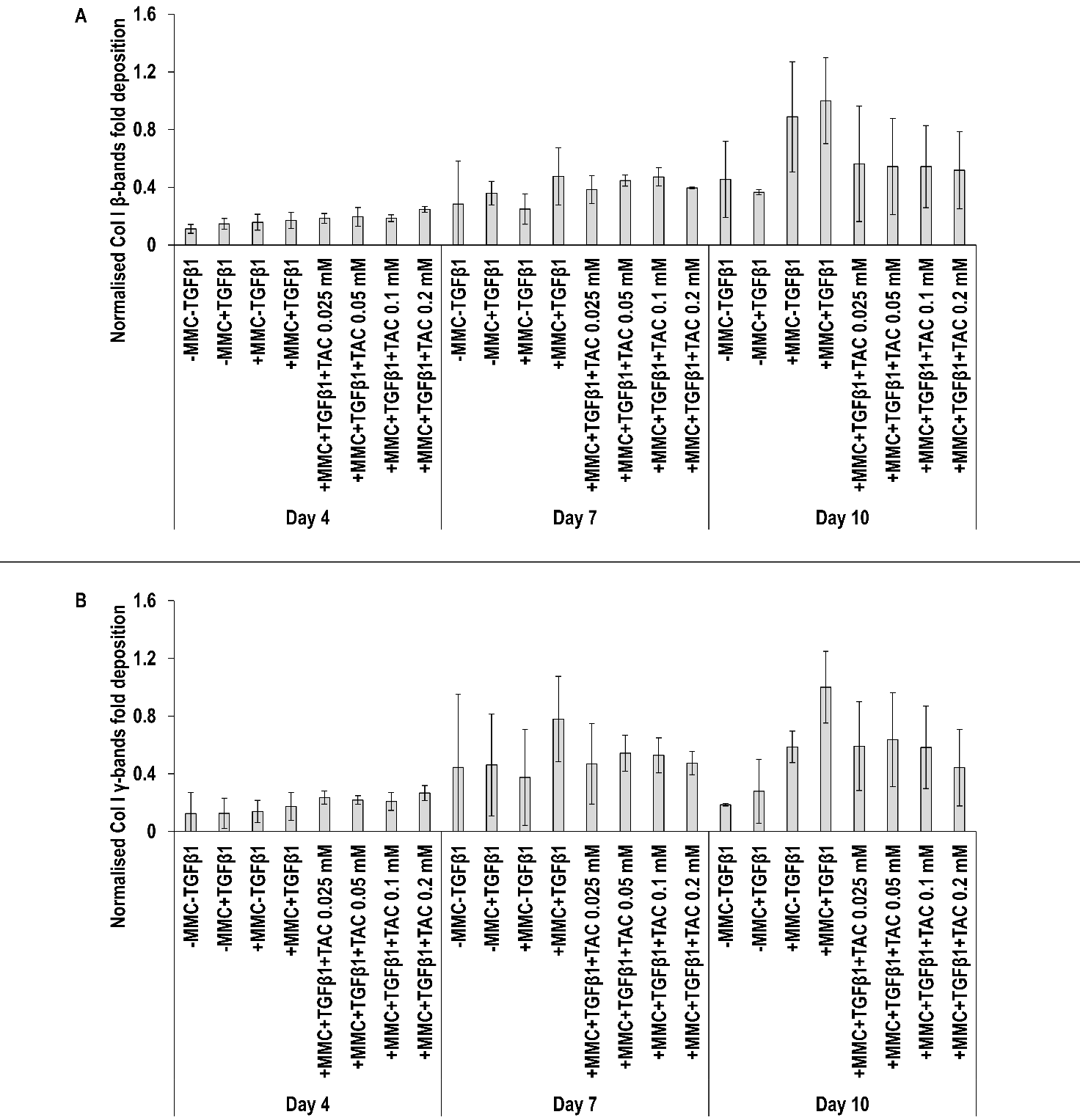


**Supplementary Figure S4:** TAC has no effect on collagen β11(I), β12(I) dimer and γ(I) trimer deposition. Densitometry of SDS-PAGE for comparison of the expression of collagen I β11(I), β12(I) (A) and γ bands (B) after supplementation with +MMC+TGFβ1 and TAC for up to 10 days revealed no significant differences between the +MMC+TGFβ1 groups and any of the +MMC+TGFβ1+TAC concentration groups. Col I STD: 0.1 mg/ml. n = 3.


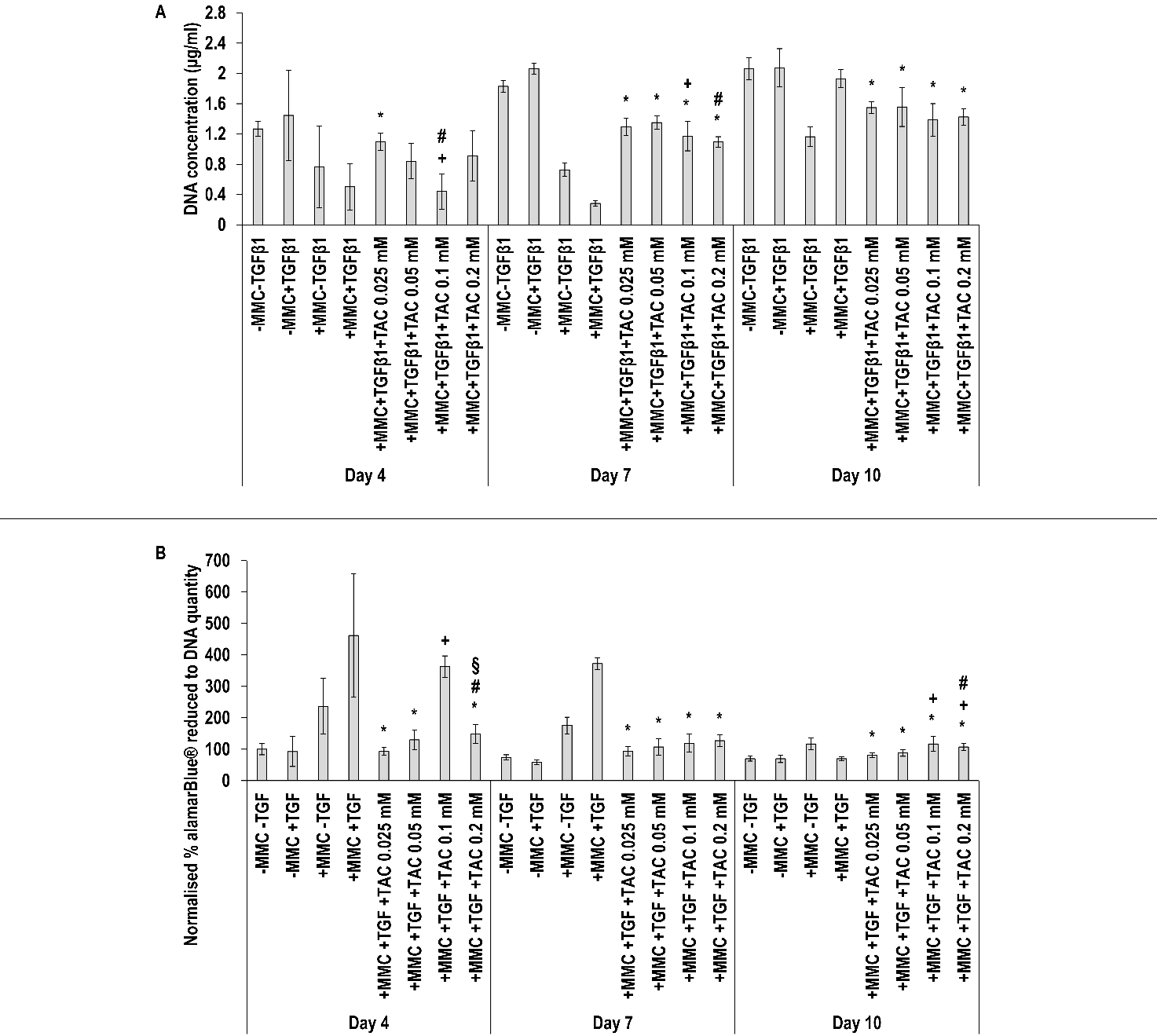


**Supplementary Figure S5:** TAC affects cell proliferation and metabolic activity. At day 4 and 7, the +MMC+TGFβ1+TAC groups exhibited significantly higher DNA concentration (A) and significantly lower metabolic activity (B) than the +MMC+TGFβ1 group and at day 10, the +MMC+TGFβ1+TAC groups exhibited significantly lower DNA concentration (A) and significantly higher metabolic activity (B) than the +MMC+TGFβ1 group. One-way ANOVA and Tukey’s post-hoc comparison test or Kruskal Wallis and Mann Whitney post-hoc analysis were conducted as appropriate. *: p < 0.05 indicates a statistically significant difference when compared to the +MMC+TGFβ1 group of the respective time point. +: p < 0.05 indicates a statistically significant difference when compared to the +MMC+TGFβ1+TAC 0.025 mM group of the respective time point. #: p < 0.05 indicates a statistically significant difference when compared to the +MMC+TGFβ1+TAC 0.05 mM group of the respective time point. §: p < 0.05 indicates a statistically significant difference when compared to the +MMC+TGFβ1+TAC 0.1 mM group of the respective time point. n = 3.


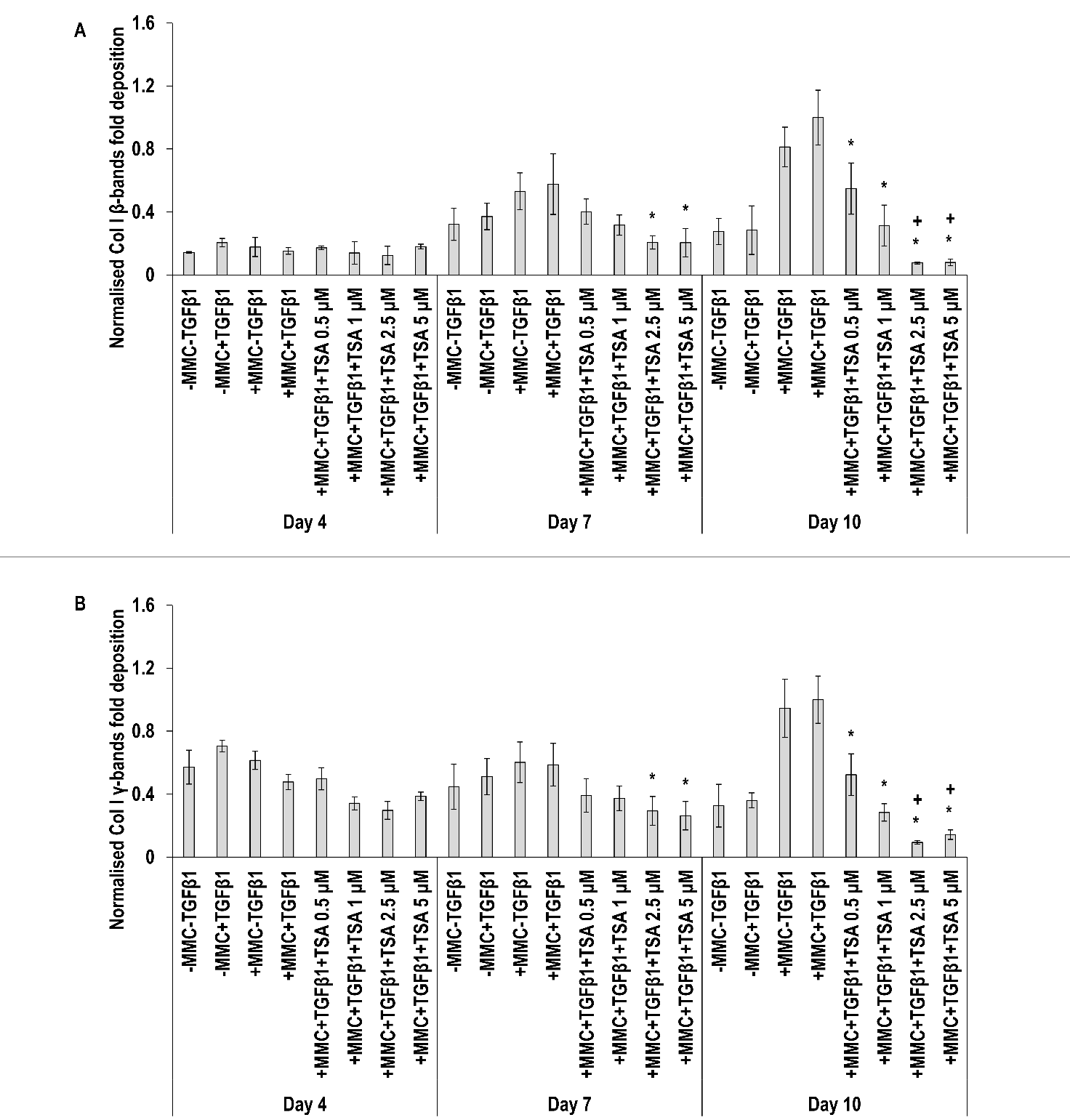


**Supplementary Figure S6:** High concentrations of TSA reduce collagen deposition. The 2.5 µM and 5 µM TSA concentrations in +MMC+TGFβ1 at day 7 and day 10 induced the lowest collagen deposition in comparison to +MMC+TGFβ1 group, as revealed by densitometry of β11(I), β12(I) (A) and γ(I) bands (B). Col I STD: 0.1 mg/ml. One-way ANOVA and Tukey’s post-hoc comparison tests were conducted. *: p < 0.05 indicates a statistically significant difference when compared to the +MMC+TGFβ1 group of the respective time point. +: p < 0.05 indicates a statistically significant difference when compared to the +MMC+TGFβ1+TSA 0.5 μM group of the respective time point. n = 3.


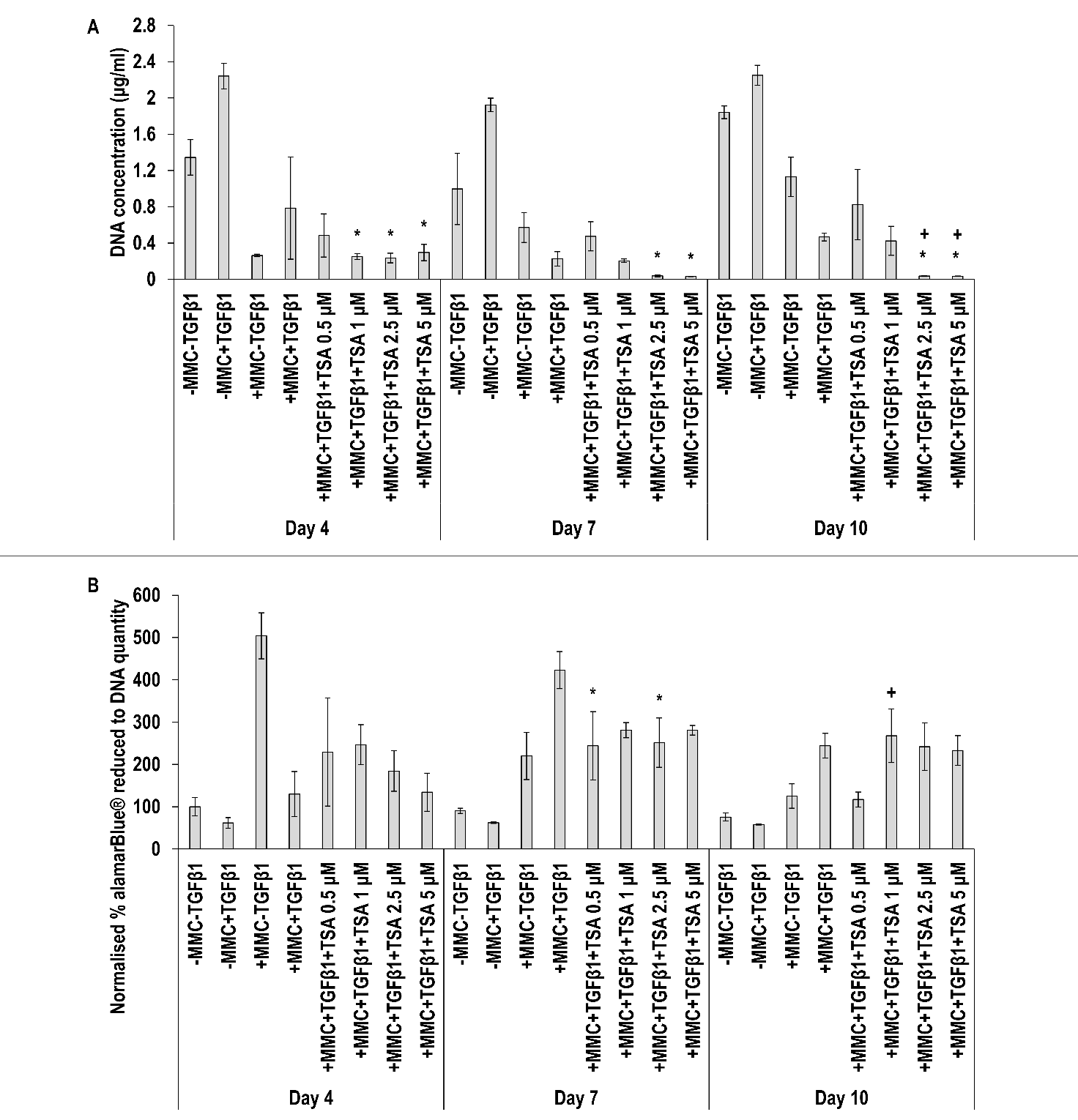


**Supplementary Figure S7:** High concentrations of TSA decrease cell proliferation. At all time points, the 2.5 µM and 5 µM TSA concentrations in +MMC+TGFβ1 resulted in reduced DNA concentration in comparison to the +MMC+TGFβ1 group (A). At day 7, the 0.5 and 2.5 µM TSA concentration in +MMC+TGFβ1 resulted in reduced metabolic activity in comparison to the +MMC+TGFβ1 group (B). One-way ANOVA and Tukey’s post-hoc comparison tests were conducted. *: p < 0.05 indicates a statistically significant difference when compared to the +MMC+TGFβ1 group of the respective time point. +: p < 0.05 indicates a statistically significant difference when compared to the +MMC+TGFβ1+TSA 0.5 μM group of the respective time point. n = 3.


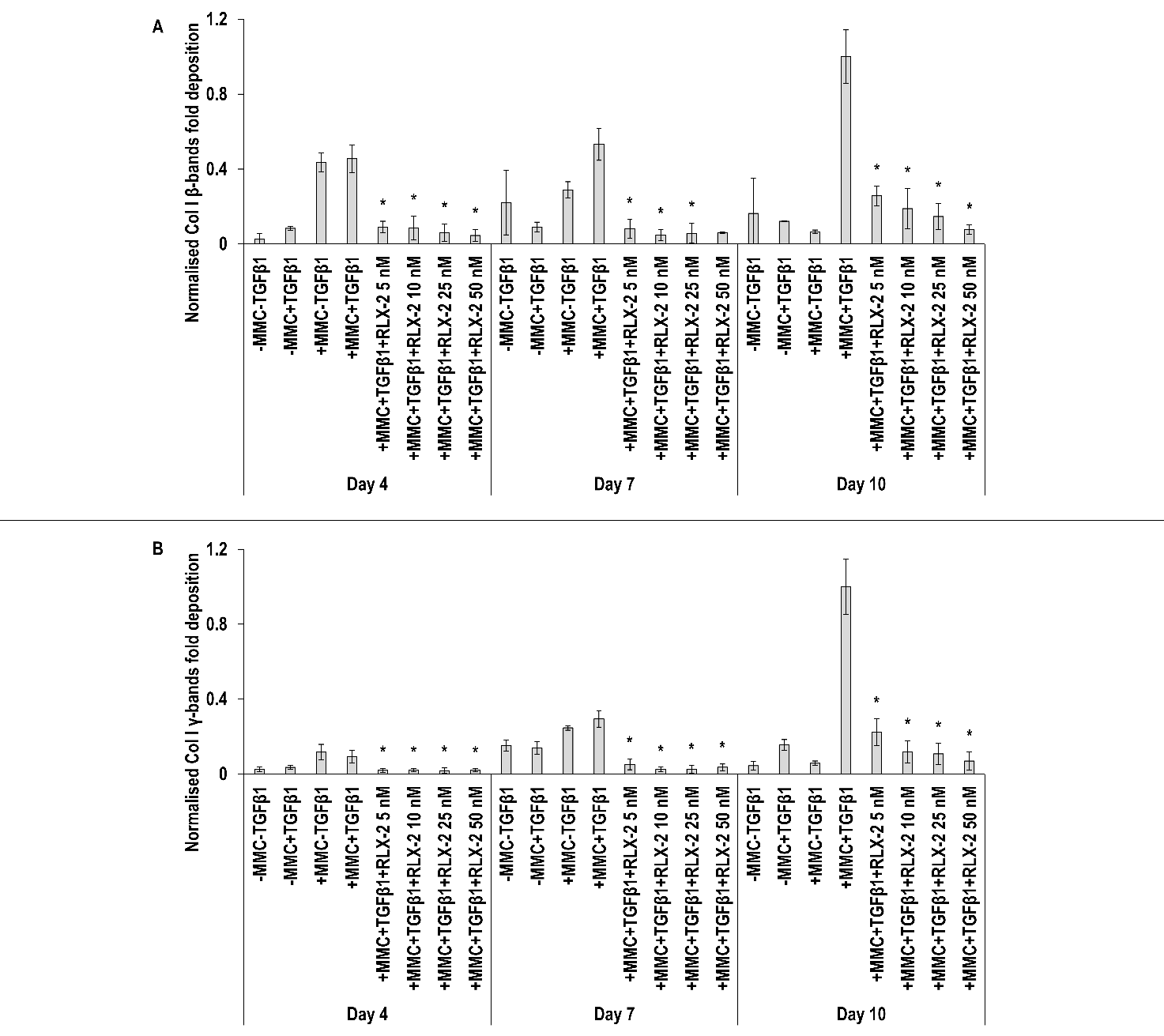


**Supplementary Figure S8:** RLX-2 reduces collagen deposition at all time points. All RLX-2 concentrations in +MMC+TGFβ1 at all time points resulted in significant (p < 0.05) decrease of collagen deposition, as judged by densitometry analysis of β11(I), β12(I) (A) and γ(I) bands (B). Col I STD: 0.1 mg/ml. One-way ANOVA and Tukey’s post-hoc comparison tests were conducted. *: p < 0.05 indicates a statistically significant difference when compared to the +MMC+TGFβ1 group of the respective time point. n = 3.


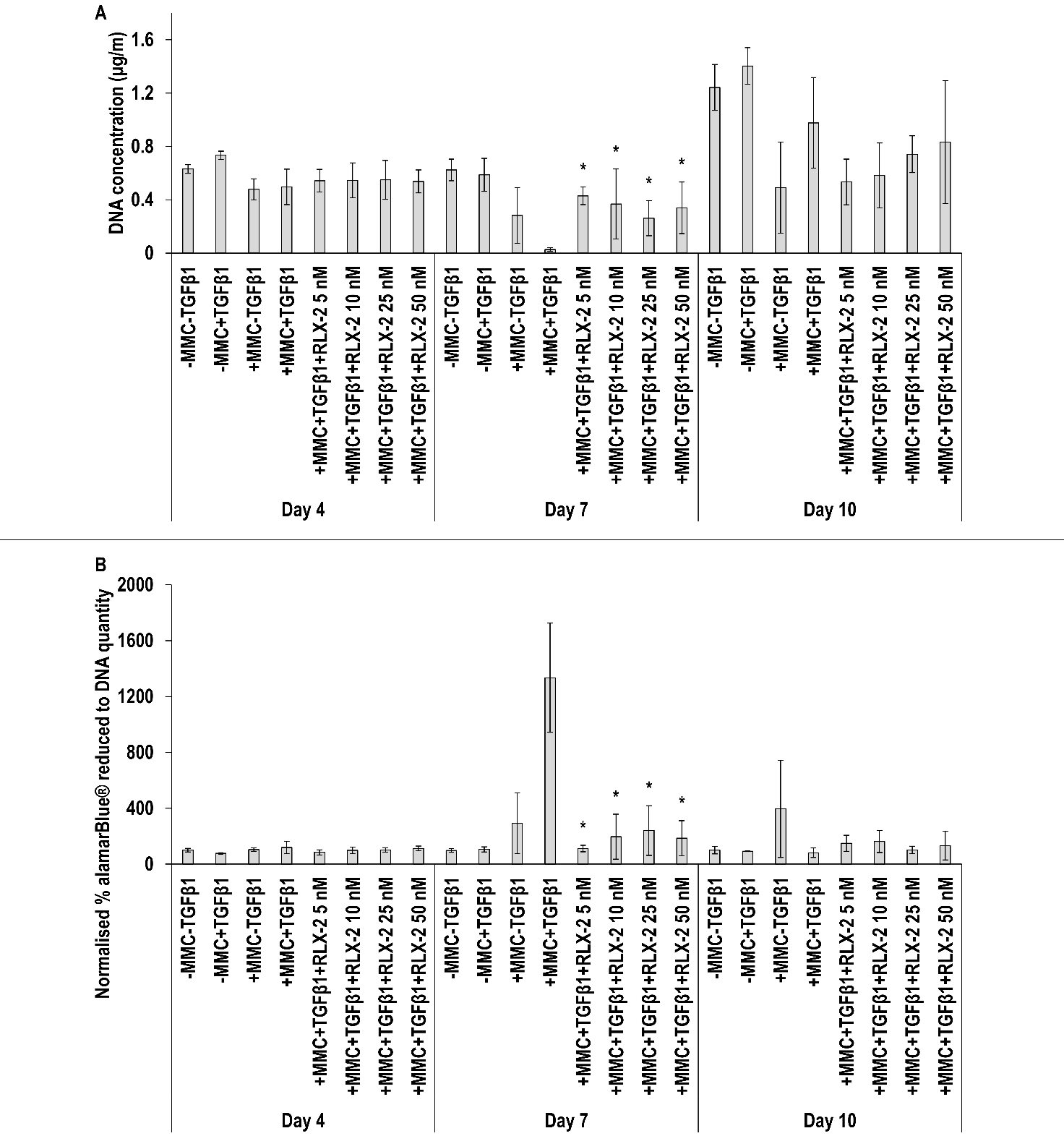


**Supplementary Figure S9:** RLX-2 only impacts cell proliferation and metabolic activity at day 7. At day 7, all RLX-2 concentrations in +MMC+TGFβ1 significantly increased DNA concentration and significantly decreased metabolic activity, in comparison to the +MMC+TGFβ1 group. One-way ANOVA and Tukey’s post-hoc comparison tests were conducted. *: p < 0.05 indicates a statistically significant difference when compared to the +MMC+TGFβ1 group of the respective time point. n = 3.


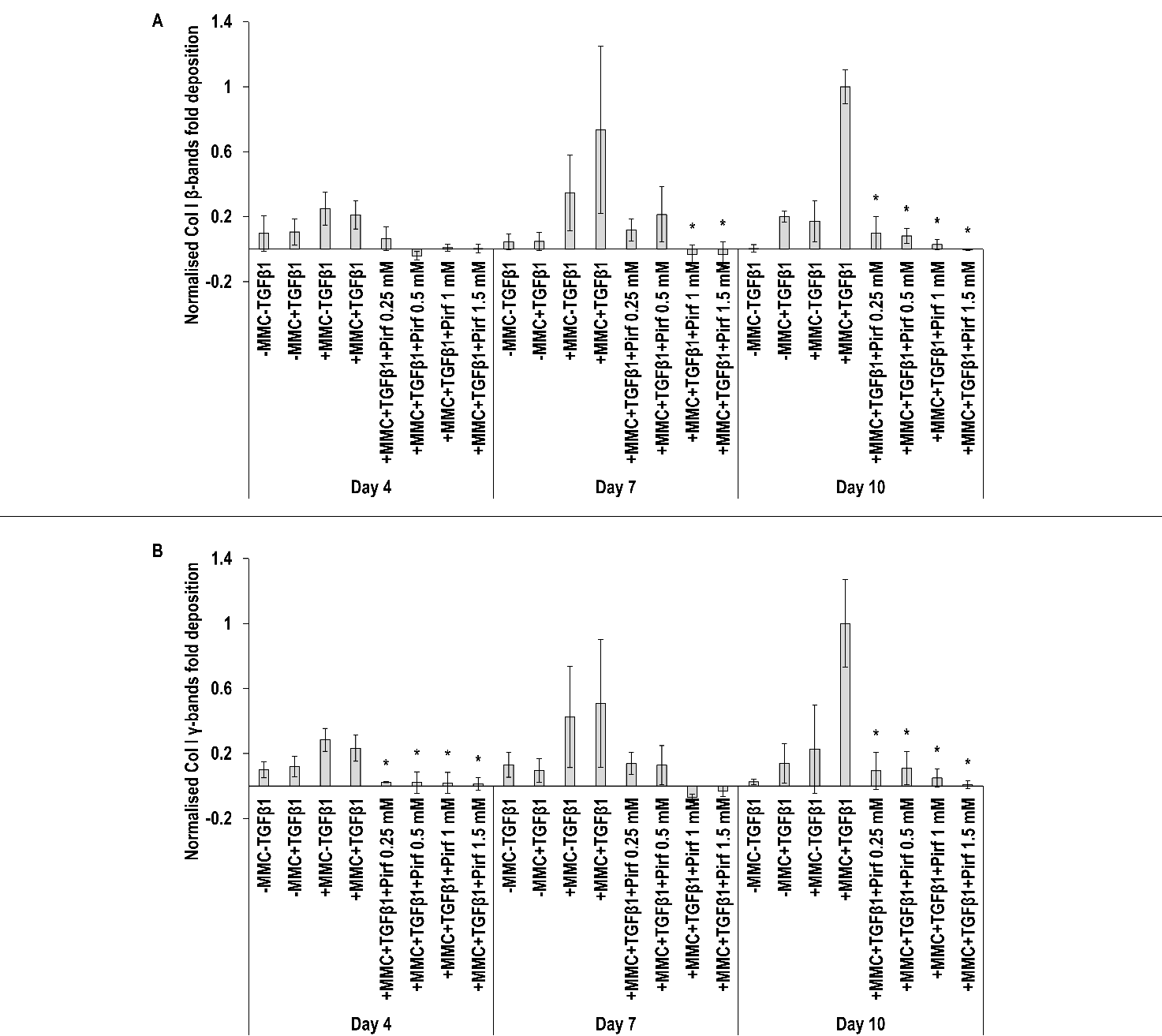


**Supplementary Figure S10:** Pirf reduces collagen deposition. The 1 mM and 1.5 mM Pirf concentrations in +MMC+TGFβ1 resulted in significant decrease of collagen deposition, as judged by densitometry analysis of β11(I), β12(I) (A) and γ(I) bands (B). Col I STD: 0.1 mg/ml. One-way ANOVA and Tukey’s post-hoc comparison tests were conducted. *: p < 0.05 indicates a statistically significant difference when compared to the +MMC+TGFβ1 group of the respective time point. n = 3.


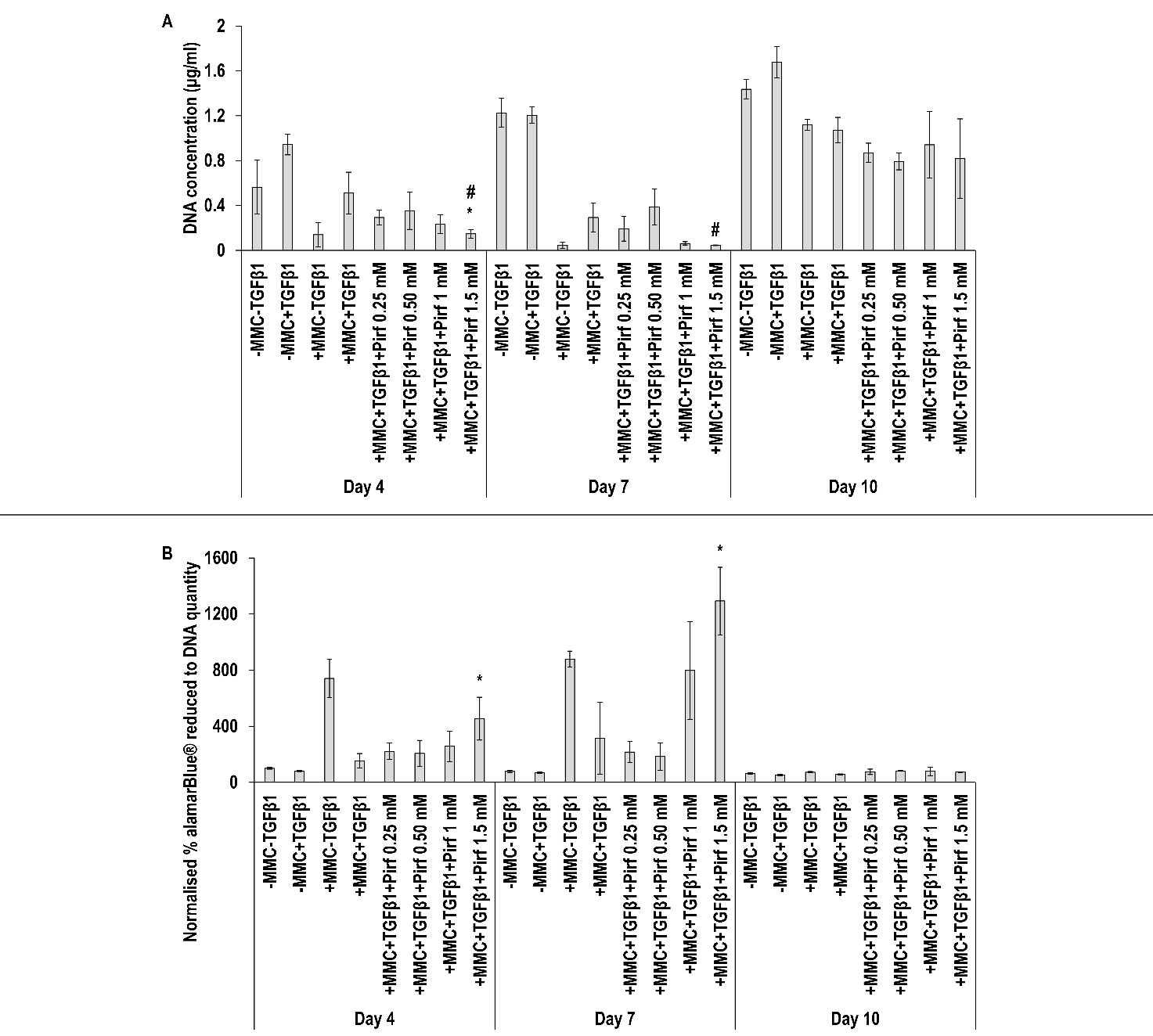


**Supplementary Figure S11:** The highest concentration of Pirf affects cell proliferation and metabolic activity. The 1.5 mM Pirf concentration in +MMC+TGFβ1 resulted in decreased DNA concentration and increased metabolic activity at day 4 and day 7 in comparison to the +MMC+TGFβ1 group. One-way ANOVA and Tukey’s post-hoc comparison tests were conducted. *: p < 0.05 indicates a statistically significant difference when compared to the +MMC+TGFβ1 group of the respective time point. #: p < 0.05 indicates a statistically significant difference when compared to the +MMC+TGFβ1+Pirf 0.50 mM group of the respective time point. n = 3.


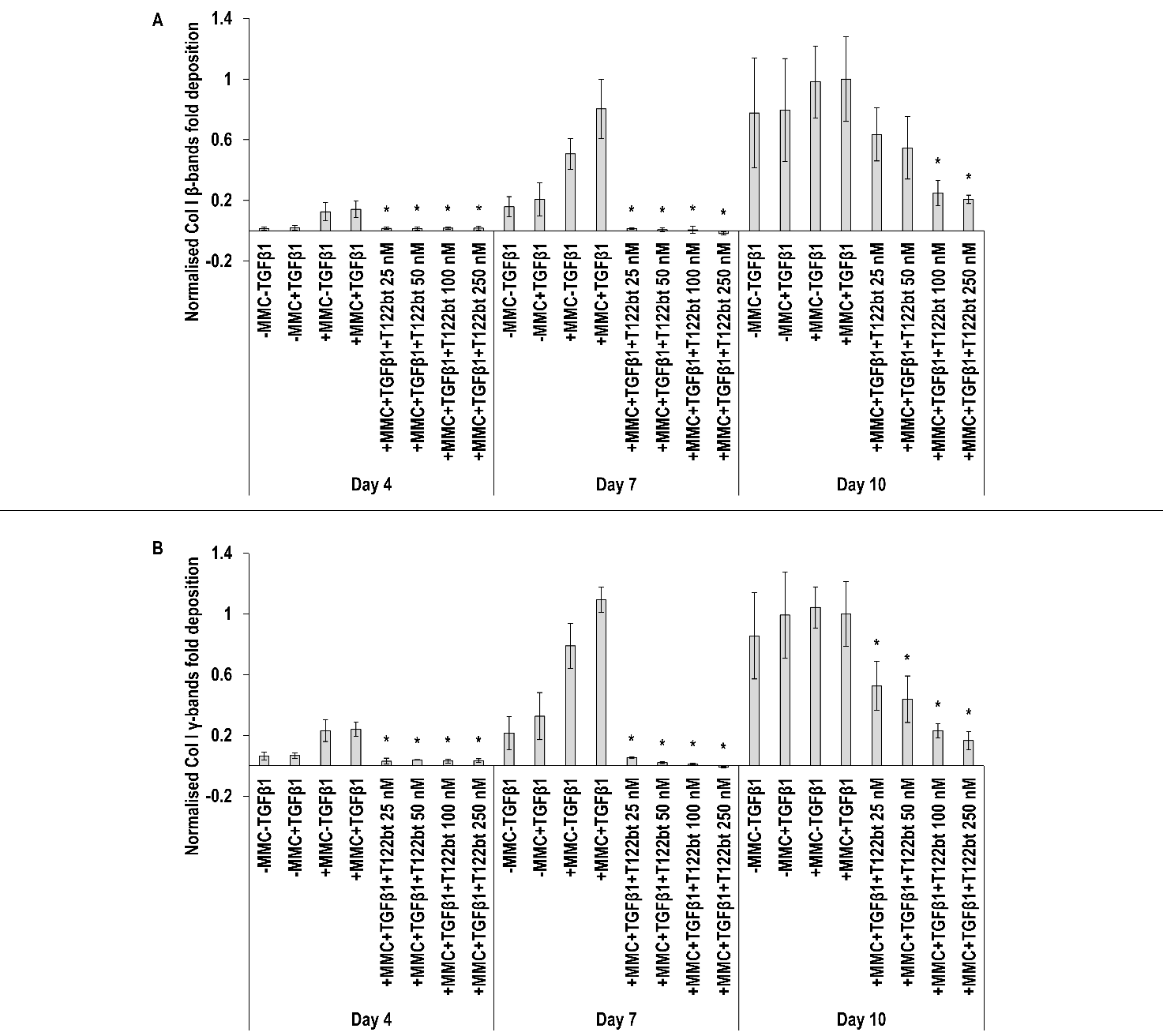


**Supplementary Figure S12:** High concentrations of TGFβ type II receptor-based trap (T122bt) reduce collagen deposition. The 100 and 250 nM T122bt concentrations in +MMC+TGFβ1 significantly reduced collagen deposition at all time points, in comparison to +MMC+TGFβ1 group, as judged by densitometry of β11(I), β12(I) (A) and γ(I) bands (B). Col I STD: 0.1 mg/ml. One-way ANOVA and Tukey’s post-hoc comparison test or Kruskal Wallis and Mann Whitney post-hoc analysis were conducted, as appropriate. *: p < 0.05 indicates a statistically significant difference when compared to the +MMC+TGFβ1 group of the respective time point. n = 3.


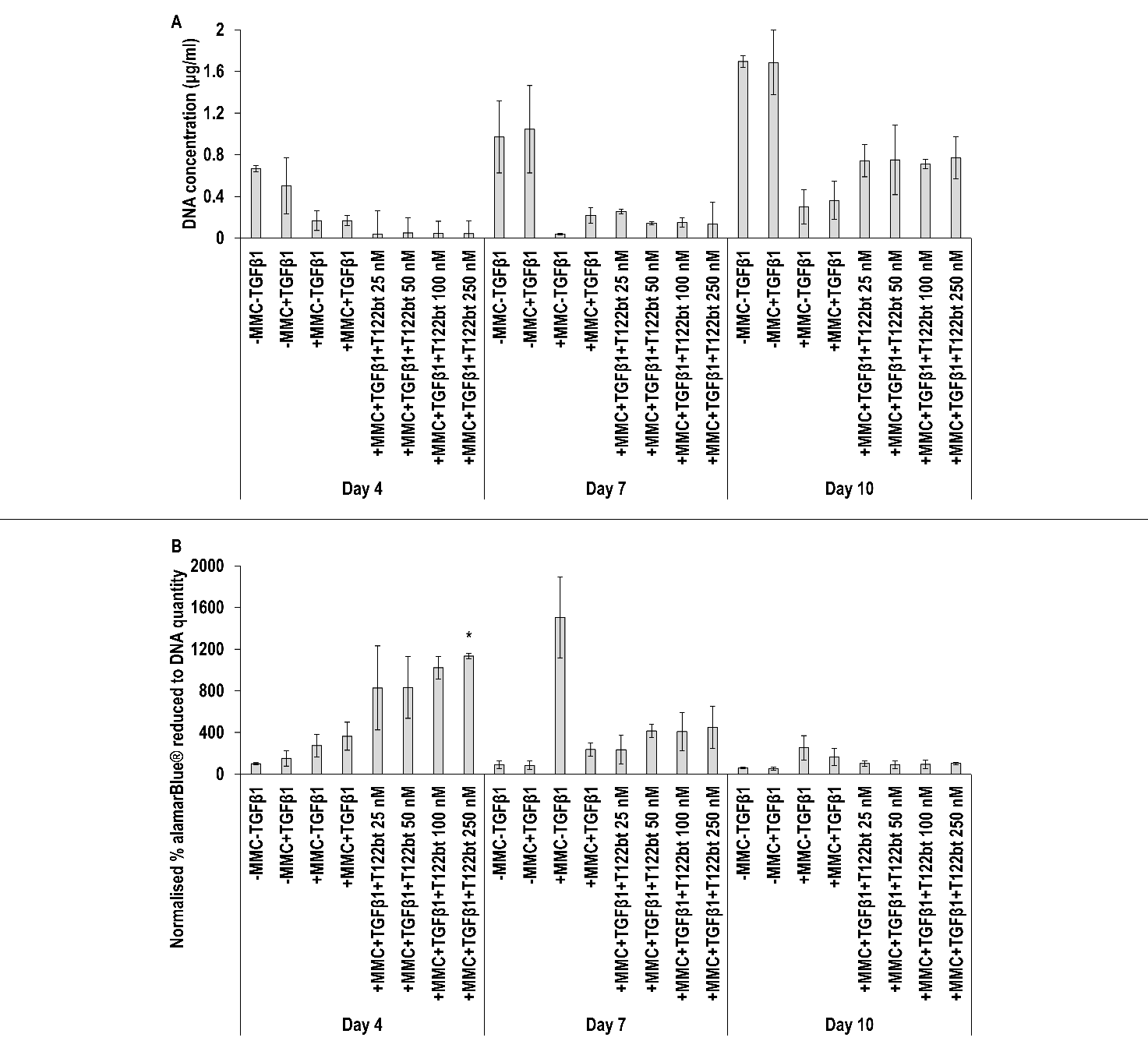


**Supplementary Figure S13:** TGFβ type II receptor-based trap (T122bt) does not affect cell proliferation or metabolic activity. No T122bt concentrations in +MMC+TGFβ1 induced any significant differences at any time point in DNA concentration (A) and metabolic activity (B), apart from the 250 nM T122bt concentration at day 4, in comparison to the +MMC+TGFβ1 group. One-way ANOVA and Tukey’s post-hoc comparison tests were conducted. *: p < 0.05 indicates a statistically significant difference when compared to the +MMC+TGFβ1 group of the respective time point. n = 3.


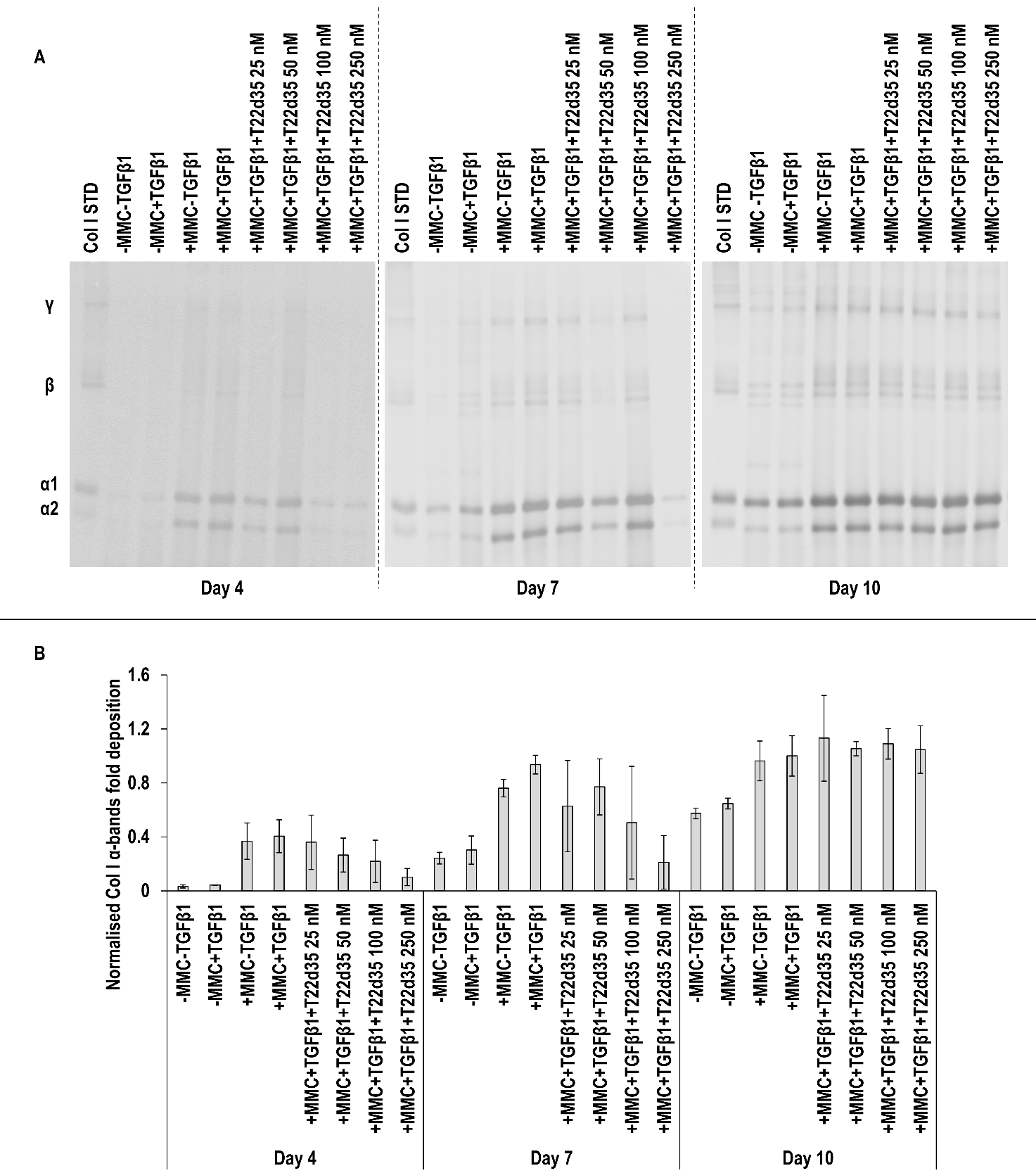


**Supplementary Figure S14:** TGFβ type II receptor-based trap (T22d35) does not suppress collagen type I deposition. None of the T22d35 concentrations in +MMC+TGFβ1 significantly reduced collagen deposition in comparison to +MMC+TGFβ1 group, as judged by SDS-PAGE (A) and densitometry analysis of α(I)1 and α(I)2 bands (B). Col I STD: 0.1 mg/ml. One-way ANOVA and Tukey’s post-hoc comparison tests were conducted. n = 3.


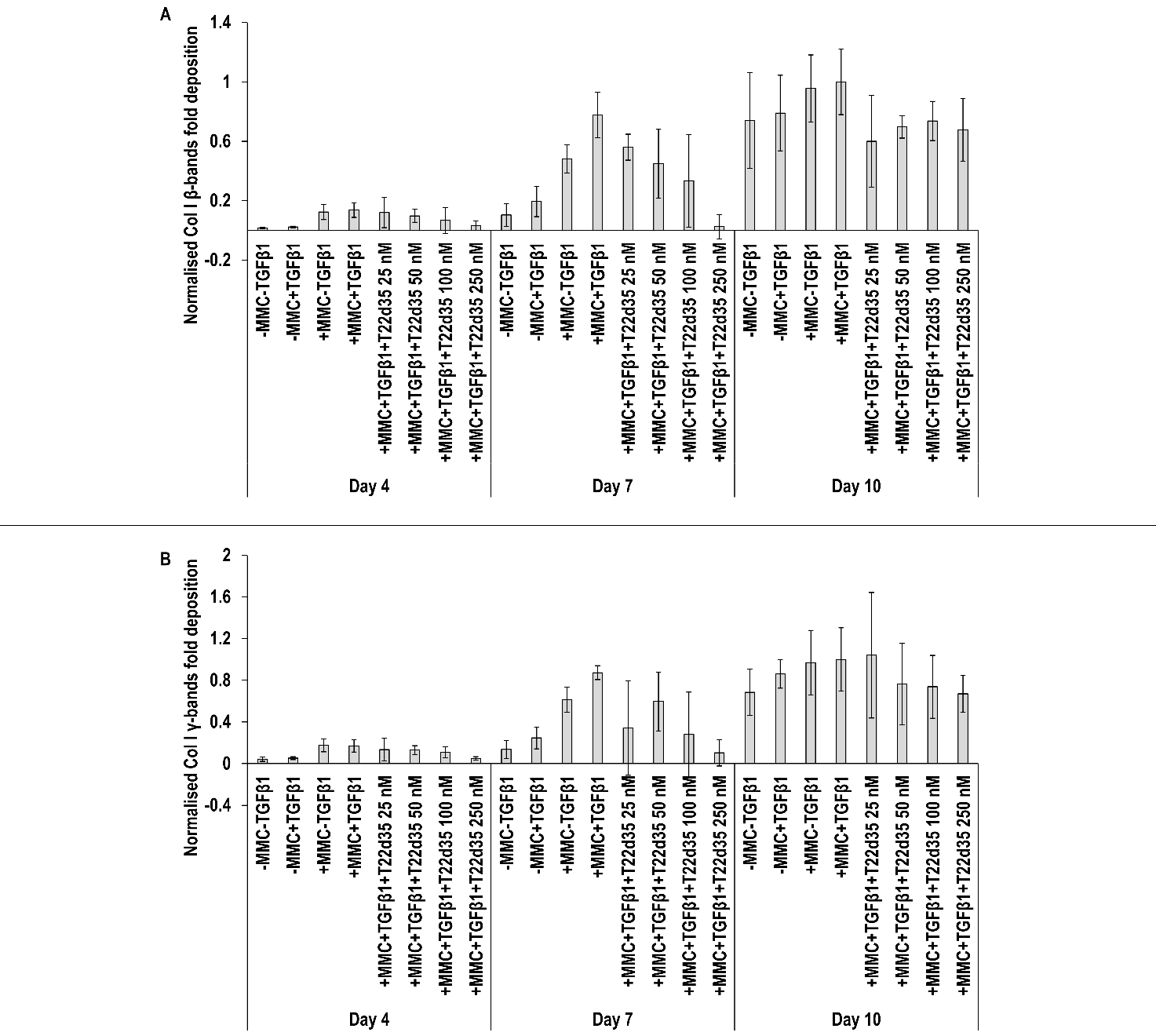


**Supplementary Figure S15:** TGFβ type II receptor-based trap (T22d35) does not suppress collagen type I deposition. None of the T22d35 concentrations in +MMC+TGFβ1 significantly reduced collagen deposition in comparison to +MMC+TGFβ1 group, as judged by densitometry analysis of β11(I), β12(I) (A) and γ(I) bands (B). Col I STD: 0.1 mg/ml. One-way ANOVA and Tukey’s post-hoc comparison tests were conducted. n = 3.


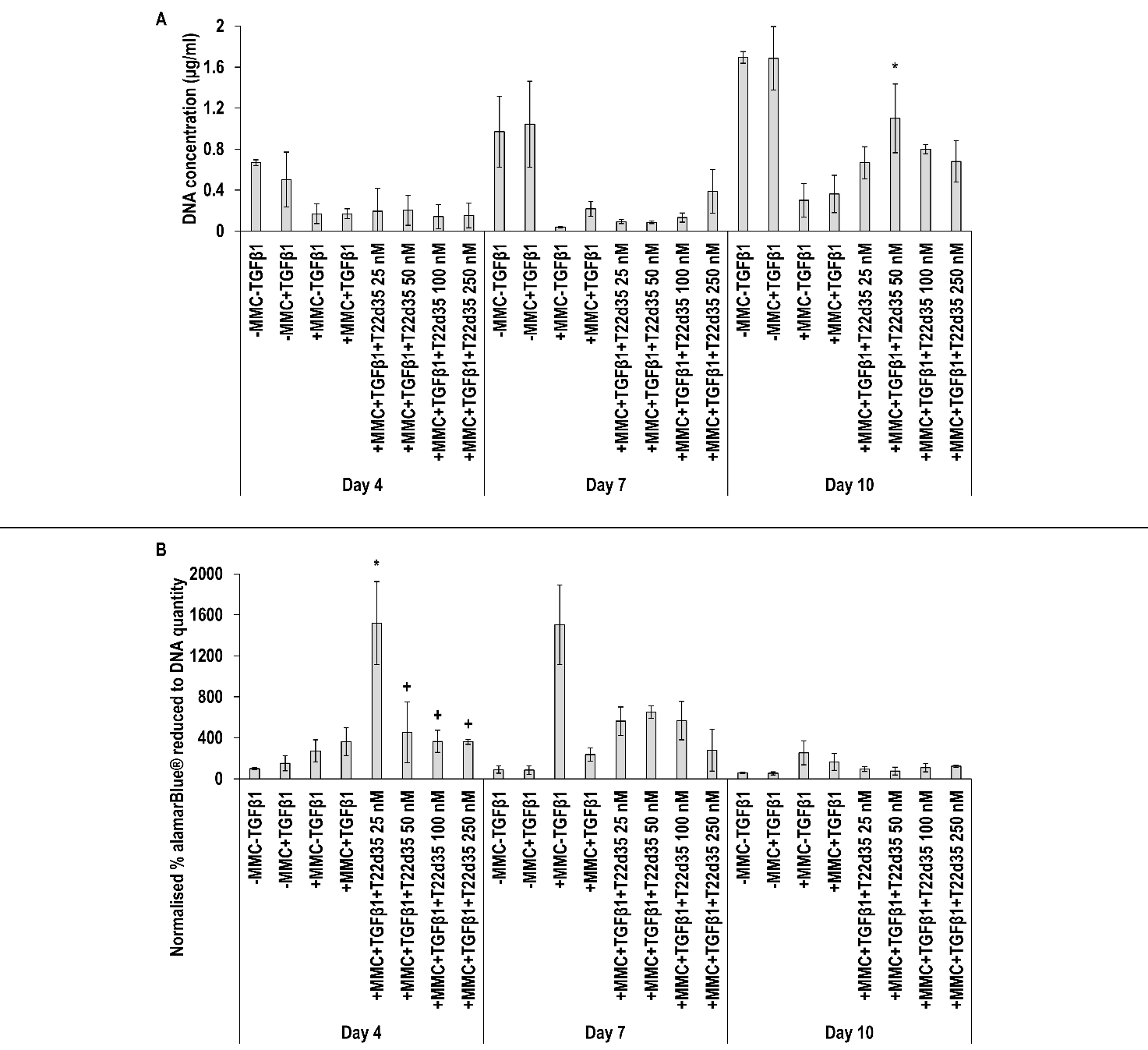


**Supplementary Figure S16:** TGF*β* type II receptor-based trap (T22d35) mostly does not affect cell proliferation and metabolic activity. DNA concentration (A) and metabolic activity (B) were significantly affected, in comparison to the +MMC+TGF*β*1 group, only when the 50 nM T22d35 in +MMC+TGF*β*1 at day 10 and the 25 nM T22d35 in +MMC+TGF*β*1 at day 4, respectively, were used. One-way ANOVA and Tukey’s post-hoc comparison tests were conducted. *: p < 0.05 indicates a statistically significant difference when compared to the +MMC+TGF*β*1 group of the respective time point. +: p < 0.05 indicates a statistically significant difference when compared to the +MMC+TGF*β*1+T22d35 25 nM group of the respective time point. n = 3.


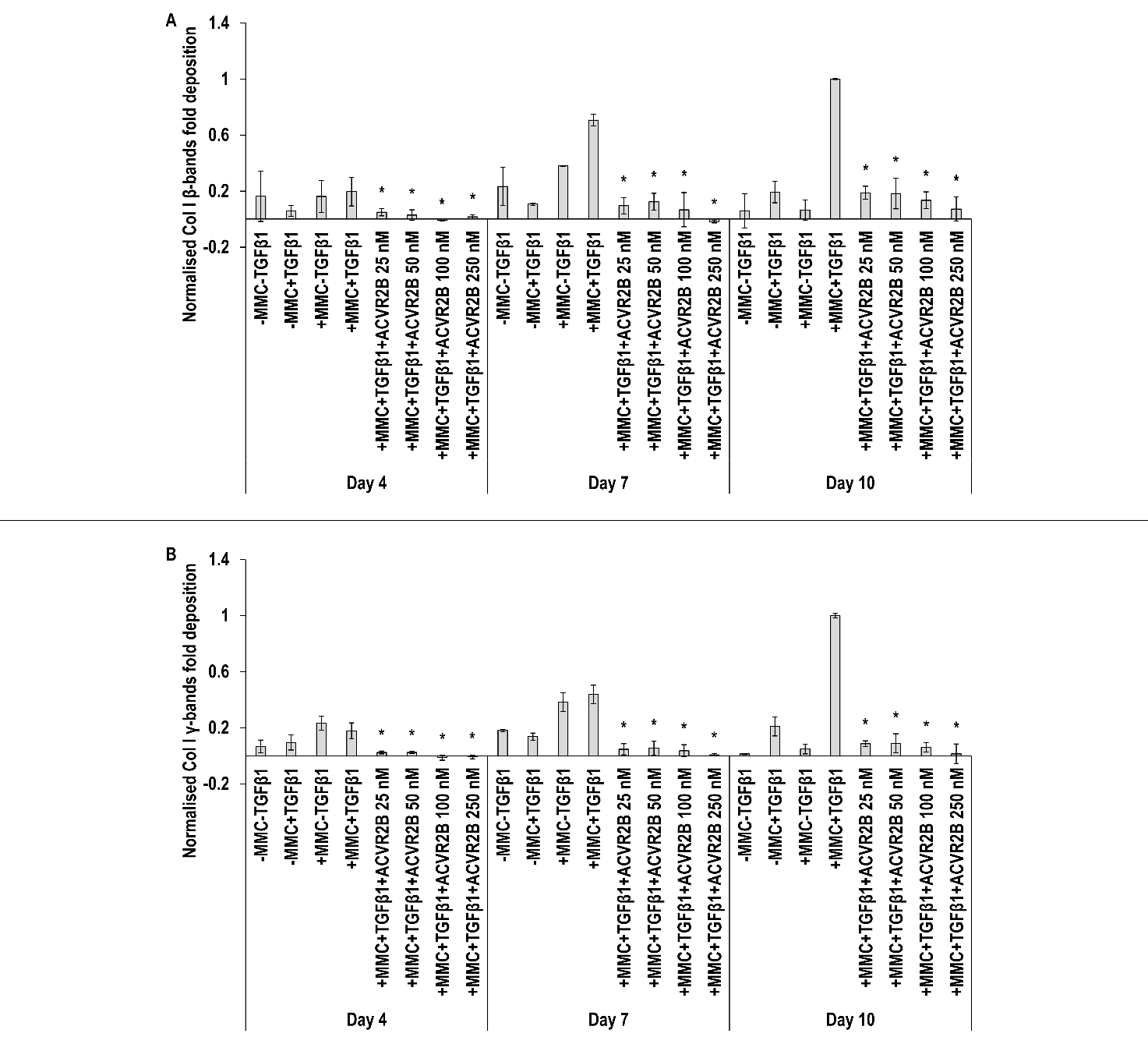


**Supplementary Figure S17:** ACVR2B reduces collagen deposition. All ACVR2B concentrations in +MMC+TGF*β*1 at all time points significantly reduced collagen deposition, in comparison to +MMC+TGF*β*1 group, as judged by densitometry analysis of *β*11(I), *β*12(I) (A) and γ(I) (B) bands. Col I STD: 0.1 mg/ml. One-way ANOVA and Tukey’s post-hoc comparison tests or Kruskal Wallis and Mann Whitney post-hoc analyses were conducted, as appropriate. *: p < 0.05 indicates a statistically significant difference when compared to the +MMC+TGF*β*1 group of the respective time point. n = 3.


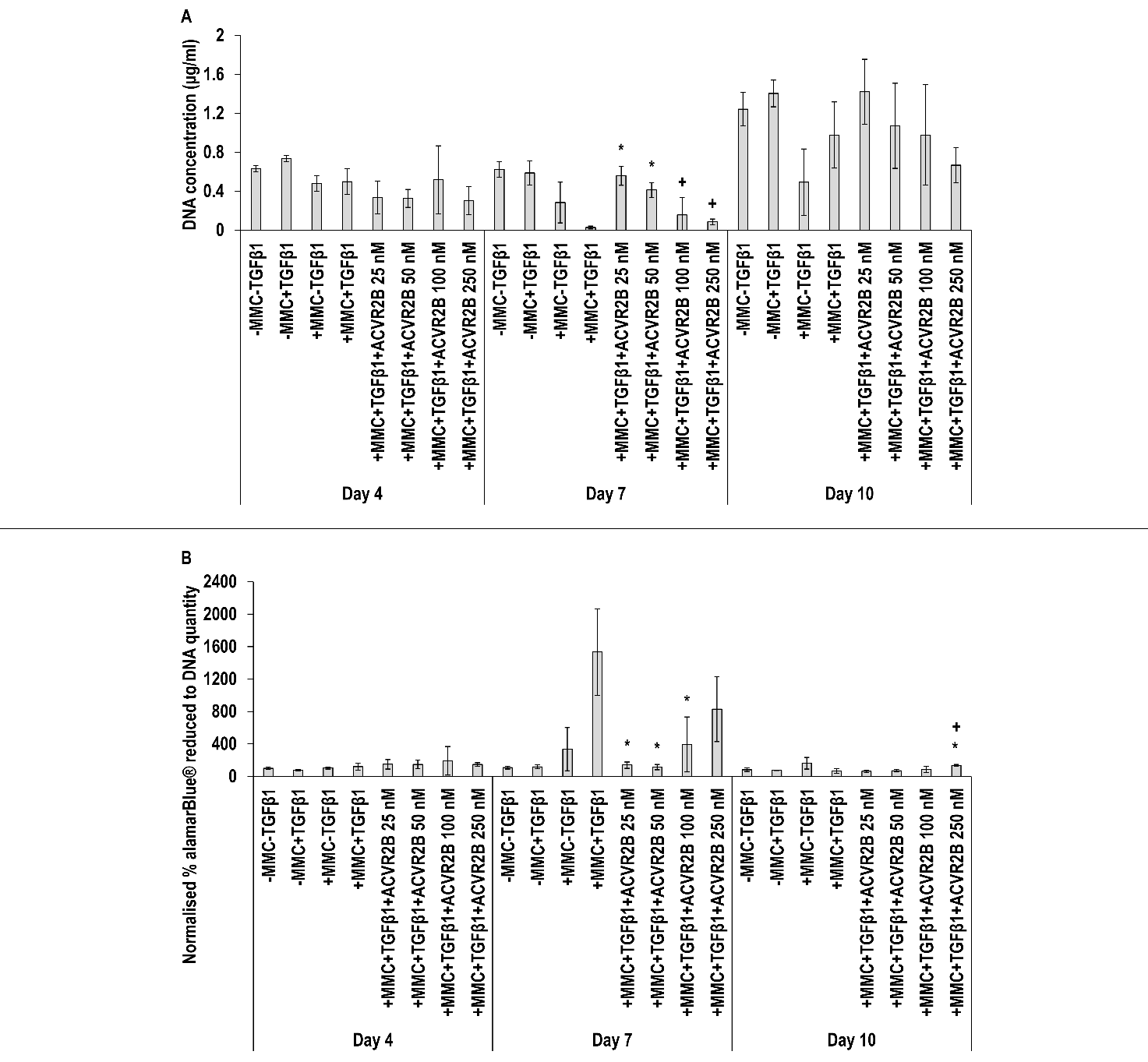


**Supplementary Figure S18:** ACVR2B affects cell proliferation and metabolic activity. The 25 nM and 50 nM AI concentrations in +MMC+TGFβ1 significantly increased DNA concentration (A) in comparison to +MMC+TGFβ1 group, at day 7. The 25 nM, 50 nM and 100 nM AI concentrations in +MMC+TGFβ1 significantly decreased metabolic activity (B), in comparison to +MMC+TGFβ1 group, at day 7 and the 250 nM concentration at day 10. One-way ANOVA and Tukey’s post-hoc comparison tests were conducted. *: p < 0.05 indicates a statistically significant difference when compared to the +MMC+TGFβ1 group of the respective time point. +: p < 0.05 indicates a statistically significant difference when compared to the +MMC+TGFβ1+AI 25 nM group of the respective time point. n = 3.


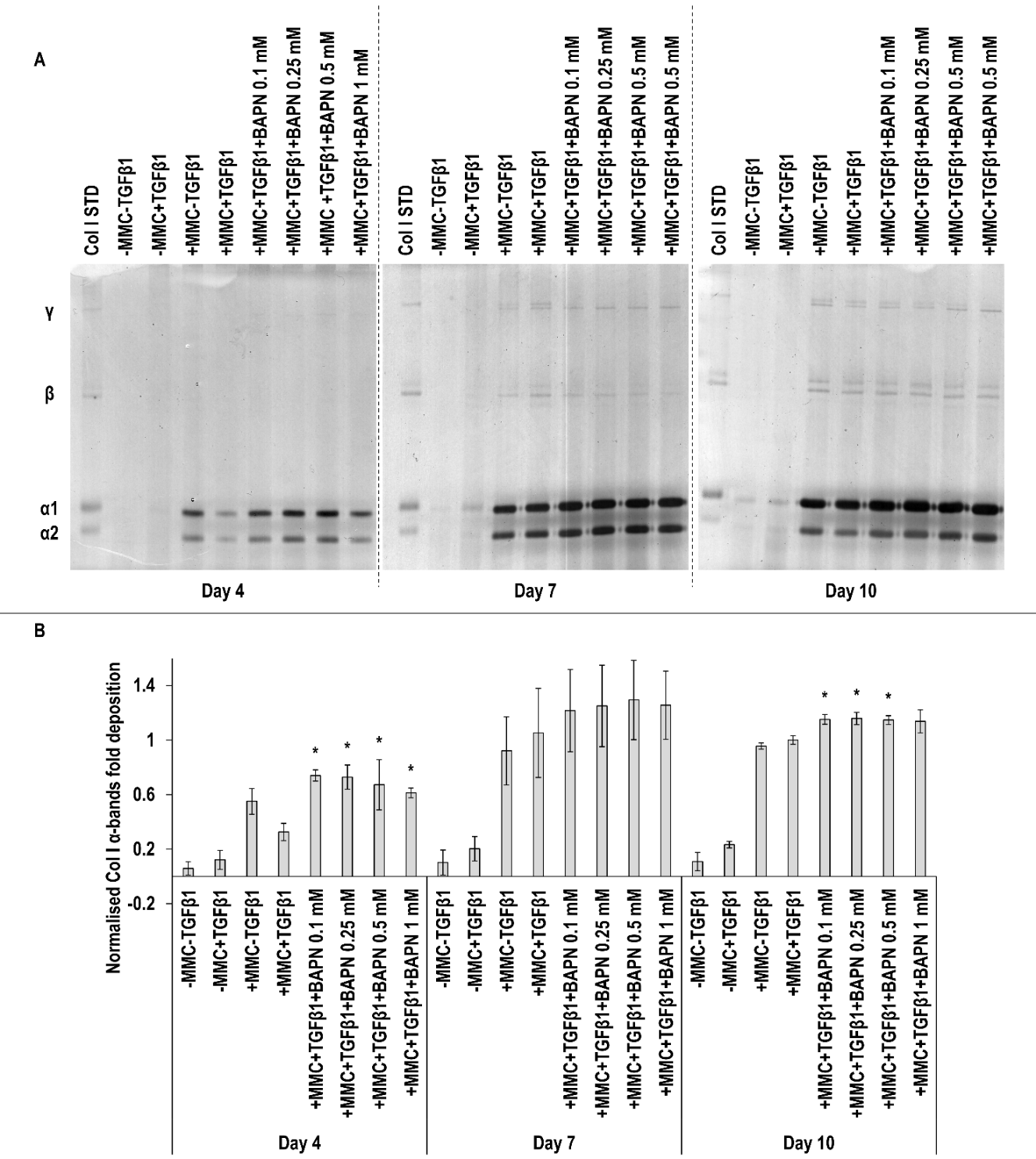


**Supplementary Figure S19:** BAPN does not reduce collagen deposition. All BAPN concentrations in +MMC+TGF*β*1 at day 4 and the concentrations of 0.1 mM, 0.25 mM and 0.5 mM at day 10 significantly increased the deposition of α(I)1 and α(I)2 components, in comparison to +MMC+TGF*β*1 group, as judged by SDS-PAGE (A) and densitometry analysis of α(I)1 and α(I)2 bands (B). Col I STD: 0.1 mg/ml. One-way ANOVA and Tukey’s post-hoc comparison tests. *: p < 0.05 indicates a statistically significant difference when compared to the +MMC+TGF*β*1 group of the respective time point. n = 3.


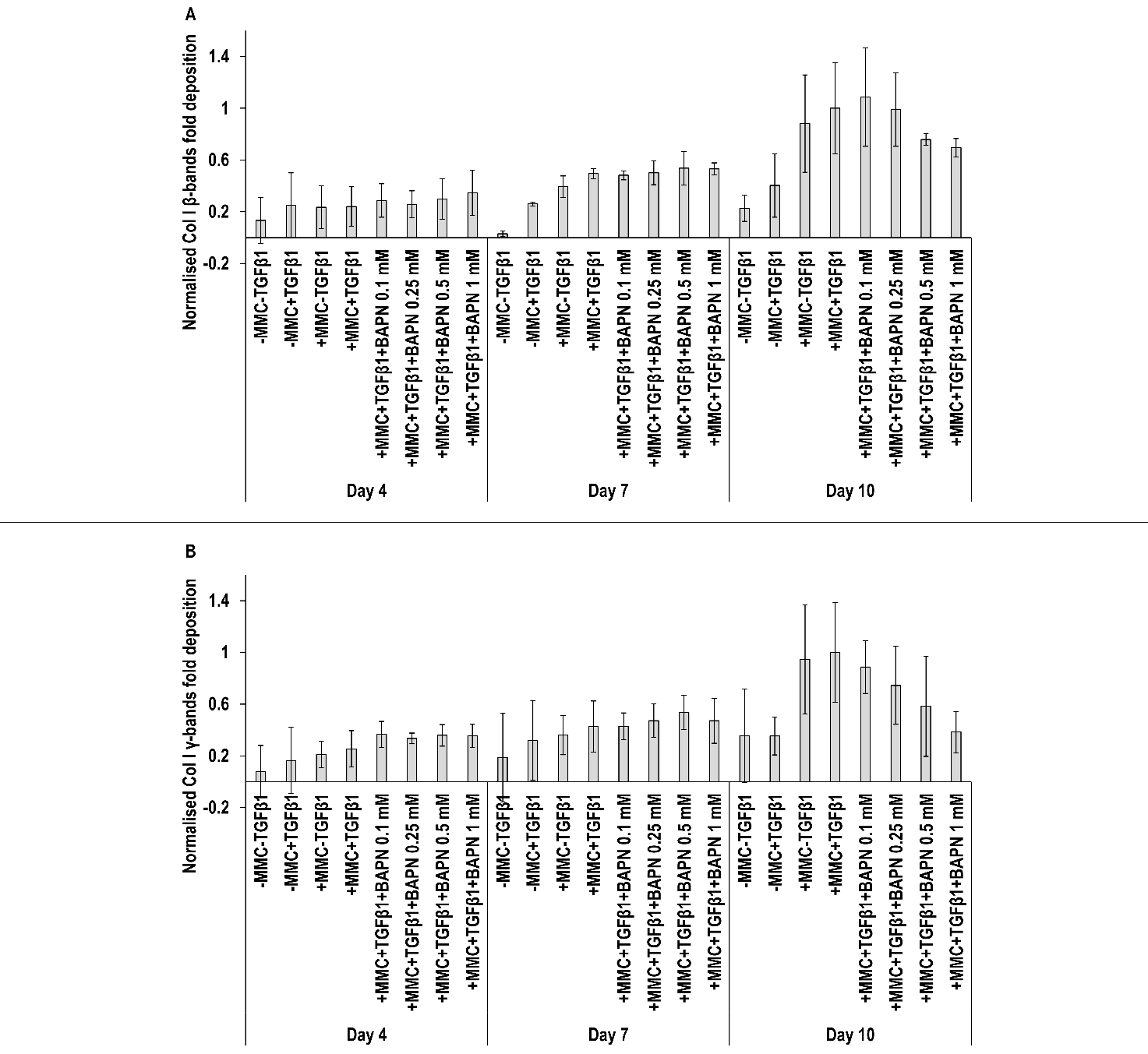


**Supplementary Figure S20:** BAPN does not reduce collagen deposition. No significant differences were observed in collagen I deposition, as judged by densitometry analysis of *β*11(I), *β*12(I) (A) and γ(I) bands (B). Col I STD: 0.1 mg/ml. One-way ANOVA and Tukey’s post-hoc comparison tests. n = 3.


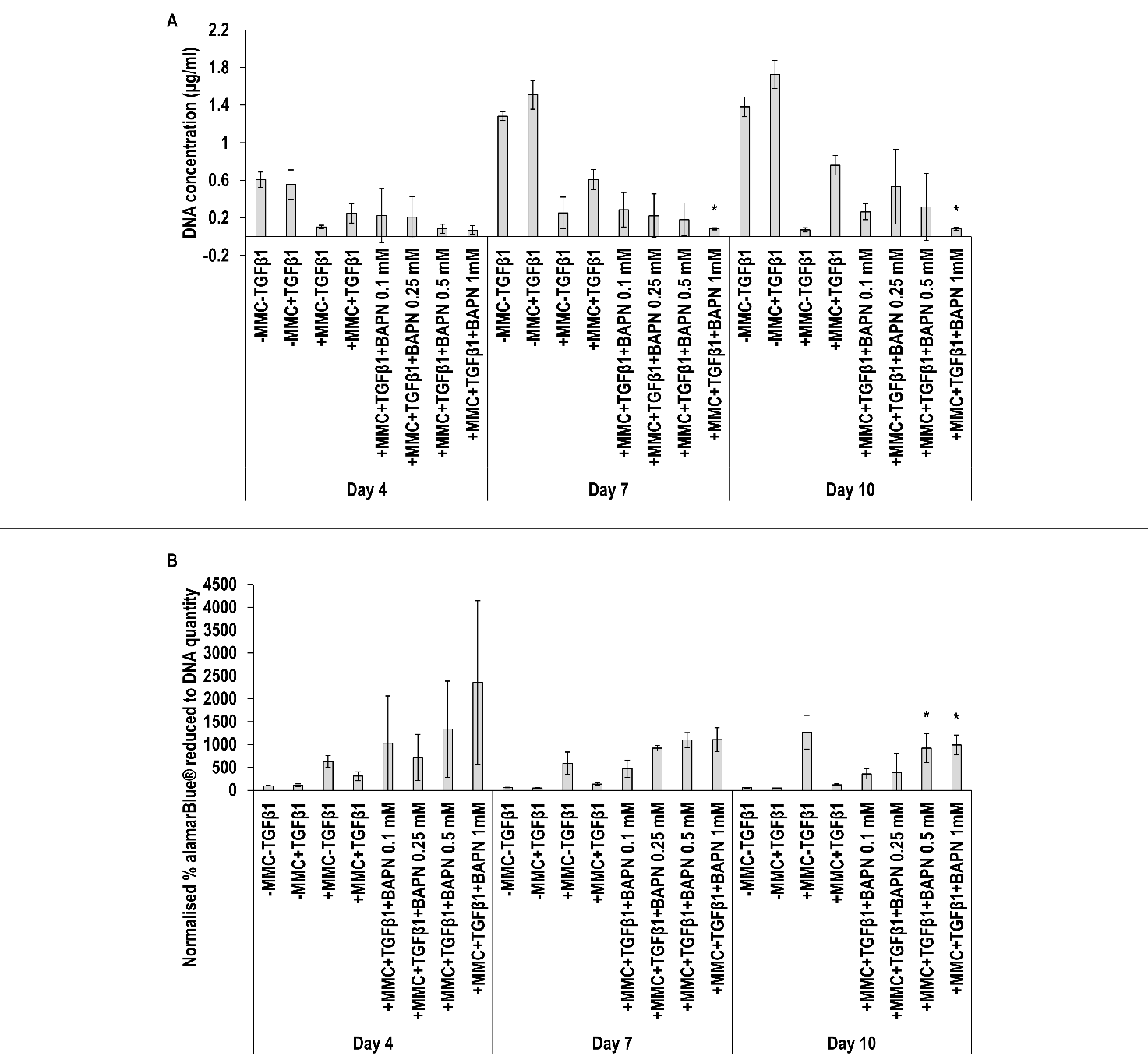


**Supplementary Figure S21:** 1 mM BAPN affects cell proliferation and metabolic activity. The 1 mM concentration of BAPN in +MMC+TGFβ1 significantly reduced DNA concentration (A) and significantly increased metabolic activity (B) in comparison to the +MMC+TGFβ1 group at day 10. One-way ANOVA and Tukey’s post-hoc comparison tests were conducted. *: p < 0.05 indicates a statistically significant difference when compared to the +MMC+TGFβ1 group of the respective time point. n = 3.


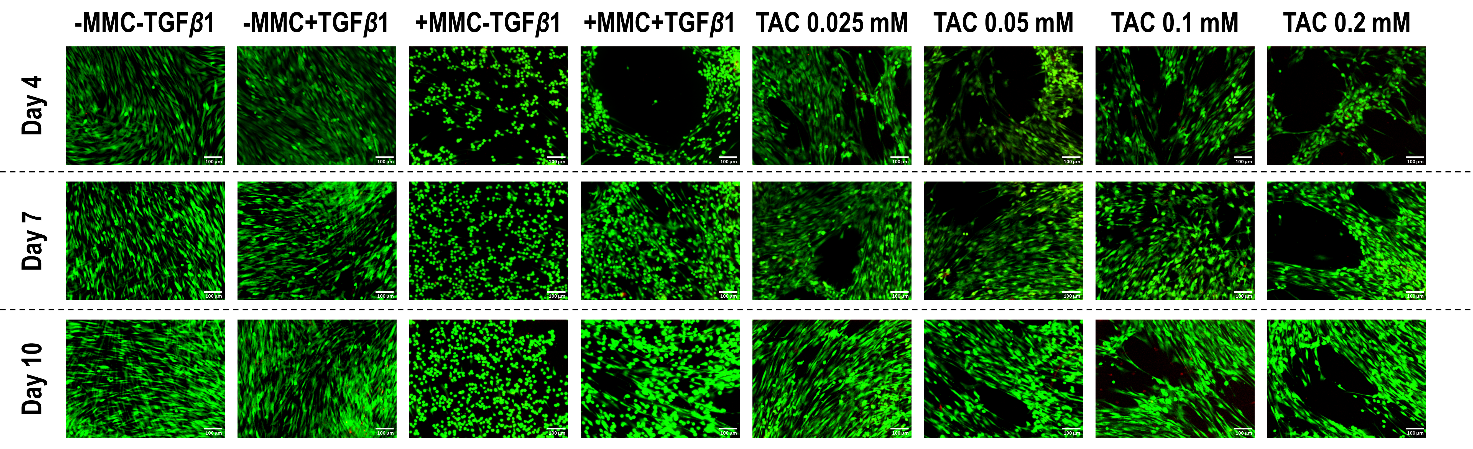


**Supplementary Figure S22:** TAC does not affect cell viability. Analysis of cell viability through calcein AM (green, viable cells) and ethidium homodimer (red, non-viable cells) fluorescence staining after 4, 7 and 10 days in culture and supplementation with +MMC+TGF*β*1 and with TAC at varying concentrations. n = 3.


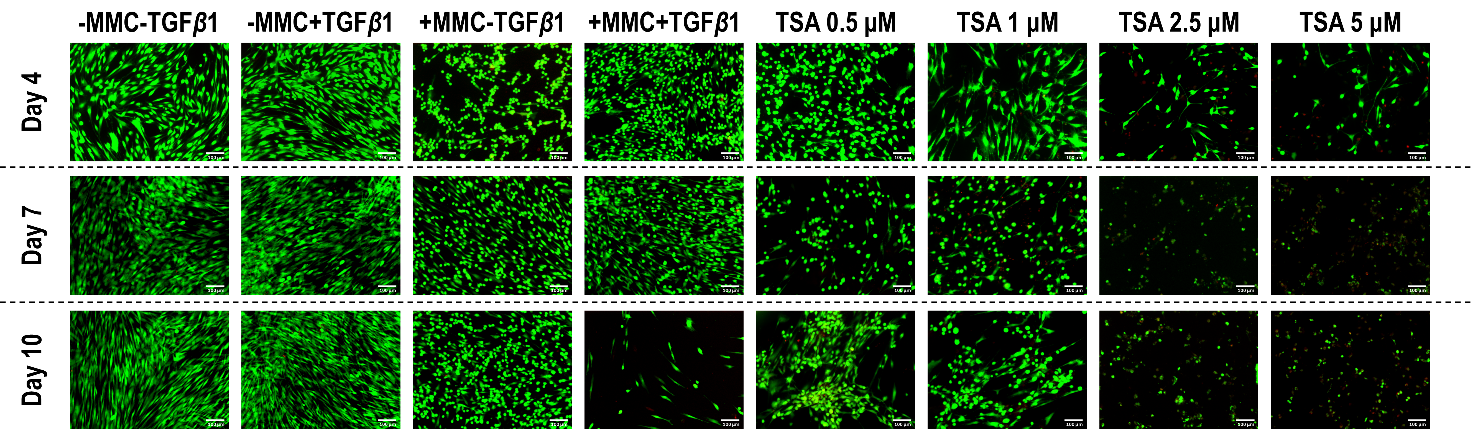


**Supplementary Figure S23:** TSA hinders cell viability at high concentrations (2.5 and 5μM). Analysis of cell viability through calcein AM (green, viable cells) and ethidium homodimer (red, non-viable cells) fluorescence staining after 4, 7 and 10 days in culture and supplementation with +MMC+TGF*β*1 and with TSA at varying concentrations. n = 3.


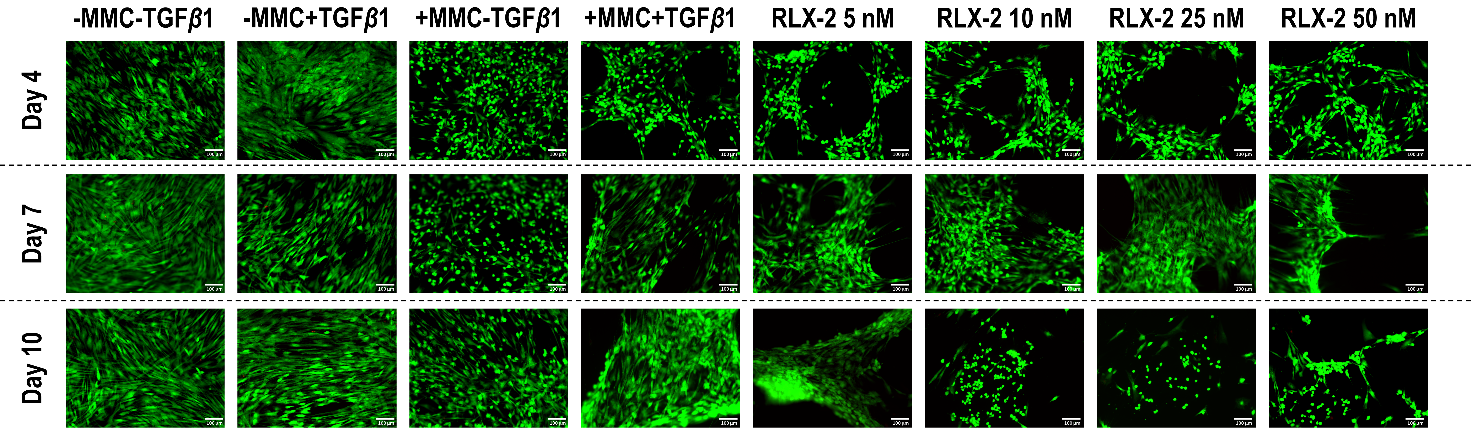


**Supplementary Figure S24:** RLX-2 hinders cell viability at day 10. Analysis of cell viability through calcein AM (green, viable cells) and ethidium homodimer (red, non-viable cells) fluorescence staining after 4, 7 and 10 days in culture and supplementation with +MMC+TGF*β*1 and with RLX-2 at varying concentrations. n = 3.


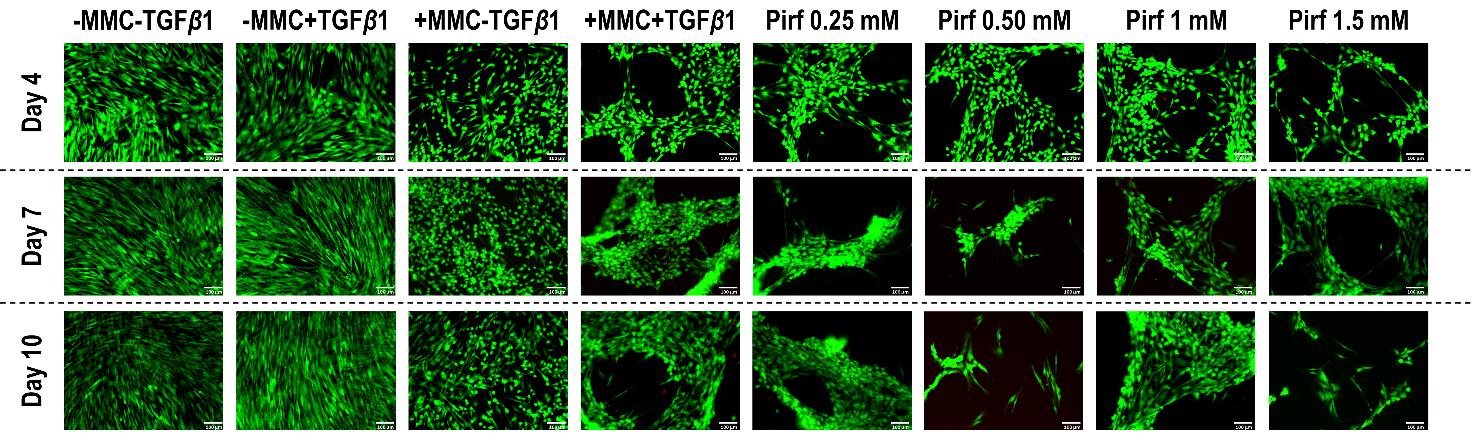


**Supplementary Figure S25:** Pirf affects cell viability at day 7 and day 10. Analysis of cell viability through calcein AM (green, viable cells) and ethidium homodimer (red, non-viable cells) fluorescence staining after 4, 7 and 10 days in culture and supplementation with +MMC+TGFβ1 and with Pirf at varying concentrations. n = 3.


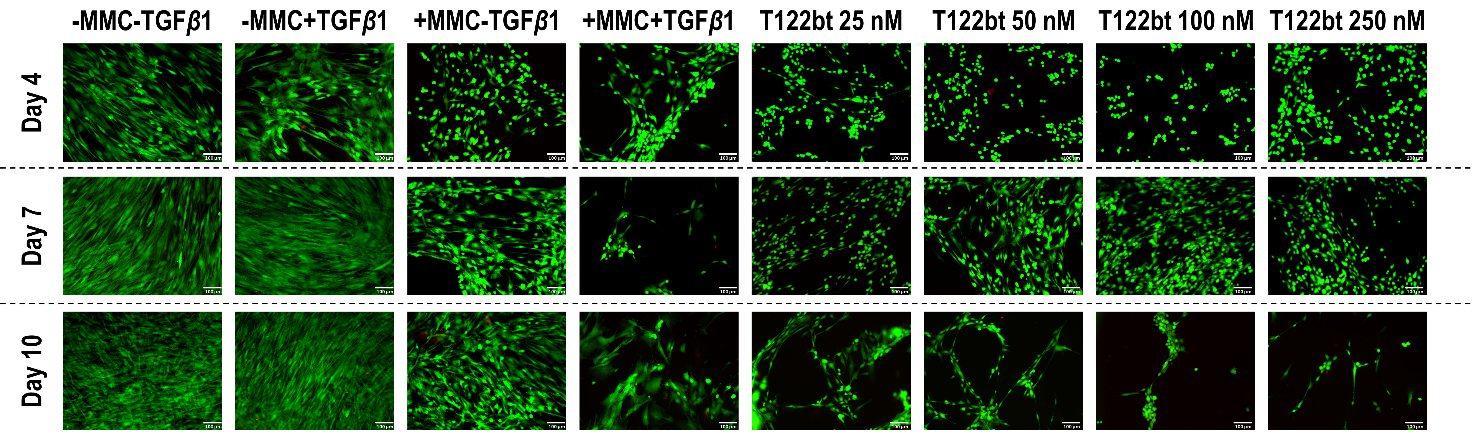


**Supplementary Figure S26:** TGF*β* trap T122bt mildly affects cell viability at day 10. Analysis of cell viability through calcein AM (green, viable cells) and ethidium homodimer (red, non-viable cells) fluorescence staining after 4, 7 and 10 days in culture and supplementation with +MMC+TGF*β*1 and with TGF*β* trap T122bt at varying concentrations. n = 3.


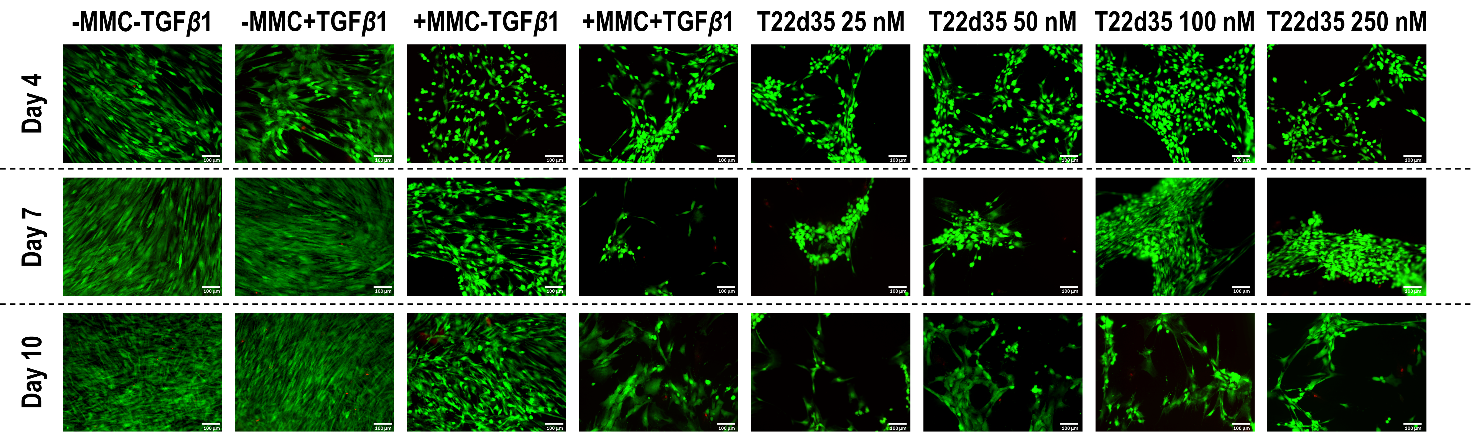


**Supplementary Figure S27:** TGF*β* trap T22d35 did not considerably hinder cell viability. Analysis of cell viability through calcein AM (green, viable cells) and ethidium homodimer (red, non-viable cells) fluorescence staining after 4, 7 and 10 days in culture and supplementation with +MMC+TGF*β*1 and with TGF*β* trap T22d35 at varying concentrations. n = 3.


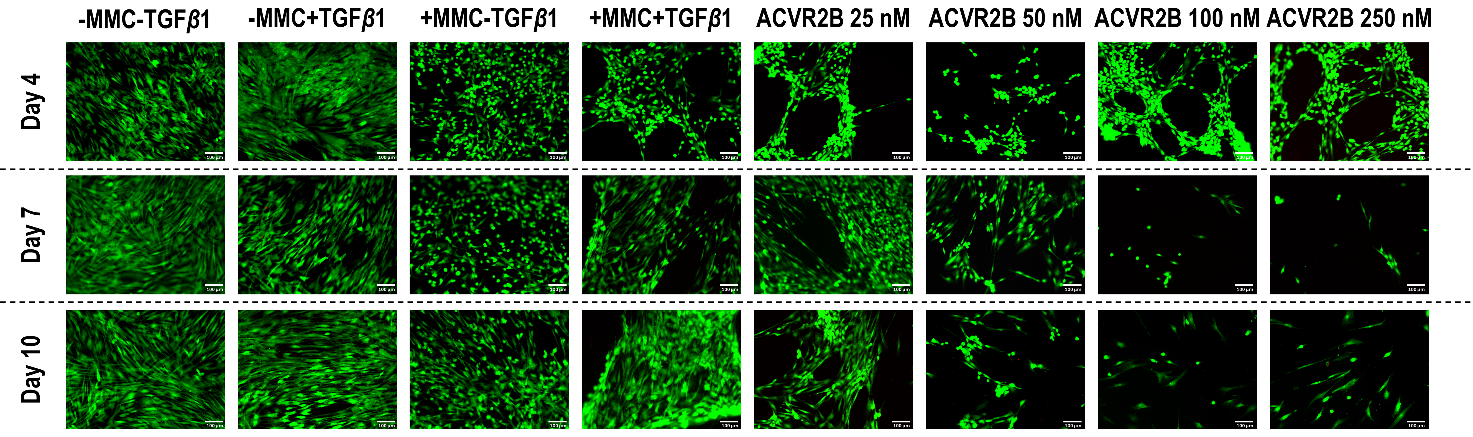


**Supplementary Figure S28:** ACV2R negatively affects cell viability at day 7 and 10. Analysis of cell viability through calcein AM (green, viable cells) and ethidium homodimer (red, non-viable cells) fluorescence staining after 4, 7 and 10 days in culture and supplementation with +MMC+TGF*β*1 and with ACV2R-Fc at varying concentrations. n = 3.


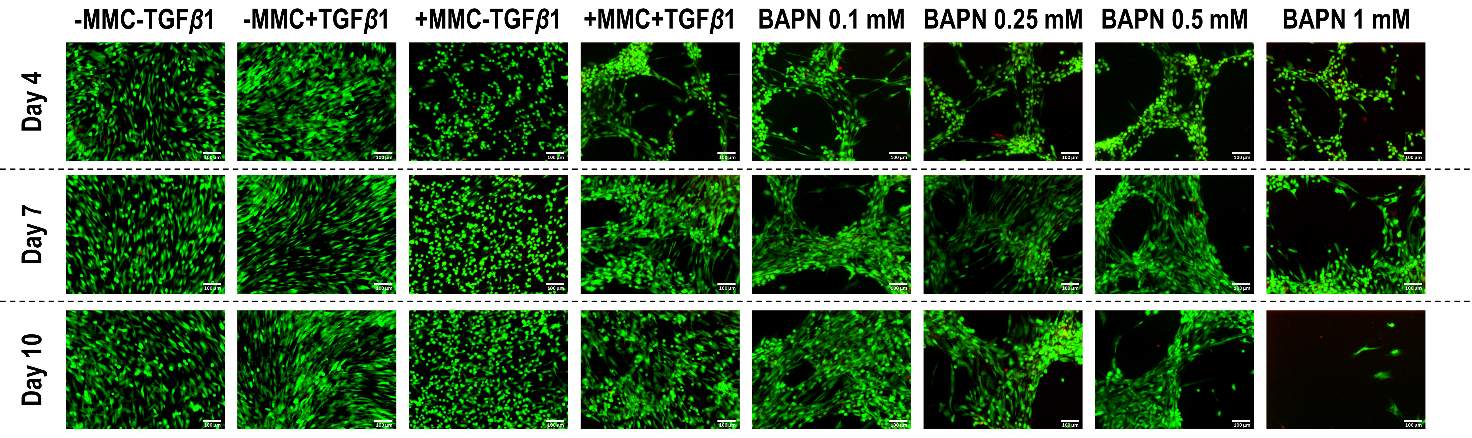


**Supplementary Figure S29:** BAPN hinders cell viability at higher concentrations (0.5 and 1 mM). Analysis of cell viability through calcein AM (green, viable cells) and ethidium homodimer (red, non-viable cells) fluorescence staining after 4, 7 and 10 days in culture and supplementation with +MMC+TGF*β*1 and with BAPN at varying concentrations. n = 3.

**References**

[1] Z.Y. Jiang, X.C. Liao, M.Z. Liu, Z.H. Fu, D.H. Min, G.H. Guo, The safety and efficacy of intralesional verapamil versus intralesional triamcinolone acetonide for keloids and hypertrophic scars: A systematic review and meta-analysis, Adv Skin Wound Care, 33 (2020) 1-7.

[2] K.E. Hietanen, T.A. Järvinen, H. Huhtala, T.T. Tolonen, H.O. Kuokkanen, I.S. Kaartinen, Treatment of keloid scars with intralesional triamcinolone and 5-fluorouracil injections - a randomized controlled trial, J Plast Reconstr Aesthet Surg, 72 (2019) 4-11.

[3] P. Yuan, X. Qiu, T. Liu, R. Tian, Y. Bai, S. Liu, X. Chen, Y. Jin, Substrate-independent polymer coating with stimuli-responsive dexamethasone release for on-demand fibrosis inhibition, J Mater Chem B, 8 (2020) 7777-7784.

[4] F. Syed, S. Singh, A. Bayat, Superior effect of combination vs. single steroid therapy in keloid disease: A comparative in vitro analysis of glucocorticoids, Wound Repair Regen, 21 (2013) 88-102.

[5] L.C. Huber, J.H. Distler, F. Moritz, H. Hemmatazad, T. Hauser, B.A. Michel, R.E. Gay, M. Matucci-Cerinic, S. Gay, O. Distler, A. Jüngel, Trichostatin A prevents the accumulation of extracellular matrix in a mouse model of bleomycin-induced skin fibrosis, Arthritis Rheum, 56 (2007) 2755-2764.

[6] M.B. Ruffy, S.S. Kunnavatana, R.J. Koch, Effects of tamoxifen on normal human dermal fibroblasts, Arch Facial Plast Surg, 8 (2006) 329-332.

[7] L.R. Soares-Lopes, I.M. Soares-Lopes, L.L. Filho, A.P. Alencar, B.B. da Silva, Morphological and morphometric analysis of the effects of intralesional tamoxifen on keloids, Exp Biol Med, 242 (2017) 926-929.

[8] R.A. Clark, G.A. McCoy, J.M. Folkvord, J.M. McPherson, TGF-beta 1 stimulates cultured human fibroblasts to proliferate and produce tissue-like fibroplasia: A fibronectin matrix-dependent event, J Cell Physiol, 170 (1997) 69-80.

[9] P. Wang, L. Gu, H. Bi, Q. Wang, Z. Qin, Comparing the efficacy and safety of intralesional verapamil with intralesional triamcinolone acetonide in treatment of hypertrophic scars and keloids: A meta-analysis of randomized controlled trials, Aesthet Surg J, 41 (2021) Np567-np575.

[10] W.J. Lee, H.M. Ahn, H. Roh, Y. Na, I.K. Choi, J.H. Lee, Y.O. Kim, D.H. Lew, C.O. Yun, Decorin-expressing adenovirus decreases collagen synthesis and upregulates MMP expression in keloid fibroblasts and keloid spheroids, Exp Dermatol, 24 (2015) 591-597.

[11] C. McArdle, S.A. Abbah, S. Bhowmick, E. Collin, A. Pandit, Localized temporal co-delivery of interleukin 10 and decorin genes using amediated by collagen-based biphasic scaffold modulates the expression of TGF-β1/β2 in a rabbit ear hypertrophic scarring model, Biomaterials science, 9 (2021) 3136-3149.

[12] L. Pan, H. Qin, C. Li, L. Yang, M. Li, J. Kong, G. Zhang, L. Zhang, Safety and efficacy of botulinum toxin type A in preventing and treating scars in animal models: A systematic review and meta-analysis, Int Wound J, (2021) doi: 10.1111/iwj.13673.

[13] A.R. Elshahed, K.S. Elmanzalawy, H. Shehata, M.L. ElSaie, Effect of botulinum toxin type A for treating hypertrophic scars: A split-scar, double-blind randomized controlled trial, J Cosmet Dermatol, 19 (2020) 2252-2258.

[14] Y.L. Huang, C.G. Wallace, Y.C. Hsiao, M.C. Lee, J.J. Huang, F.C. Chang, Z.C. Chen, S. Hu, J.P. Chen, Botulinum toxin to improve lower blepharoplasty scar: A double-blinded, randomized, vehicle-controlled clinical trial, Aesthetic surgery journal, (2021) sjab024.

[15] D. Khanna, P.J. Clements, D.E. Furst, J.H. Korn, M. Ellman, N. Rothfield, F.M. Wigley, L.W. Moreland, R. Silver, Y.H. Kim, V.D. Steen, G.S. Firestein, A.F. Kavanaugh, M. Weisman, M.D. Mayes, D. Collier, M.E. Csuka, R. Simms, P.A. Merkel, T.A. Medsger, Jr., M.E. Sanders, P. Maranian, J.R. Seibold, Recombinant human relaxin in the treatment of systemic sclerosis with diffuse cutaneous involvement: A randomized, double-blind, placebo-controlled trial, Arthritis Rheum, 60 (2009) 1102-1111.

[16] C. Corallo, A.M. Pinto, A. Renieri, S. Cheleschi, A. Fioravanti, M. Cutolo, S. Soldano, R. Nuti, N. Giordano, Altered expression of RXFP1 receptor contributes to the inefficacy of relaxin-based anti-fibrotic treatments in systemic sclerosis, Clin Exp Rheumatol, 37 Suppl 119 (2019) 69-75.

[17] M. Saito, M. Yamazaki, T. Maeda, H. Matsumura, Y. Setoguchi, R. Tsuboi, Pirfenidone suppresses keloid fibroblast-embedded collagen gel contraction, Arch Dermatol Res, 304 (2012) 217-222.

[18] C.D.D.L.d.l. Pascua, Efficacy of Intralesional Triamcinolone and 8% Topical Pirfenidone for Treatment of Keloid Scars (LADISLAO) (NCT02823236), ClinicalTrials.gov, 2017.

[19] Z. Tamaki, Y. Asano, M. Kubo, H. Ihn, Y. Tada, M. Sugaya, T. Kadono, S. Sato, Effects of the immunosuppressant rapamycin on the expression of human α2(I) collagen and matrix metalloproteinase 1 genes in scleroderma dermal fibroblasts, J Dermatol Sci, 74 (2014) 251-259.

[20] A. Yoshizaki, K. Yanaba, A. Yoshizaki, Y. Iwata, K. Komura, F. Ogawa, M. Takenaka, K. Shimizu, Y. Asano, M. Hasegawa, M. Fujimoto, S. Sato, Treatment with rapamycin prevents fibrosis in tight-skin and bleomycin-induced mouse models of systemic sclerosis, Arthritis Rheum, 62 (2010) 2476-2487.

[21] P.L. Chen, J.B. Hong, L.J. Shen, Y.T. Chen, S.J. Wang, Y.H. Liao, The efficacy and safety of topical rapamycin-calcitriol for facial angiofibromas in patients with tuberous sclerosis complex: A prospective, double-blind, randomized clinical trial, Br J Dermatol, 183 (2020) 655-663.

[22] G.R. Botstein, G.K. Sherer, E.C. Leroy, Fibroblast selection in scleroderma. An alternative model of fibrosis, Arthritis Rheum, 25 (1982) 189-195.

[23] M. Calderon, W.T. Lawrence, A.J. Banes, Increased proliferation in keloid fibroblasts wounded in vitro, J Surg Res, 61 (1996) 343-347.

[24] C. Tsai, K. Hata, S. Torii, M. Matsuyama, M. Ueda, Contraction potency of hypertrophic scar-derived fibroblasts in a connective tissue model: In vitro analysis of wound contraction, Ann Plast Surg, 35 (1995) 638-646.

[25] P. Smith, G. Mosiello, L. Deluca, F. Ko, S. Maggi, M.C. Robson, TGF-beta2 activates proliferative scar fibroblasts, J Surg Res, 82 (1999) 319-323.

[26] C. Fan, L.K.P. Lim, S.Q. Loh, K.Y. Ying Lim, Z. Upton, D. Leavesley, Application of "macromolecular crowding" in vitro to investigate the naphthoquinones shikonin, naphthazarin and related analogues for the treatment of dermal scars, Chem Biol Interact, 310 (2019) 108747.

[27] C. Fan, L.K.P. Lim, Z. Wu, B. Sharma, S.Q. Gan, K. Liang, Z. Upton, D. Leavesley, In vitro model of human cutaneous hypertrophic scarring using macromolecular crowding, J Vis Exp, (2020) doi: 10.3791/61037.

[28] C.W. Wong, C.F. LeGrand, B.F. Kinnear, R.M. Sobota, R. Ramalingam, D.E. Dye, M. Raghunath, E.B. Lane, D.R. Coombe, In vitro expansion of keratinocytes on human dermal fibroblast-derived matrix retains their stem-like characteristics, Sci Rep, 9 (2019) 18561.

[29] M. Lebeko, N.P. Khumalo, A. Bayat, Multi-dimensional models for functional testing of keloid scars: In silico, in vitro, organoid, organotypic, ex vivo organ culture, and in vivo models, Wound Repair Regen, 27 (2019) 298-308.

[30] D. Suttho, S. Mankhetkorn, D. Binda, L. Pazart, P. Humbert, G. Rolin, 3D modeling of keloid scars in vitro by cell and tissue engineering, Arch Dermatol Res, 309 (2017) 55-62.

[31] M.C. Vozenin, J.L. Lefaix, R. Ridi, D.S. Biard, F. Daburon, M. Martin, The myofibroblast markers α-SM actin and β-actin are differentially expressed in 2 and 3-D culture models of fibrotic and normal skin, Cytotechnology, 26 (1998) 29-38.

[32] L.J. van den Broek, F.B. Niessen, R.J. Scheper, S. Gibbs, Development, validation and testing of a human tissue engineered hypertrophic scar model, Altex, 29 (2012) 389-402.

[33] L.J. van den Broek, G.C. Limandjaja, F.B. Niessen, S. Gibbs, Human hypertrophic and keloid scar models: Principles, limitations and future challenges from a tissue engineering perspective, Exp Dermatol, 23 (2014) 382-386.

[34] J. Li, J. Wang, Z. Wang, Y. Xia, M. Zhou, A. Zhong, J. Sun, Experimental models for cutaneous hypertrophic scar research, Wound Repair Regen, 28 (2020) 126-144.

[35] L. Bergers, C.M.A. Reijnders, L.J. van den Broek, S.W. Spiekstra, T.D. de Gruijl, E.M. Weijers, S. Gibbs, Immune-competent human skin disease models, Drug Discov Today, 21 (2016) 1479-1488.

[36] S. Ud-Din, A. Bayat, Non-animal models of wound healing in cutaneous repair: In silico, in vitro, ex vivo, and in vivo models of wounds and scars in human skin, Wound Repair Regen, 25 (2017) 164-176.

[37] A.E. Matei, C.W. Chen, L. Kiesewetter, A.H. Györfi, Y.N. Li, T. Trinh-Minh, X. Xu, C. Tran Manh, T. van Kuppevelt, J. Hansmann, A. Jüngel, G. Schett, F. Groeber-Becker, J.H.W. Distler, Vascularised human skin equivalents as a novel in vitro model of skin fibrosis and platform for testing of antifibrotic drugs, Ann Rheum Dis, 78 (2019) 1686-1692.

[38] H.S. Duong, Q. Zhang, A. Kobi, A. Le, D.V. Messadi, Assessment of morphological and immunohistological alterations in long-term keloid skin explants, Cells Tissues Organs, 181 (2005) 89-102.

[39] R. Bagabir, F. Syed, R. Paus, A. Bayat, Long-term organ culture of keloid disease tissue, Exp Dermatol, 21 (2012) 376-381.

[40] K.W. Ng, M. Pearton, S. Coulman, A. Anstey, C. Gateley, A. Morrissey, C. Allender, J. Birchall, Development of an ex vivo human skin model for intradermal vaccination: Tissue viability and Langerhans cell behaviour, Vaccine, 27 (2009) 5948-5955.

[41] D.A. Burd, M.T. Longaker, N.S. Adzick, C.C. Compton, M.R. Harrison, J.W. Siebert, H.P. Ehrlich, Fetal wound healing: An in vitro explant model, J Pediatr Surg, 25 (1990) 898-901.

[42] W.J. Lee, I.K. Choi, J.H. Lee, Y.O. Kim, C.O. Yun, A novel three-dimensional model system for keloid study: Organotypic multicellular scar model, Wound Repair Regen, 21 (2013) 155-165.

[43] W.J. Lee, J.H. Lee, H.M. Ahn, S.Y. Song, Y.O. Kim, D.H. Lew, C.O. Yun, Heat shock protein 90 inhibitor decreases collagen synthesis of keloid fibroblasts and attenuates the extracellular matrix on the keloid spheroid model, Plast Reconstr Surg, 136 (2015) 328e-337e.

[44] K. Schimek, H.H. Hsu, M. Boehme, J.J. Kornet, U. Marx, R. Lauster, R. Pörtner, G. Lindner, Bioengineering of a full-thickness skin equivalent in a 96-well insert format for substance permeation studies and organ-on-a-chip applications, Bioengineering, 5 (2018) 43.

[45] N. Mori, Y. Morimoto, S. Takeuchi, Skin integrated with perfusable vascular channels on a chip, Biomaterials, 116 (2017) 48-56.

[46] H.E. Abaci, Z. Guo, Y. Doucet, J. Jacków, A. Christiano, Next generation human skin constructs as advanced tools for drug development, Exp Biol Med, 242 (2017) 1657-1668.

[47] S. Lee, S.P. Jin, Y.K. Kim, G.Y. Sung, J.H. Chung, J.H. Sung, Construction of 3D multicellular microfluidic chip for an in vitro skin model, Biomed Microdevices, 19 (2017) 22.

[48] M.H. Mohammadi, B. Heidary Araghi, V. Beydaghi, A. Geraili, F. Moradi, P. Jafari, M. Janmaleki, K.P. Valente, M. Akbari, A. Sanati-Nezhad, Skin diseases modeling using combined tissue engineering and microfluidic technologies, Adv Healthc Mater, 5 (2016) 2459-2480.

[49] J.C. Zwaagstra, T. Sulea, J. Baardsnes, A.E. Lenferink, C. Collins, C. Cantin, B. Paul-Roc, S. Grothe, S. Hossain, L.P. Richer, D. L'Abbé, R. Tom, B. Cass, Y. Durocher, M.D. O'Connor-McCourt, Engineering and therapeutic application of single-chain bivalent TGF-β family traps, Mol Cancer Ther, 11 (2012) 1477-1487.

[50] M. O'Connor-Mccourt, T. Sulea, J. Zwaagstra, J. Baardsnes, Antagonists of ligands and uses thereof, US8574548B2 (2013).
